# Microarray Analysis of Serum mRNA in Patients with Head and Neck Squamous Cell Carcinoma at Whole-Genome Scale

**DOI:** 10.1155/2014/408683

**Published:** 2014-04-23

**Authors:** Markéta Čapková, Jana Šáchová, Hynek Strnad, Michal Kolář, Miluše Hroudová, Martin Chovanec, Zdeněk Čada, Martin Šteffl, Jaroslav Valach, Jan Kastner, Čestmír Vlček, Karel Smetana, Jan Plzák

**Affiliations:** ^1^Department of Otorhinolaryngology and Head and Neck Surgery, 1st Faculty of Medicine, Charles University, University Hospital Motol, Institute of Postgraduate Medicine, V Uvalu 84, 15006 Prague 5, Czech Republic; ^2^Academy of Sciences of the Czech Republic, Institute of Molecular Genetics, Department of Genomics and Bioinformatics, Videnska 1083, 14220 Prague 4, Czech Republic; ^3^Institute of Anatomy, 1st Faculty of Medicine, Charles University, U Nemocnice 3, 12800 Prague 2, Czech Republic; ^4^Department of Stomatology, 1st Faculty of Medicine, Charles University, Kateřinská 32, 12108 Prague 2, Czech Republic

## Abstract

With the increasing demand for noninvasive approaches in monitoring head and neck cancer, circulating nucleic acids have been shown to be a promising tool. We focused on the global transcriptome of serum samples of head and neck squamous cell carcinoma (HNSCC) patients in comparison with healthy individuals. We compared gene expression patterns of 36 samples. Twenty-four participants including 16 HNSCC patients (from 12 patients we obtained blood samples 1 year posttreatment) and 8 control subjects were recruited. The Illumina HumanWG-6 v3 Expression BeadChip was used to profile and identify the differences in serum mRNA transcriptomes. We found 159 genes to be significantly changed (Storey's *P* value <0.05) between normal and cancer serum specimens regardless of factors including p53 and B-cell lymphoma family members (Bcl-2, Bcl-XL). In contrast, there was no difference in gene expression between samples obtained before and after surgery in cancer patients. We suggest that microarray analysis of serum cRNA in patients with HNSCC should be suitable for refinement of early stage diagnosis of disease that can be important for development of new personalized strategies in diagnosis and treatment of tumours but is not suitable for monitoring further development of disease.

## 1. Introduction

Head and neck squamous cell carcinoma (HNSCC) is the fifth most common cancer worldwide. The overall incidence is half a million cases per year. HNSCC accounts for about 10% of the total cancer burden in men [1]. Its biological behaviour is typified by aggressive locoregional invasiveness, local recurrence, and tumour multiplicity. Despite considerable advances in surgical and oncological treatment over the past two decades, overall disease outcome has only slightly improved. The main reason for this is late diagnosis, with almost two-thirds of cases being diagnosed in the late stage of disease. The presence of lymph node metastasis is associated with a 50% decrease in 5-year survival and is the single most important prognostic factor identified to date [2–5]. For better prognosis of patients and reduction of posttherapeutic morbidity, including significant discomfort, it is crucial to recognise the cancer at an early stage. Therefore, many scientists have dedicated much effort to identification of potential biomarkers involved in the process of carcinogenesis and investigation of the molecular characteristics of HNSCC.

The field of cancer research has continued to evolve rapidly, and, in recent years, there have been advances in our knowledge of the molecular biology and epigenetics of HNSCC as well as in the techniques available to study this disease. Development of high-throughput expression-array-based techniques has led to the detection of novel tumour suppressor genes (TSGs) and protooncogenes, as well as a description of epigenetic modifications involved in tumourigenesis. Finally, the field of bioinformatics is now intimately involved in deciphering the data generated by these techniques. Many studies have yielded promising results in this field by analysing tumour tissue samples in comparison to normal mucosa [6–11]. However, assays based on processing of tumour tissue samples do not resolve the problem of early diagnosis, and invasive tumour biopsy places great demands on the patient and is limited in size.

With the increasing demand for noninvasive approaches in the monitoring of cancer, circulating nucleic acids (CNAs) have been shown to be a promising tool. In the case of neoplasia, circulating RNA has been found to be a more sensitive marker than tumour-derived circulating DNA. The first study associating circulating RNA in the serum as a potential tumour marker was reported by Wieczorek et al. more than 20 years ago. That study reported an association between the presence of the RNA–proteolipid complex and the tumour response to therapy [12]. During the past 10 years, there has been a great leap forward in detecting and testing isolated cell-free RNA for different tumour-related transcripts [13, 14], telomerase components [15], or viral RNA transcripts. Tumour-associated RNA was detectable in the serum or plasma of patients with breast, liver, or lung cancer [16], colorectal cancer [17], follicular lymphoma [18], prostate cancer [19], malignant melanoma [20], hepatocellular carcinoma, oesophageal carcinoma, and others [21, 22]. Unfortunately, almost no data are available on cRNA in patients with HNSCC. The detection and identification of cRNA can be carried out by using microarray technologies or reverse transcription quantitative real-time PCR [23].

Detection of cell-free RNA in plasma and serum could potentially serve as a “liquid biopsy,” which would avoid the need for tumour tissue biopsies. This approach is especially favourable for its possibility of taking repeated blood samples during cancer development and progression, as well as during the monitoring of cancer treatment. Indeed the role of CNAs as blood biomarkers was recently highlighted [24].

## 2. Materials and Methods

Twenty-four participants including 16 HNSCC patients (from 12 patients we obtained blood samples 1 year after treatment) and eight control subjects were recruited. The Illumina HumanWG-6 v3 Expression BeadChip was used to profile and identify the differences in serum mRNA transcriptomes between cancer patients and healthy controls as well as the differences in serum mRNA transcriptomes between serum from the same donor obtained before surgery and 1 year after treatment.

### 2.1. Blood Specimens and Collection Procedure

The blood specimens were obtained from Department of Otorhinolaryngology and Head and Neck Surgery (Charles University, First Faculty of Medicine, Prague, Czech Republic), with patient consent and approval of the Local Ethical Committee according to the principles of the Helsinki Declaration. The blood samples were obtained from patients with HNSCC who underwent surgical treatment and were drawn before surgery and then approximately 1 year after treatment. As healthy controls, we selected patients with benign noninflammatory diagnoses. The demographic and clinical data of cases and controls are shown in Table 1. All blood samples were processed within 2 hours after venous puncture. Blood was centrifuged at 1,000 *g* for 10 minutes at 4°C, and 0.5-mL aliquots of serum samples were stored at −80°C.

### 2.2. RNA Extraction, Amplification, Labeling, and Hybridization

*Extraction.* An aliquot of 400 *μ*L of each serum sample was used for RNA extraction. Total RNA was isolated by MagMAX Viral RNA Isolation Kit (Ambion Inc., Foster City, CA, USA) according to the manufacturer's recommendations. RNA quantity was measured on a NanoDrop 3300 fluorospectrometer (NanoDrop Technologies LLC, Wilmington, DE, USA). RNA integrity was assessed on an Agilent 2100 Bioanalyzer and RNA 6000 Pico LabChip (Agilent Technologies, Santa Clara, CA, USA).

*Amplification.* Total RNA was amplified using WT-Ovation One Direct RNA Amplification System V1.0 (NuGEN Technologies Inc., San Carlos, CA, USA), according to the standard protocol, from a starting amount of 500 pg. Amplified cDNA was consequently purified by MinElute Reaction Cleanup Kit (QIAGEN Inc., Valencia, CA, USA) according to the instructions described in the WT-Ovation One Direct protocol. RNA quality and quantity were assessed on an Agilent 2100 Bioanalyzer and RNA 6000 Pico LabChip. 

*Labeling*. After purification, theamplified single-stranded cDNA was labeled with biotin according to the NuGEN Illumina Protocol. 

*Hybridization*. Illumina HumanWG-6 v3 Expression BeadChip (Illumina, San Diego, CA, USA) was used for the microarray analysis following the standard protocol. Biotin-labeled cDNA (1.5 *μ*g) was hybridized, washed, and scanned according to the manufacturer's instructions, with the exception that the hybridization temperature was reduced to 48°C to accommodate the altered hybridization kinetics of cDNA/DNA pairs relative to cRNA/DNA pairs. All subsequent analyses were done on biological replicates.

### 2.3. Data Analysis

The raw data (TIFF image files) were analysed using the BeadArray package [25] of the bioconductor within the R environment (R Development Core Team 2007). All hybridizations passed quality control. The data were background corrected and normalized with the probe level quantile method. The probes with intensity level lower than the 95 percentile of negative controls of the BeadChip in all samples were disregarded before detection of differential expression. Differential expression was performed with the Limma package [26] on intensities that were variance-stabilized by logarithmic transformation. Annotation provided by bioconductor was used (*illuminaHumanv3BeadID.db*) [27]. Only transcripts with a false discovery rate (FDR) <0.05 and fold change <0.5 or >2 were reported and used in the downstream analysis. To identify significantly perturbed pathways, we performed SPIA [28] analysis on KEGG pathways: genes with FDR <0.05 were considered to be differentially transcribed. The data were deposited in the ArrayExpress database under accession number E-MTAB-1516.

## 3. Results

### 3.1. Comparison of Serum Expression Profiles of HNSCC Patients and Healthy Individuals

**Figure 1:**
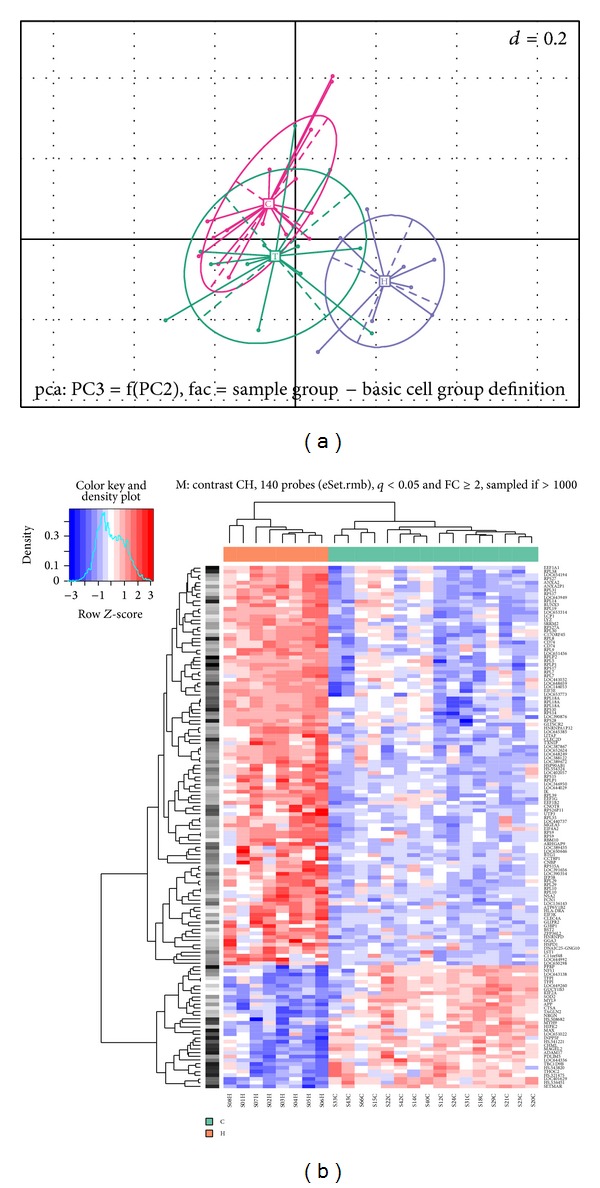
(a) Principal component analysis shows the global difference in presence of transcripts between serum specimens of H: healthy individuals, C: cancer patients before operation, and T: cancer patients one year after treatment. (b) Heat map shows the contrast in presence of transcripts between serum specimens of H (red line): healthy individuals and C (green line): cancer patients before treatment. Inside the heat map, blue color: downregulated genes and red color: upregulated genes.

The global gene expression pattern of the blood samples was analysed using principal component analysis (PCA). In this analysis all 16 HNSCC serum specimens were grouped together and were distinct from the normal specimens, showing that the pattern of gene expression was different in cancer patients and healthy individuals (Figures 1(a) and 1(b)).

1055 gene transcripts were significantly changed (*P* < 0.05, with at least a twofold change between normal and cancer serum specimens); see Supplementary Tables  S1 and S2 (see Tables S1 and S2 in the Supplementary Material available online at http://dx.doi.org/10.1155/2014/408683/). After correction to FDR (false discovery rate), we obtained 159 gene transcripts that were significantly changed (*P* value <0.05). Among these genes, we found the following groups as the most interesting: genes involved in the p53 signalling pathway (p53, p21, cyclinD, MDM2, CASP3, and MAX) and genes of the B-cell lymphoma (Bcl-2) family of proteins (Bcl-2, Bcl-XL, Bcl2L1, Mcl1, and BclAF1).

#### 3.1.1. Deregulated Myc Expression in HNSCC Patients

We found deregulated Myc expression (MAX: Myc associated factor X, *P* = 0.0099, logFC = 1.77 in the cancer-control group and *P* = 0.0055, logFC = 2 in the treated-control group) and upregulation of proapoptotic transmembrane protein Bim in different isoforms (TMBIM4 (transmembrane BAX inhibitor motif containing 4), *P* = 0.019, logFC = 1.78, and TMBIM1, *P* = 0.024, logFC = 1.69 in the cancer-control group and TMBIM4, *P* = 0.078, logFC = 1.36 and TMBIM1, *P* = 0.038, logFC = 1.61 in the treated-control group).

#### 3.1.2. Differences in Gene Expression in HNSCC Patient Serum before and 1 Year after Treatment

We compared 12 HNSCC serum samples from the same donors before and 1 year after treatment. The global gene expression analysis showed 246 changed genes (*P* < 0.05, with at least a twofold change). However, there were no significantly changed genes after FDR correction, demonstrating that the changes were less than those between cancer and normal serum samples.

#### 3.1.3. Signalling Pathway Analysis

Genes that were differentially expressed (*P* < 0.05) were analysed using signalling pathway impact analysis (SPIA) of the KEGG pathways. This combines the evidence obtained from the classical enrichment analysis with a novel type of evidence, which measures the actual perturbation on a given pathway under a given condition. In our dataset we were able to identify the pathways that were differentially activated or inhibited (FDR < 0.05) in serum of cancer patients and healthy individuals. The pathways that were significantly activated in our dataset were connected with antigen processing and presentation, focal adhesion, viral carcinogenesis, regulation of actin cytoskeleton, and chemokine signalling pathway or were involved in several viral infections (e.g., herpes simplex, influenza A, human T-lymphotrophic virus-I, and viral myocarditis). By contrast, the significantly inhibited pathways involved RNA transport, leukocyte transendothelial migration, natural-killer-cell-mediated cytotoxicity, and pathways connected with some neurodegenerative or autoimmune diseases (e.g., Parkinson's disease, Huntington's disease, and rheumatoid arthritis) (Table 2).

These findings were further supported by gene set enrichment analysis (GSEA) on the KEGG pathways, which showed significant deregulation of related pathways and downregulation of pathways connected with overall metabolism, RNA transport and degradation, and similar neurodegenerative diseases (Supplementary Table  S3).

Using GSEA on GO (gene ontology) terms, we found that the following influenced the biological processes: translational processes; ribosomal biogenesis; viral transcription; negative regulation of DNA damage response—signal transduction by p53 class mediator; induction of apoptosis; and several integrin- or interferon-mediated pathways. In terms of cellular compartment we detected changes mainly in the cytosolic or nuclear compartment (Supplementary Tables  S4 and  S5).

## 4. Discussion

Carcinogenesis and tumour progression are complex and progressive processes that are associated with numerous genetic and epigenetic alterations that can be detected in plasma or serum. Although there is a long history of investigation of circulating mRNA as a potential biomarker, not many relevant studies have used whole-genome microarray profiling [29, 30]. The aim of many microarray experiments is to determine the global genetic alterations that distinguish cancer cells from their normal counterparts. In our previous studies we mainly focused on the global transcriptome of cancer tissues in comparison with normal epithelium and especially peritumoural tissue. We have shown that paracrine secretion of growth factors, such as insulin-like growth factor-2 (IGF-2) and bone morphogenetic protein-4 (BMP-4), can elucidate the biological activity of stromal fibroblasts to normal keratinocytes by markedly influencing their phenotype. The induced keratinocytes acquire the appearance of squamous cell carcinoma keratinocytes or keratinocytes of wounded skin [31–35].

In light of these experiments, we tried to focus on markers that could potentially be present in the serum of cancer patients. There are several theories about how CNAs are released into the bloodstream. CNAs enter the bloodstream after apoptosis of nucleated cells or after tumour necrosis or are actively released into the circulation by tumour cells [36–38]. We need to realize that the changes in the different transcripts in the serum arise from a heterogeneous cell population—tumour cells, nucleated cells such as lymphocytes or monocytes, as well as a small number of thrombocytes. That is why we can expect changes in genes connected with cell death and tumour suppression, proliferation, and differentiation. There have been several studies using whole-genome microarray profiling to detect differences in serum gene expression in different types of solid tumours [39–42] as well as haematopoietic malignancies [43–45]. In our present study we adopted a similar design using whole-genome microarray profiling. What was particularly interesting was the combination of three different groups of samples, from patients before and after treatment as well as from healthy individuals.

As in numerous previous studies, we found that the apoptotic pathway was altered in the patients. Apoptosis is a major barrier to oncogenesis [46, 47] and is triggered by several factors that act through two major pathways: extrinsic and intrinsic pathways [48, 49]. In vertebrates most apoptosis proceeds through the intrinsic pathway. A regulator of this process is the Bcl-2 family of proteins [50]. The family comprises both antiapoptotic or prosurvival members (which can be divided into two subclasses: Bcl-2, Bcl-XL, and Bcl-w and Mcl-1 and A1) and proapoptotic members (the BAX subfamily that includes BAX, BAK, and BOK and the BH3-only subfamily that includes BID, BIM, BAD, BIK, BMF, PUMA, NOXA, and HRK) [51, 52]. The balance between these proteins determines whether a cell commits apoptosis, and the main regulator of these processes is p53. The identification of a myriad of proapoptotic p53 targets that bind and inhibit antiapoptotic Bcl-2 family members suggests that it is only through the combined transcriptional activation of numerous proapoptotic targets that p53 exerts its full apoptotic capability. Similarly, a combination of p53-dependent and -independent signals establishes a total apoptotic burden in a cell that stands in opposition to the prosurvival function of Bcl-2. In our study we revealed significant difference in presence of p53 transcripts in serum of cancer-control group gene expression of p53 in cancer-control group (*P* = 0.00031; logFC = −2.24) and in treated-control group (*P* = 0.019; logFC = −1.79), respectively. In contrast, we did not find a significant difference in expression in the cancer-treated group. As far as Bcl-2 family proteins are concerned, we detected a significant difference in expression of Bcl-2 and Bcl-XL transcripts in the treated-control group (Bcl-2, *P* = 0.004, logFC = −1.21; Bcl-XL, *P* = 0.006, logFC = 1.67). Overexpression of Bcl-XL in the treated group was evident and was 3.5 times higher than in the control group. In contrast, we did not detect a significant difference in expression of Bcl-2 family proteins in the cancer-control group (Bcl-2, *P* = 0.085; Bcl-XL, *P* = 0.14). In some studies overexpression of the antiapoptotic proteins Bcl-2 and Bcl-XL is associated with chemotherapy and radiation resistance [53, 54], and the combination of p53 status and Bcl-XL is associated with cisplatin resistance in HNSCC cells* in vitro* [55, 56]. The combination of low p53 and high Bcl-XL expression is associated with poor overall survival and disease specific survival [55, 57]. The negligible effect of HNSCC treatment on the activity of the above-mentioned genes, based on the level of cRNA, can be interpreted according to our recent results demonstrating differentiation-dependent expression of the endogenous lectin galectin-9 in normal squamous cell epithelium and in cancer [58]. Although normal squamous epithelium from noncancer patients demonstrated strictly basal cell expression, the malignant epithelium of tumours and a significant amount of histologically normal epithelium from HNSCC patients were devoid of expression of this lectin. This indicates some abnormality of histologically normal epithelial layer. These data suggest that the normal epithelium is damaged at the molecular level but to a lesser extent and complexity than is necessary for tumour formation. It harmonizes with hypothesis about field cancerisation [59].

**Figure 2:**
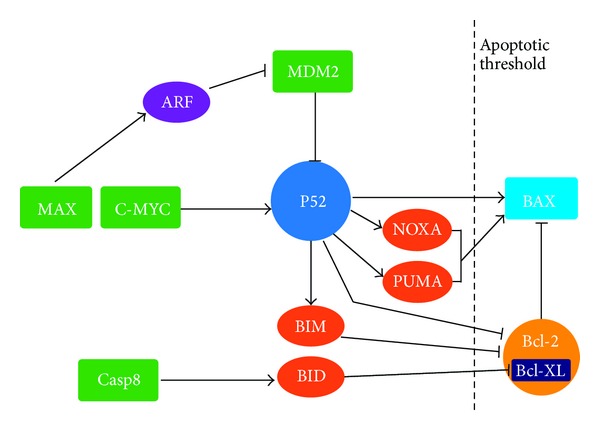
Role of p53 and other proteins (BH3 family, Bcl-2 family) in the apoptotic pathway.

Included in the apoptotic load are a number of BH3-only proteins that show no obvious regulation by p53 yet antagonise Bcl-2 function in response to specific cellular stresses (Figure 2). First, the BH3-only proteins Bim, Bad, and Hrk are induced by cytokine deprivation in a p53-independent manner [60], yet these proteins may synergize with p53-induced pathways to overcome the antiapoptotic threshold set by Bcl-2 and promote cell death. Second, deregulated Myc expression promotes p53-dependent apoptosis [61], but it also promotes the p53-independent activation of the proapoptotic BH3-only proteins Bim and Bax [62, 63]. Thus, p53-dependent and -independent signals act in parallel to promote cell death and suppress tumourigenesis. The combined strength of these signals is required to overwhelm the antiapoptotic Bcl-2 family members such that inactivation of any one of several prodeath effectors can drop the system below its apoptotic firing threshold and allow unabated proliferation. Myc is an important factor which regulates the expression of cellular targets involved in a wide range of diverse cellular functions, including cell growth, proliferation, loss of cell-cell contact, loss of differentiation, and angiogenesis. In our study we showed deregulated Myc expression in serum of HNSCC patients. Activation of Myc has been shown to cause cell growth, loss of differentiation, and cell cycle entry in suprabasal keratinocytes* in vivo* [64].

Our hypothesis is that apoptosis is altered in cancer patients due to resistance of Bcl-XL to p53-independent stimulation by Myc (MAX), potentially Bax, or other BH3-only proteins. The mechanisms of action of Bcl-2 and Bcl-XL are complex, with many postulated interactions with other proteins, and the role of any single interaction in the final phenotype at cellular level remains ill-defined. In some studies Bcl-XL has been ~10 times more active than Bcl-2 in repressing apoptosis in breast cancer cell lines [65]. When examined in the same cellular context, Bcl-2 and Bcl-XL differ substantially in the potency with which they inhibit apoptosis, mediated in part by differences in the inhibition of specific subcellular pathways.

Concerning the global difference in presence of transcripts in same patients before and one year after treatment, the principal component analysis (PCA) showed positive shift in treated patients in higher presence of transcripts corresponding more with the population of healthy individuals (see Figure 1(a)). This time period was not sufficient to make definite conclusions because some residual disease was present or patients underwent radical oncological treatment and the organism is not balanced yet. Nevertheless, there was a positive shift in PCA, which demonstrated that this method could be beneficial in the control of tumour recurrence.

The convergence of p53 on various aspects of Bcl-2 biology highlights the crucial role of this interaction in tumour suppression and drug response. Thus, promoting the p53-Bcl-2 interaction seemingly provides an ideal strategy for anticancer therapy. The relevance of specific p53-induced effector proteins and antiapoptotic Bcl-2 family members may vary in distinct tumourigenic contexts; therefore, understanding the precise apoptotic pathways abrogated in specific malignancies will be essential for devising targeted proapoptotic therapy. This includes expanding our understanding of how parallel apoptotic pathways synergise with p53-Bcl-2 signalling to promote cell death. We assumed that in cells the essential difference between Bcl-2 and Bcl-XL involved regulation of expression, probably due to expression in different tissues or in the same tissue but at different times.

We suggest that microchip analysis of serum cRNA in patients with HNSCC should be suitable for refinement of early stage diagnosis of disease that could be important for development of new personalised strategies in diagnosis and treatment of tumours. Either analysis of serum specimens of patients one year after treatment shows promising results in the meaning of shift to the population of healthy individuals.

## Supplementary Material

Supplementary Table S1: List of deregulated genes in serum of cancer patients in comparison to serum of healthy individuals (*p* < 0.05, logFC ≥ 1).
Supplementary Table S2: List of deregulated genes in serum of treated patients in comparison to serum of healthy individuals (*p* < 0.05, logFC ≥ 1).
Supplementary Table S3: List of deregulated KEGG pathways in serum of cancer patients in comparison to healthy individuals ( Nsig number of significantly changed genes in the pathway; Npath number of genes in the pathway).
Supplementary Table S4: GSEA on GO terms - biological process of serum specimens of cancer patients in comparison to healthy individuals shows significant deregulation in translational processes, ribosomal biogenesis, viral transcription, negative regulation of DNA damage response - signal transduction by p53 class mediator, induction of apoptosis (showed only GO terms with *p*-value < 0.05).
Supplementary Table S5: GSEA on GO terms - cytological compartment of serum specimens of cancer patients in comparison to healthy individuals shows changes mainly in cytosolic or nuclear compartment (showed only GO terms with *p*-value < 0.05).

## Figures and Tables

**Table:** (a)

Specimen	Gender	Age of diagnosis	Tumour site	Grade	pT	pN	Stage
S12C, S12T	F	42	Tonsillar fossa	G2	T2	N3	IV
S14C, S14T	M	61	Larynx	G2	T3	N0	III
S15C, S15T	M	59	Tongue margin	G2	T3	N2	IV
S18C, S18T	M	66	Soft palate	G2	T1	N0	I
S20C, S20T	M	63	Soft palate	G2	T1	N2	IV
S21C	M	62	Tonsillar fossa	G2	T2	N0	II
S22C, S22T	M	65	Body of tongue	G2	T2	N2	IV
S23C, S23T	M	65	Palatine tonsil	G1	T2	N1	III
S24C	M	73	Palatine tonsil	G2	T2	N2	IV
S29C, S29T	F	61	Larynx	G3	T4	N0	IV
S31C, S31T	M	58	Palatine tonsil	G2	T3	N3	IV
S33C, S33T	M	69	Root of tongue	G2	T2	N2	IV
S40C	F	73	Retromolar trigone	G1	T2	N2	IV
S42C, S42T	M	76	Piriform recess	G2	T3	N0	III
S43C, S43T	M	70	Palatine tonsil	G2	T2	N2	IV
S66C	M	51	Body of tongue	G1	T1	N0	I

**Table:** (b)

Specimen	Gender	Age of diagnosis	Diagnosis
S01H	M	29	Negative
S02H	M	74	Cystitis sinus *maxillaris *
S03H	F	41	Hypacusis conductiva
S04H	F	25	Perforation myringitis
S05H	F	48	Perforation myringitis
S06H	F	59	Atherosclerosis
S07H	M	32	SAS
S08H	M	26	Cystitis colli lateralis

**Table 2:** SPIA comparison of cancer serum specimens and healthy individuals serum specimens shows significant upregulation of pathways involved in antigen processing and presentation, focal adhesion, viral carcinogenesis, regulation of actin cytoskeleton, and downregulation of pathways involved in RNA transport, leukocyte transendothelial migration, natural-killer-cell-mediated cytotoxicity, and pathways connected with some neurodegenerative or autoimmune diseases.

KEGG ID	KEGG path	*N*_sig_	*N*_path_	FDR	Status
hsa05169	Epstein-Barr virus infection	198	NA	1.31*e* − 10	Inhibited
hsa04612	Antigen processing and presentation	75	107	3.71*e* − 05	Activated
hsa05203	Viral carcinogenesis	205	NA	3.71*e* − 05	Activated
hsa04510	Focal adhesion	201	329	3.98*e* − 05	Activated
hsa04810	Regulation of actin cytoskeleton	212	322	3.98*e* − 05	Activated
hsa03013	RNA transport	151	202	5.64*e* − 05	Inhibited
hsa05168	Herpes simplex infection	184	NA	5.64*e* − 05	Activated
hsa05164	Influenza A	173	NA	0.00019	Activated
hsa05323	Rheumatoid arthritis	91	119	0.000218	Inhibited
hsa05140	Leishmaniasis	72	99	0.000218	Activated
hsa05012	Parkinson's disease	111	138	0.000367	Inhibited
hsa05166	HTLV-I infection	260	NA	0.000542	Activated
hsa05110	*Vibrio cholerae* infection	54	90	6.00*e* − 04	Activated
hsa05322	Systemic lupus erythematosus	131	137	0.00269	Activated
hsa04062	Chemokine signaling pathway	188	268	0.00287	Activated
hsa05130	Pathogenic *Escherichia coli* infection	54	88	0.00334	Activated
hsa04914	Progesterone-mediated oocyte maturation	86	152	0.00334	Inhibited
hsa05016	Huntington's disease	168	218	0.00381	Inhibited
hsa05134	Legionellosis	55	NA	0.00389	Inhibited
hsa04670	Leukocyte transendothelial migration	115	175	0.00484	Inhibited
hsa05416	Viral myocarditis	70	107	0.0118	Activated
hsa05131	Shigellosis	61	112	0.0133	Activated
hsa05152	Tuberculosis	179	NA	0.0133	Activated
hsa04141	Protein processing in endoplasmic reticulum	163	236	0.0172	Activated
hsa05100	Bacterial invasion of epithelial cells	70	123	0.0236	Activated
hsa04660	T cell receptor signaling pathway	108	169	0.0266	Activated
hsa05145	Toxoplasmosis	133	193	0.027	Inhibited
hsa05150	*Staphylococcus aureus* infection	55	62	0.027	Activated
hsa04540	Gap junction	89	130	0.027	Activated
hsa05032	Morphine addiction	92	NA	0.027	Activated
hsa04940	Type I diabetes mellitus	43	54	0.0303	Inhibited
hsa04380	Osteoclast differentiation	132	193	0.0346	Activated
hsa04650	Natural-killer-cell-mediated cytotoxicity	134	189	0.0346	Inhibited
hsa05120	Epithelial cell signaling in *Helicobacter pylori* infection	68	100	0.0361	Activated
hsa03018	RNA degradation	69	97	0.0475	Inhibited

*N*_sig_: number of significantly changed genes in the pathway; *N*_path_: number of genes in the pathway; FDR: false discovery rate.
