# Supplementary material for: Microarray Analysis of Serum mRNA in Patients with Head and Neck Squamous Cell Carcinoma at Whole-Genome Scale
Source: Biomed Res Int. 2014 Apr 23;2014:408683. doi: 10.1155/2014/408683 (PMC4017838; doi:10.1155/2014/408683)
Supplement: Supplementary file 1 — Supplementary Table S1: List of deregulated genes in serum of cancer patients in comparison to serum of healthy individuals (p < 0.05, logFC ≥ 1). Supplementary Table S2: List of deregulated genes in serum of treated patients in comparison to serum of healthy individuals (p < 0.05, logFC ≥ 1). Supplementary Table S3: List of deregulated KEGG pathways in serum of cancer patients in comparison to healthy individuals ( Nsig number of significantly changed genes in the pathway; Npath number of genes in the pathway). Supplementary Table S4: GSEA on GO terms - biological process of serum specimens of cancer patients in comparison to healthy individuals shows significant deregulation in translational processes, ribosomal biogenesis, viral transcription, negative regulation of DNA damage response - signal transduction by p53 class mediator, induction of apoptosis (showed only GO terms with p-value < 0.05). Supplementary Table S5: GSEA on GO terms - cytological compartment of serum specimens of cancer patients in comparison to healthy individuals shows changes mainly in cytosolic or nuclear compartment (showed only GO terms with p-value < 0.05). [file 408683.f1.pdf]

Supporting information

Additional Supporting Information may be found in the online version of this article:

Supplementary Table S1: List of deregulated genes in serum of cancer patients in comparision to serum of healthy individuals ($p < 0.05$, logFC ≥ 1).

ENTREZ Gene ID	Symbol	Gene Name	logFC
8407	TAGLN2	transgelin 2	3,78
7035	TFPI	tissue factor pathway inhibitor (lipoprotein-associated coagulation inhibitor)	3,53
28996	HIPK2	homeodomain interacting protein kinase 2	3,49
3690	ITGB3	integrin, beta 3 (platelet glycoprotein IIIa, antigen CD61)	3,48
7035	TFPI	tissue factor pathway inhibitor (lipoprotein-associated coagulation inhibitor)	3,45
4900	NRGN	neurogranin (protein kinase C substrate, RC3)	3,32
10398	MYL9	myosin, light chain 9, regulatory	3,22
3796	KIF2A	kinesin heavy chain member 2A	3,14
5476	CTSA	cathepsin A	3,08
6648	SOD2	superoxide dismutase 2, mitochondrial	3,07
2982	GUCY1A3	guanylate cyclase 1, soluble, alpha 3	3,07
8459	TPST2	tyrosylprotein sulfotransferase 2	3,05
2983	GUCY1B3	guanylate cyclase 1, soluble, beta 3	3,04
145781	GCOM1	GRINL1A complex locus	3,02
10611	PDLIM5	PDZ and LIM domain 5	2,87
5567	PRKACB	protein kinase, cAMP-dependent, catalytic, beta	2,85
25907	TMEM158	transmembrane protein 158 (gene/pseudogene)	2,84
8848	TSC22D1	TSC22 domain family, member 1	2,83

351	APP	amyloid beta (A4) precursor protein	2,82
9240	PNMA1	paraneoplastic antigen MA1	2,78
400073	C12orf76	chromosome 12 open reading frame 76	2,78
649260	ILMN_35781	PREDICTED: Homo sapiens similar to LIM and senescent cell antigen-like domains 1 (LOC649260), mRNA.	2,77
8407	TAGLN2	transgelin 2	2,74
2162	F13A1	coagulation factor XIII, A1 polypeptide	2,74
9948	WDR1	WD repeat domain 1	2,73
81027	TUBB1	tubulin, beta 1	2,73
9848	MFAP3L	microfibrillar-associated protein 3-like	2,73
51257	2.III	membrane-associated ring finger (C3HC4) 2	2,73
25994	HIGD1A	HIG1 hypoxia inducible domain family, member 1A	2,71
8992	ATP6V0E1	ATPase, H+ transporting, lysosomal 9kDa, V0 subunit e1	2,7
8545	CGGBP1	CGG triplet repeat binding protein 1	2,68
2017	CTTN	cortactin	2,67
5413	ILMN_139223	Homo sapiens septin 5 (SEPT5), mRNA.	2,64
196527	ANO6	anoctamin 6	2,62
7485	WRB	tryptophan rich basic protein	2,61
222166	C7orf41	chromosome 7 open reading frame 41	2,59
10079	ATP9A	ATPase, class II, type 9A	2,59
2040	STOM	stomatin	2,58
58497	PRUNE	prune homolog (Drosophila)	2,57
83699	ILMN_9801	Homo sapiens SH3 domain binding glutamic acid-rich protein like 2 (SH3BGRL2), mRNA.	2,56
27102	EIF2AK1	eukaryotic translation initiation factor 2-alpha kinase 1	2,56
401629	ILMN_14074	Homo sapiens hypothetical LOC401629 (LOC401629), non-coding RNA.	2,54
2791	GNG11	guanine nucleotide binding protein (G protein), gamma 11	2,54
147199	SCGB1C1	secretoglobin, family 1C, member 1	2,51
1998	ELF2	E74-like factor 2 (ets domain transcription factor)	2,5
89872	AQP10	aquaporin 10	2,49
4673	NAP1L1	nucleosome assembly protein 1-like 1	2,48
4638	MYLK	myosin light chain kinase	2,48
3017	HIST1H2BD	histone cluster 1, H2bd	2,47
643319	ILMN_41137	PREDICTED: Homo sapiens similar to Transgelin-2 (LOC643319), mRNA.	2,44
9910	RABGAP1L	RAB GTPase activating protein 1-like	2,43

3987	LIMS1	LIM and senescent cell antigen-like domains 1	2,43
444	ASPH	aspartate beta-hydroxylase	2,43
54498	SMOX	spermine oxidase	2,42
8349	ILMN_28293	Homo sapiens histone cluster 2, H2be (HIST2H2BE), mRNA.	2,42
127018	LYPLAL1	lysophospholipase-like 1	2,4
643138	ILMN_39430	PREDICTED: Homo sapiens similar to Guanine nucleotide-binding protein G(s), alpha subunit (Adenylate cyclase-stimulating G alpha protein) (G-alpha-8) (LOC643138), mRNA.	2,4
2108	ETFA	electron-transfer-flavoprotein, alpha polypeptide	2,39
54469	ZFAND6	zinc finger, AN1-type domain 6	2,38
10611	PDLIM5	PDZ and LIM domain 5	2,38
401232	ILMN_11265	Homo sapiens hypothetical protein DKFZp686I15217 (DKFZp686I15217), mRNA.	2,38
87	ACTN1	actinin, alpha 1	2,38
4052	LTBP1	latent transforming growth factor beta binding protein 1	2,35
79053	ALG8	asparagine-linked glycosylation 8, alpha-1,3-glucosyltransferase homolog (S. cerevisiae)	2,35
10653	SPINT2	serine peptidase inhibitor, Kunitz type, 2	2,33
7414	VCL	vinculin	2,31
5889	RAD51C	RAD51 homolog C (S. cerevisiae)	2,31
10826	C5orf4	chromosome 5 open reading frame 4	2,3
6648	SOD2	superoxide dismutase 2, mitochondrial	2,29
153768	PRELID2	PRELI domain containing 2	2,29
9054	NFS1	NFS1 nitrogen fixation 1 homolog (S. cerevisiae)	2,29
644422	ILMN_34341	PREDICTED: Homo sapiens misc_RNA (LOC644422), miscRNA.	2,29
51266	CLEC1B	C-type lectin domain family 1, member B	2,29
10867	TSPAN9	tetraspanin 9	2,28
3017	HIST1H2BD	histone cluster 1, H2bd	2,28
25994	HIGD1A	HIG1 hypoxia inducible domain family, member 1A	2,28
7057	THBS1	thrombospondin 1	2,27
5795	PTPRJ	protein tyrosine phosphatase, receptor type, J	2,27
441081	ILMN_36410	PREDICTED: Homo sapiens similar to nuclear pore membrane protein 121 (LOC441081), mRNA.	2,27
3987	LIMS1	LIM and senescent cell antigen-like domains 1	2,27
1069	CETN2	centrin, EF-hand protein, 2	2,27
63933	CCDC90A	coiled-coil domain containing 90A	2,27
830	CAPZA2	capping protein (actin filament) muscle Z-line, alpha 2	2,27

5154	PDGFA	platelet-derived growth factor alpha polypeptide	2,26
9848	MFAP3L	microfibrillar-associated protein 3-like	2,24
442124	ILMN_37787	PREDICTED: Homo sapiens hypothetical LOC442124 (LOC442124), mRNA.	2,24
10217	CTDSPL	CTD (carboxy-terminal domain, RNA polymerase II, polypeptide A) small phosphatase-like	2,23
3934	LCN2	lipocalin 2	2,19
340205	TREML1	triggering receptor expressed on myeloid cells-like 1	2,18
23429	RYBP	RING1 and YY1 binding protein	2,18
80212	CCDC92	coiled-coil domain containing 92	2,18
351	APP	amyloid beta (A4) precursor protein	2,17
9402	GRAP2	GRB2-related adaptor protein 2	2,15
84859	LRCH3	leucine-rich repeats and calponin homology (CH) domain containing 3	2,14
222658	KCTD20	potassium channel tetramerisation domain containing 20	2,14
	ILMN_103084	BX105743 NCI_CGAP_GC6 Homo sapiens cDNA clone IMAGp998H135716, mRNA sequence	2,14
139105	ILMN_3923	Homo sapiens chromosome X open reading frame 20 (CXorf20), mRNA.	2,14
3688	ITGB1	integrin, beta 1 (fibronectin receptor, beta polypeptide, antigen CD29 includes MDF2, MSK12)	2,13
10666	CD226	CD226 molecule	2,13
51109	RDH11	retinol dehydrogenase 11 (all-trans/9-cis/11-cis)	2,12
	ILMN_105608	AV702854 ADB Homo sapiens cDNA clone ADBCYG05 5, mRNA sequence	2,12
55755	CDK5RAP2	CDK5 regulatory subunit associated protein 2	2,12
54516	ILMN_182901	Homo sapiens mitochondrial translational release factor 1-like (MTRF1L), mRNA.	2,11
645128	ILMN_40515	PREDICTED: Homo sapiens similar to DEAD/H (Asp-Glu-Ala-Asp/His) box polypeptide 11 isoform 1 (LOC645128), mRNA.	2,1
23558	WBP2	WW domain binding protein 2	2,09
30061	SLC40A1	solute carrier family 40 (iron-regulated transporter), member 1	2,09
5742	ILMN_3001	Homo sapiens prostaglandin-endoperoxide synthase 1 (prostaglandin G/H synthase and cyclooxygenase) (PTGS1), transcript variant 2, mRNA.	2,09
1026	ILMN_5895	Homo sapiens cyclin-dependent kinase inhibitor 1A (p21, Cip1) (CDKN1A), transcript variant 1, mRNA.	2,09
732424	ILMN_119261	PREDICTED: Homo sapiens hypothetical protein LOC732424 (LOC732424), mRNA.	2,07
391475	DYTN	dystrotelin	2,07
4267	CD99	CD99 molecule	2,07
2040	STOM	stomatin	2,05
64407	RGS18	regulator of G-protein signaling 18	2,05
55132	LARP1B	La ribonucleoprotein domain family, member 1B	2,05

2280	FKBP1A	FK506 binding protein 1A, 12kDa	2,05
80739	C6orf25	chromosome 6 open reading frame 25	2,05
4627	MYH9	myosin, heavy chain 9, non-muscle	2,04
5028	P2RY1	purinergic receptor P2Y, G-protein coupled, 1	2,03
7456	WIPF1	WAS/WASL interacting protein family, member 1	2,02
649214	ILMN_33279	PREDICTED: Homo sapiens similar to High mobility group protein 1-like 10 (HMG-1L10), transcript variant 4 (LOC649214), mRNA.	2,02
	ILMN_97811	AV762101 MDS Homo sapiens cDNA clone MDSEOA03 5, mRNA sequence	2,02
643752	RAP1BL	RAP1B, member of RAS oncogene family pseudogene	2
644799	ILMN_43702	PREDICTED: Homo sapiens hypothetical protein LOC644799, transcript variant 1 (LOC644799), mRNA.	2
51706	CYB5R1	cytochrome b5 reductase 1	1,99
9556	C14orf2	chromosome 14 open reading frame 2	1,99
23531	MMD	monocyte to macrophage differentiation-associated	1,98
10938	EHD1	EH-domain containing 1	1,98
3013	HIST1H2AD	histone cluster 1, H2ad	1,97
6117	RPA1	replication protein A1, 70kDa	1,96
54741	ILMN_27032	Homo sapiens leptin receptor overlapping transcript (LEPROT), mRNA.	1,96
10049	DNAJB6	DnaJ (Hsp40) homolog, subfamily B, member 6	1,96
5196	ILMN_16715	Homo sapiens platelet factor 4 (chemokine (C-X-C motif) ligand 4) (PF4), mRNA.	1,95
	ILMN_88180	ir56a05.y1 HR85 islet Homo sapiens cDNA clone IMAGE:6549201 5, mRNA sequence	1,95
644162	ILMN_43225	PREDICTED: Homo sapiens similar to septin 7, transcript variant 4 (LOC644162), mRNA.	1,94
4638	MYLK	myosin light chain kinase	1,93
351	APP	amyloid beta (A4) precursor protein	1,93
5874	RAB27B	RAB27B, member RAS oncogene family	1,92
9588	PRDX6	peroxiredoxin 6	1,92
83658	DYNLRB1	dynein, light chain, roadblock-type 1	1,92
83700	JAM3	junctional adhesion molecule 3	1,91
6374	CXCL5	chemokine (C-X-C motif) ligand 5	1,91
5197	PF4V1	platelet factor 4 variant 1	1,9
10000	AKT3	v-akt murine thymoma viral oncogene homolog 3 (protein kinase B, gamma)	1,9
9445	ITM2B	integral membrane protein 2B	1,89
5087	PBX1	pre-B-cell leukemia homeobox 1	1,88
55353	LAPTM4B	lysosomal protein transmembrane 4 beta	1,88

2811	GP1BA	glycoprotein Ib (platelet), alpha polypeptide	1,88
653061	ILMN_40836	PREDICTED: Homo sapiens similar to golgi autoantigen, golgin subfamily a, 8A (LOC653061), mRNA.	1,87
	ILMN_93150	UI-H-EU0-azk-k-10-0-UI.s1 NCI_CGAP_Car1 Homo sapiens cDNA clone IMAGE: 5849985 3, mRNA sequence	1,87
2149	ILMN_25961	Homo sapiens coagulation factor II (thrombin) receptor (F2R), mRNA.	1,87
80790	ILMN_13851	Homo sapiens c-Maf-inducing protein (CMIP), transcript variant Tc-mip, mRNA.	1,87
5305	PIP4K2A	phosphatidylinositol-5-phosphate 4-kinase, type II, alpha	1,86
375033	ILMN_170351	Homo sapiens platelet endothelial aggregation receptor 1 (PEAR1), mRNA.	1,86
3688	ITGB1	integrin, beta 1 (fibronectin receptor, beta polypeptide, antigen CD29 includes MDF2, MSK12)	1,86
84771	ILMN_42231	PREDICTED: Homo sapiens hypothetical protein MGC13005 (MGC13005), mRNA.	1,84
644336	ILMN_44408	PREDICTED: Homo sapiens similar to SET domain and mariner transposase fusion gene (LOC644336), mRNA.	1,84
79647	AKIRIN1	akirin 1	1,84
3987	ILMN_139143	PREDICTED: Homo sapiens LIM and senescent cell antigen-like domains 1 (LIMS1), mRNA.	1,83
7414	VCL	vinculin	1,82
648057	ILMN_32589	PREDICTED: Homo sapiens similar to Guanine nucleotide-binding protein G(s), alpha subunit (Adenylate cyclase-stimulating G alpha protein) (G-alpha-8) (LOC648057), mRNA.	1,82
643451	ILMN_31270	PREDICTED: Homo sapiens hypothetical protein LOC643451 (LOC643451), mRNA.	1,81
25932	CLIC4	chloride intracellular channel 4	1,81
9628	RGS6	regulator of G-protein signaling 6	1,8
8440	NCK2	NCK adaptor protein 2	1,8
27257	LSM1	LSM1 homolog, U6 small nuclear RNA associated (S. cerevisiae)	1,8
83658	DYNLRB1	dynein, light chain, roadblock-type 1	1,8
30850	CDR2L	cerebellar degeneration-related protein 2-like	1,8
25893	TRIM58	tripartite motif containing 58	1,79
653635	ILMN_42161	PREDICTED: Homo sapiens similar to CXYorf1-related protein (LOC653635), mRNA.	1,79
8655	DYNLL1	dynein, light chain, LC8-type 1	1,79
401232	ILMN_11265	Homo sapiens hypothetical protein DKFZp686I15217 (DKFZp686I15217), mRNA.	1,79
51643	TMBIM4	transmembrane BAX inhibitor motif containing 4	1,78
326624	RAB37	RAB37, member RAS oncogene family	1,78
23299	BICD2	bicaudal D homolog 2 (Drosophila)	1,78
94121	SYTL4	synaptotagmin-like 4	1,77
91624	NEXN	nexilin (F actin binding protein)	1,77
4149	MAX	MYC associated factor X	1,77
79742	CXorf36	chromosome X open reading frame 36	1,77

643253	CCT6P1	chaperonin containing TCP1, subunit 6 (zeta) pseudogene 1	1,77
54498	SMOX	spermine oxidase	1,76
10169	SERF2	small EDRK-rich factor 2	1,76
5579	PRKCB	protein kinase C, beta	1,76
5552	SRGN	serglycin	1,75
644380	ILMN_36938	PREDICTED: Homo sapiens similar to High mobility group protein 1-like 10 (HMG-1L10), transcript variant 1 (LOC644380), mRNA.	1,75
148823	ILMN_22220	Homo sapiens chromosome 1 open reading frame 150 (C1orf150), mRNA.	1,74
54470	ILMN_6931	Homo sapiens armadillo repeat containing, X-linked 6 (ARMCX6), transcript variant 1, mRNA.	1,74
64795	RMND5A	required for meiotic nuclear division 5 homolog A (S. cerevisiae)	1,73
51251	NT5C3	5'-nucleotidase, cytosolic III	1,73
10398	MYL9	myosin, light chain 9, regulatory	1,73
7326	UBE2G1	ubiquitin-conjugating enzyme E2G 1	1,72
4627	MYH9	myosin, heavy chain 9, non-muscle	1,72
4149	MAX	MYC associated factor X	1,72
9764	KIAA0513	KIAA0513	1,72
2876	ILMN_10376	Homo sapiens glutathione peroxidase 1 (GPX1), transcript variant 2, mRNA.	1,72
83706	FERMT3	fermitin family member 3	1,72
55281	TMEM140	transmembrane protein 140	1,71
30061	SLC40A1	solute carrier family 40 (iron-regulated transporter), member 1	1,71
64805	P2RY12	purinergic receptor P2Y, G-protein coupled, 12	1,7
387820	ILMN_26714	PREDICTED: Homo sapiens similar to DnaJ (Hsp40) homolog, subfamily B, member 6 isoform a (LOC387820), mRNA.	1,7
3988	LIPA	lipase A, lysosomal acid, cholesterol esterase	1,7
283777	FAM169B	family with sequence similarity 169, member B	1,7
64114	TMBIM1	transmembrane BAX inhibitor motif containing 1	1,69
8436	SDPR	serum deprivation response	1,67
642707	ILMN_43996	PREDICTED: Homo sapiens similar to Hornerin (LOC642707), mRNA.	1,67
5867	ILMN_14453	Homo sapiens RAB4A, member RAS oncogene family (RAB4A), mRNA.	1,66
5816	PVALB	parvalbumin	1,66
	ILMN_125202	IL2-NT0102-160600-105-C03 NT0102 Homo sapiens cDNA, mRNA sequence	1,66
26052	DNM3	dynamin 3	1,66
84913	ATOH8	atonal homolog 8 (Drosophila)	1,66
56897	WRNIP1	Werner helicase interacting protein 1	1,65

651361	ILMN_35263	PREDICTED: Homo sapiens similar to S-phase kinase-associated protein 1A (Cyclin A/CDK2-associated protein p19) (p19A) (p19skp1) (LOC651361), mRNA.	1,65
10477	UBE2E3	ubiquitin-conjugating enzyme E2E 3	1,64
8655	DYNLL1	dynein, light chain, LC8-type 1	1,64
136319	MTPN	myotrophin	1,63
8417	STX7	syntaxin 7	1,62
4218	RAB8A	RAB8A, member RAS oncogene family	1,61
3580	ILMN_139000	PREDICTED: Homo sapiens interleukin 8 receptor, beta pseudogene (IL8RBP), misc RNA.	1,61
55294	FBXW7	F-box and WD repeat domain containing 7	1,61
6352	CCL5	chemokine (C-C motif) ligand 5	1,61
27018	NGFRAP1	nerve growth factor receptor (TNFRSF16) associated protein 1	1,6
4354	MPP1	membrane protein, palmitoylated 1, 55kDa	1,6
3693	ITGB5	integrin, beta 5	1,6
84548	TMEM185A	transmembrane protein 185A	1,59
80230	RUFY1	RUN and FYVE domain containing 1	1,59
55796	MBNL3	muscleblind-like 3 (Drosophila)	1,59
647958	ILMN_31883	PREDICTED: Homo sapiens similar to SET domain and mariner transposase fusion gene (LOC647958), mRNA.	1,59
3146	HMGB1	high mobility group box 1	1,59
9445	ITM2B	integral membrane protein 2B	1,58
	ILMN_86463	RST15665 Athersys RAGE Library Homo sapiens cDNA, mRNA sequence	1,58
64423	INF2	inverted formin, FH2 and WH2 domain containing	1,58
51646	YPEL5	yippee-like 5 (Drosophila)	1,57
51251	NT5C3	5'-nucleotidase, cytosolic III	1,57
64747	MFSD1	major facilitator superfamily domain containing 1	1,57
2664	ILMN_13492	Homo sapiens GDP dissociation inhibitor 1 (GDI1), mRNA.	1,57
3953	LEPR	leptin receptor	1,56
1523	CUX1	cut-like homeobox 1	1,56
56344	CABP5	calcium binding protein 5	1,56
5494	PPM1A	protein phosphatase, Mg2+/Mn2+ dependent, 1A	1,55
651022	ILMN_31551	PREDICTED: Homo sapiens hypothetical protein LOC651022 (LOC651022), mRNA.	1,55
8328	GFI1B	growth factor independent 1B transcription repressor	1,55
9465	AKAP7	A kinase (PRKA) anchor protein 7	1,55
5627	PROS1	protein S (alpha)	1,54

5577	PRKAR2B	protein kinase, cAMP-dependent, regulatory, type II, beta	1,54
650263	ILMN_46692	PREDICTED: Homo sapiens hypothetical protein LOC650263 (LOC650263), mRNA.	1,54
8339	HIST1H2BG	histone cluster 1, H2bg	1,54
1727	CYB5R3	cytochrome b5 reductase 3	1,54
6397	SEC14L1	SEC14-like 1 (S. cerevisiae)	1,53
10158	PDZK1IP1	PDZK1 interacting protein 1	1,53
11315	PARK7	parkinson protein 7	1,53
3987	LIMS1	LIM and senescent cell antigen-like domains 1	1,53
7504	XK	X-linked Kx blood group (McLeod syndrome)	1,52
8654	PDE5A	phosphodiesterase 5A, cGMP-specific	1,52
123803	NTAN1	N-terminal asparagine amidase	1,52
440917	LOC440917	tyrosine 3-monooxygenase/tryptophan 5-monooxygenase activation protein, epsilon polypeptide pseudogene	1,52
27292	DIMT1	DIM1 dimethyladenosine transferase 1 homolog (S. cerevisiae)	1,52
400804	ILMN_29977	Homo sapiens chromosome 1 open reading frame 140 (C1orf140), mRNA.	1,52
7533	YWHAH	tyrosine 3-monooxygenase/tryptophan 5-monooxygenase activation protein, eta polypeptide	1,51
5782	PTPN12	protein tyrosine phosphatase, non-receptor type 12	1,51
389787	LOC389787	tumor protein, translationally-controlled 1 pseudogene	1,51
10357	HMGB1P1	high mobility group box 1 pseudogene 1	1,51
395	ARHGAP6	Rho GTPase activating protein 6	1,51
84519	ACRBP	acrosin binding protein	1,51
340348	TSPAN33	tetraspanin 33	1,5
6648	ILMN_19880	Homo sapiens superoxide dismutase 2, mitochondrial (SOD2), nuclear gene encoding mitochondrial protein, transcript variant 1, mRNA.	1,5
	ILMN_81934	UI-H-DF1-auk-p-10-0-UI.s1 NCI_CGAP_DF1 Homo sapiens cDNA clone IMAGE:5870841 3, mRNA sequence	1,5
8345	HIST1H2BH	histone cluster 1, H2bh	1,5
7076	TIMP1	TIMP metallopeptidase inhibitor 1	1,49
	ILMN_111407	xb22e11.x1 NCI_CGAP_Kid13 Homo sapiens cDNA clone IMAGE:2577068 3, mRNA sequence	1,49
9455	HOMER2	homer homolog 2 (Drosophila)	1,49
3081	HGD	homogentisate 1,2-dioxygenase	1,49
55845	BRK1	BRICK1, SCAR/WAVE actin-nucleating complex subunit	1,49
6748	SSR4	signal sequence receptor, delta	1,48
5911	RAP2A	RAP2A, member of RAS oncogene family	1,48
3572	IL6ST	interleukin 6 signal transducer (gp130, oncostatin M receptor)	1,48

56911	C21orf7	chromosome 21 open reading frame 7	1,48
7531	YWHAE	tyrosine 3-monooxygenase/tryptophan 5-monooxygenase activation protein, epsilon polypeptide	1,47
	ILMN_72868	TCAAP1D4752 Pediatric acute myelogenous leukemia cell (FAB M1) Baylor-HGSC project=TCAA Homo sapiens cDNA clone TCAAP4752, mRNA sequence	1,47
2280	FKBP1A	FK506 binding protein 1A, 12kDa	1,47
11235	PDCD10	programmed cell death 10	1,46
4154	MBNL1	muscleblind-like (Drosophila)	1,46
257019	FRMD3	FERM domain containing 3	1,46
6500	SKP1	S-phase kinase-associated protein 1	1,45
10929	SRSF8	serine/arginine-rich splicing factor 8	1,45
58508	MLL3	myeloid/lymphoid or mixed-lineage leukemia 3	1,45
	ILMN_110914	BX429982 Homo sapiens FETAL LIVER Homo sapiens cDNA clone CS0DM002YI05 5-PRIME, mRNA sequence	1,45
112495	GTF3C6	general transcription factor IIIC, polypeptide 6, alpha 35kDa	1,45
488	ILMN_19965	Homo sapiens ATPase, Ca++ transporting, cardiac muscle, slow twitch 2 (ATP2A2), transcript variant 1, mRNA.	1,45
54470	ARMCX6	armadillo repeat containing, X-linked 6	1,45
7168	TPM1	tropomyosin 1 (alpha)	1,44
6419	ILMN_17510	Homo sapiens SET domain and mariner transposase fusion gene (SETMAR), mRNA.	1,44
8780	RIOK3	RIO kinase 3 (yeast)	1,44
8766	RAB11A	RAB11A, member RAS oncogene family	1,44
2026	ENO2	enolase 2 (gamma, neuronal)	1,44
22885	ABLIM3	actin binding LIM protein family, member 3	1,44
6399	TRAPPC2	trafficking protein particle complex 2	1,43
8714	ILMN_8120	Homo sapiens ATP-binding cassette, sub-family C (CFTR/MRP), member 3 (ABCC3), mRNA.	1,43
8848	TSC22D1	TSC22 domain family, member 1	1,42
8848	TSC22D1	TSC22 domain family, member 1	1,42
6117	RPA1	replication protein A1, 70kDa	1,42
326624	ILMN_10097	Homo sapiens RAB37, member RAS oncogene family (RAB37), transcript variant 1, mRNA.	1,42
326624	RAB37	RAB37, member RAS oncogene family	1,42
652271	ILMN_37313	PREDICTED: Homo sapiens similar to N-acylsphingosine amidohydrolase 2 (LOC652271), mRNA.	1,42
81689	ISCA1	iron-sulfur cluster assembly 1 homolog (S. cerevisiae)	1,42
9923	ZBTB40	zinc finger and BTB domain containing 40	1,41
5552	SRGN	serglycin	1,41
113230	ILMN_33629	PREDICTED: Homo sapiens hypothetical protein LOC113230 (LOC113230), mRNA.	1,41

3688	ITGB1	integrin, beta 1 (fibronectin receptor, beta polypeptide, antigen CD29 includes MDF2, MSK12)	1,41
	ILMN_96170	ny42b04.s1 NCI_CGAP_Pr12 Homo sapiens cDNA clone IMAGE:1274383, mRNA sequence	1,41
6374	CXCL5	chemokine (C-X-C motif) ligand 5	1,41
80025	PANK2	pantothenate kinase 2	1,4
2768	GNA12	guanine nucleotide binding protein (G protein) alpha 12	1,4
10152	ABI2	abl-interactor 2	1,4
10857	PGRMC1	progesterone receptor membrane component 1	1,39
1200	ILMN_19883	Homo sapiens tripeptidyl peptidase I (TPP1), mRNA.	1,38
55529	TMEM55A	transmembrane protein 55A	1,38
6757	ILMN_163873	Homo sapiens synovial sarcoma, X breakpoint 2 (SSX2), transcript variant 1, mRNA.	1,38
8773	SNAP23	synaptosomal-associated protein, 23kDa	1,38
8654	PDE5A	phosphodiesterase 5A, cGMP-specific	1,38
	ILMN_128554	CR748447 Soares_testis_NHT Homo sapiens cDNA clone IMAGp971I1175 ; IMAGE:1738834 5, mRNA sequence	1,38
	ILMN_79152	xj44f04.x1 Soares_NFL_T_GBC_S1 Homo sapiens cDNA clone IMAGE:2660095 3, mRNA sequence	1,38
4953	ODC1	ornithine decarboxylase 1	1,37
4130	MAP1A	microtubule-associated protein 1A	1,37
653226	ILMN_39856	PREDICTED: Homo sapiens similar to Signal recognition particle 9 kDa protein (SRP9) (LOC653226), mRNA.	1,37
29094	LGALSL	lectin, galactoside-binding-like	1,37
2766	GMPR	guanosine monophosphate reductase	1,37
50848	F11R	F11 receptor	1,36
649395	LOC649395	tyrosine 3-monooxygenase/tryptophan 5-monooxygenase activation protein, epsilon polypeptide pseudogene	1,35
6714	SRC	v-src sarcoma (Schmidt-Ruppin A-2) viral oncogene homolog (avian)	1,35
8773	SNAP23	synaptosomal-associated protein, 23kDa	1,35
56950	SMYD2	SET and MYND domain containing 2	1,35
6403	SELP	selectin P (granule membrane protein 140kDa, antigen CD62)	1,35
2778	GNAS	GNAS complex locus	1,35
1729	DIAPH1	diaphanous homolog 1 (Drosophila)	1,35
8804	CREG1	cellular repressor of E1A-stimulated genes 1	1,35
760	CA2	carbonic anhydrase II	1,35
444	ASPH	aspartate beta-hydroxylase	1,35
7168	TPM1	tropomyosin 1 (alpha)	1,34
26994	RNF11	ring finger protein 11	1,34
4149	MAX	MYC associated factor X	1,34

2867	FFAR2	free fatty acid receptor 2	1,34
83658	DYNLRB1	dynein, light chain, roadblock-type 1	1,34
29797	ILMN_28495	Homo sapiens hypothetical protein DKFZp434K191 (DKFZp434K191), mRNA.	1,34
6881	TAF10	TAF10 RNA polymerase II, TATA box binding protein (TBP)-associated factor, 30kDa	1,33
8935	SKAP2	src kinase associated phosphoprotein 2	1,33
387590	ILMN_12454	Homo sapiens TPTE pseudogene (psiTPTE22), non-coding RNA.	1,33
55898	UNC45A	unc-45 homolog A (C. elegans)	1,32
9978	RBX1	ring-box 1, E3 ubiquitin protein ligase	1,32
9124	PDLIM1	PDZ and LIM domain 1	1,32
400073	C12orf76	chromosome 12 open reading frame 76	1,32
6006	RHCE	Rh blood group, CcEe antigens	1,31
54551	MAGEL2	MAGE-like 2	1,31
643713	ILMN_40960	PREDICTED: Homo sapiens hypothetical protein LOC643713 (LOC643713), mRNA.	1,31
977	ILMN_44841	Homo sapiens CD151 molecule (Raph blood group) (CD151), transcript variant 2, mRNA.	1,31
6868	ILMN_165100	Homo sapiens ADAM metallopeptidase domain 17 (ADAM17), mRNA.	1,31
6774	STAT3	signal transducer and activator of transcription 3 (acute-phase response factor)	1,3
644436	ILMN_42336	PREDICTED: Homo sapiens similar to Tubulin alpha-4 chain (Alpha-tubulin 4) (Alpha-tubulin isotype M-alpha-4) (LOC644436), mRNA.	1,3
	ILMN_111904	UI-CF-DU1-aay-b-22-18-UI.s18 UI-CF-DU1 Homo sapiens cDNA clone UI-CF-DU1-aay-b-22-18-UI 3, mRNA sequence	1,3
408	ARRB1	arrestin, beta 1	1,3
57187	THOC2	THO complex 2	1,29
	ILMN_107341	PM3-LT0032-090100-008-f10 LT0032 Homo sapiens cDNA, mRNA sequence	1,29
22920	KIFAP3	kinesin-associated protein 3	1,28
	ILMN_125302	RST26545 Athersys RAGE Library Homo sapiens cDNA, mRNA sequence	1,28
1122	CHML	choroideremia-like (Rab escort protein 2)	1,28
10095	ARPC1B	actin related protein 2/3 complex, subunit 1B, 41kDa	1,28
55122	AKIRIN2	akirin 2	1,28
117177	ILMN_41802	Homo sapiens RAB3A interacting protein (rabin3) (RAB3IP), transcript variant A, mRNA.	1,27
728127	ILMN_46425	PREDICTED: Homo sapiens similar to centaurin, gamma-like family, member 1 (LOC728127), mRNA.	1,27
388555	ILMN_17801	Homo sapiens IGF-like family member 3 (IGFL3), mRNA.	1,27
440712	ILMN_20193	Homo sapiens chromosome 1 open reading frame 186 (C1orf186), mRNA.	1,27
137492	VPS37A	vacuolar protein sorting 37 homolog A (S. cerevisiae)	1,26
140739	UBE2F	ubiquitin-conjugating enzyme E2F (putative)	1,26

641649	TMEM91	transmembrane protein 91	1,26
27020	NPTN	neuroplastin	1,26
143666	ILMN_28031	PREDICTED: Homo sapiens hypothetical protein LOC143666 (LOC143666), mRNA.	1,26
	ILMN_118124	BP395220 Homo sapiens pancreatic islet Homo sapiens cDNA clone hbt03885 3, mRNA sequence	1,26
8365	HIST1H4H	histone cluster 1, H4h	1,26
7064	THOP1	thimet oligopeptidase 1	1,25
643154	ILMN_38879	PREDICTED: Homo sapiens hypothetical protein LOC643154 (LOC643154), mRNA.	1,25
7456	WIPF1	WAS/WASL interacting protein family, member 1	1,24
27040	LAT	linker for activation of T cells	1,24
	ILMN_105596	hd01g11.x1 Soares_NFL_T_GBC_S1 Homo sapiens cDNA clone IMAGE:2908292 3 similar to contains L1.t2 L1 repetitive element ;, mRNA sequence	1,24
2820	GPD2	glycerol-3-phosphate dehydrogenase 2 (mitochondrial)	1,24
7292	TNFSF4	tumor necrosis factor (ligand) superfamily, member 4	1,23
8926	ILMN_15998	Homo sapiens SNRPN upstream reading frame (SNURF), transcript variant 1, mRNA.	1,23
5494	PPM1A	protein phosphatase, Mg2+/Mn2+ dependent, 1A	1,23
25915	NDUFAF3	NADH dehydrogenase (ubiquinone) 1 alpha subcomplex, assembly factor 3	1,23
4154	MBNL1	muscleblind-like (Drosophila)	1,23
	ILMN_89656	yz74c09.s1 Soares_multiple_sclerosis_2NbHMSP Homo sapiens cDNA clone IMAGE:288784 3, mRNA sequence	1,23
2207	FCER1G	Fc fragment of IgE, high affinity I, receptor for; gamma polypeptide	1,23
7401	CLRN1	clarin 1	1,23
1071	CETP	cholesteryl ester transfer protein, plasma	1,23
51538	ZCCHC17	zinc finger, CCHC domain containing 17	1,22
6683	SPAST	spastin	1,22
6478	SIAH2	seven in absentia homolog 2 (Drosophila)	1,22
5110	PCMT1	protein-L-isoaspartate (D-aspartate) O-methyltransferase	1,22
642489	ILMN_37913	PREDICTED: Homo sapiens similar to FK506-binding protein 1A (LOC642489), mRNA.	1,22
3549	IHH	Indian hedgehog	1,22
2500	ILMN_16099	Homo sapiens ferritin, heavy polypeptide-like 7 (FTHL7) on chromosome 13.	1,22
574	ILMN_10365	Homo sapiens B melanoma antigen (BAGE), mRNA.	1,22
	ILMN_71879	Homo sapiens cDNA clone IMAGE:5297467	1,21
143279	ILMN_23234	Homo sapiens HECT domain containing 2 (HECTD2), transcript variant 2, mRNA.	1,21
6451	SH3BGRL	SH3 domain binding glutamic acid-rich protein like	1,2
5567	PRKACB	protein kinase, cAMP-dependent, catalytic, beta	1,2

4917	NTN3	netrin 3	1,2
112597	ILMN_137135	Homo sapiens hypothetical protein MGC4677 (MGC4677), mRNA.	1,2
	ILMN_90731	UI-H-FG0-bcw-h-18-0-UI.s1 NCI_CGAP_EN1_2 Homo sapiens cDNA clone UI-H-FG0-bcw-h-18-0-UI 3, mRNA sequence	1,2
4735	2.IX	septin 2	1,19
644010	ILMN_41564	PREDICTED: Homo sapiens hypothetical LOC644010, transcript variant 2 (LOC644010), mRNA.	1,19
85236	HIST1H2BK	histone cluster 1, H2bk	1,19
8344	HIST1H2BE	histone cluster 1, H2be	1,19
2869	GRK5	G protein-coupled receptor kinase 5	1,19
219771	CCNY	cyclin Y	1,19
896	CCND3	cyclin D3	1,19
1175	AP2S1	adaptor-related protein complex 2, sigma 1 subunit	1,19
93650	ACPT	acid phosphatase, testicular	1,19
7511	XPNPEP1	X-prolyl aminopeptidase (aminopeptidase P) 1, soluble	1,18
5257	PHKB	phosphorylase kinase, beta	1,18
651589	ILMN_32438	PREDICTED: Homo sapiens similar to SET domain and mariner transposase fusion gene (LOC651589), mRNA.	1,18
644322	ILMN_34524	PREDICTED: Homo sapiens similar to Ribosome biogenesis protein BMS1 homolog, transcript variant 2 (LOC644322), mRNA.	1,18
22876	INPP5F	inositol polyphosphate-5-phosphatase F	1,18
3133	HLA-E	major histocompatibility complex, class I, E	1,18
8324	FZD7	frizzled family receptor 7	1,18
29940	DSE	dermatan sulfate epimerase	1,18
152065	C3orf22	chromosome 3 open reading frame 22	1,18
7054	TH	tyrosine hydroxylase	1,17
10427	ILMN_138457	PREDICTED: Homo sapiens SEC24 related gene family, member B (S. cerevisiae), transcript variant 3 (SEC24B), mRNA.	1,17
5473	PPBP	pro-platelet basic protein (chemokine (C-X-C motif) ligand 7)	1,17
5341	PLEK	pleckstrin	1,17
56890	MDM1	Mdm1 nuclear protein homolog (mouse)	1,17
387820	ILMN_26714	PREDICTED: Homo sapiens similar to DnaJ (Hsp40) homolog, subfamily B, member 6 isoform a (LOC387820), mRNA.	1,17
2776	GNAQ	guanine nucleotide binding protein (G protein), q polypeptide	1,17
54918	CMTM6	CKLF-like MARVEL transmembrane domain containing 6	1,17
7739	ZNF185	zinc finger protein 185 (LIM domain)	1,16

8764	TNFRSF14	tumor necrosis factor receptor superfamily, member 14	1,16
8654	PDE5A	phosphodiesterase 5A, cGMP-specific	1,16
400948	ILMN_32897	PREDICTED: Homo sapiens similar to CG33774-PA (LOC400948), mRNA.	1,16
55683	KIAA1310	KIAA1310	1,16
239	ALOX12	arachidonate 12-lipoxygenase	1,16
283131	ILMN_138191	PREDICTED: Homo sapiens trophoblast-derived noncoding RNA (TncRNA), misc RNA.	1,15
100131943	LOC100131943	hypothetical LOC100131943	1,15
10357	HMGB1P1	high mobility group box 1 pseudogene 1	1,15
65056	GPBP1	GC-rich promoter binding protein 1	1,15
2280	FKBP1A	FK506 binding protein 1A, 12kDa	1,15
5782	PTPN12	protein tyrosine phosphatase, non-receptor type 12	1,14
9200	PTPLA	protein tyrosine phosphatase-like (proline instead of catalytic arginine), member A	1,14
29074	MRPL18	mitochondrial ribosomal protein L18	1,14
4149	MAX	MYC associated factor X	1,14
402221	ILMN_35678	PREDICTED: Homo sapiens similar to actin alpha 1 skeletal muscle protein (LOC402221), mRNA.	1,14
3953	LEPR	leptin receptor	1,14
	ILMN_86083	602274281F1 NIH_MGC_85 Homo sapiens cDNA clone IMAGE:4362141 5, mRNA sequence	1,14
81631	MAP1LC3B	microtubule-associated protein 1 light chain 3 beta	1,13
727958	ILMN_35093	PREDICTED: Homo sapiens hypothetical protein LOC727958 (LOC727958), mRNA.	1,13
646135	ILMN_39828	PREDICTED: Homo sapiens hypothetical protein LOC646135 (LOC646135), mRNA.	1,13
442204	ILMN_34044	PREDICTED: Homo sapiens similar to myosin regulatory light chain-like (LOC442204), mRNA.	1,13
	ILMN_92185	602849957F1 NIH_MGC_10 Homo sapiens cDNA clone IMAGE:4991510 5, mRNA sequence	1,13
9191	DEDD	death effector domain containing	1,13
527	ATP6V0C	ATPase, H+ transporting, lysosomal 16kDa, V0 subunit c	1,13
427	ASAH1	N-acylsphingosine amidohydrolase (acid ceramidase) 1	1,13
80177	MYCT1	myc target 1	1,12
	ILMN_100075	Homo sapiens cDNA FLJ37325 fis, clone BRAMY2018295	1,12
54435	ILMN_42290	Homo sapiens HLA complex group 4 (HCG4), non-coding RNA.	1,12
399886	ILMN_1487	Homo sapiens FLJ41423 protein (FLJ41423), mRNA.	1,12
51185	CRBN	cereblon	1,12
79752	ILMN_23674	Homo sapiens zinc finger, AN1-type domain 1 (ZFAND1), mRNA.	1,11
339005	ILMN_32270	Homo sapiens WAS protein homolog associated with actin, golgi membranes and microtubules-like 1 (WHAMML1), non-coding RNA.	1,11

124590	USH1G	Usher syndrome 1G (autosomal recessive)	1,11
53346	TM6SF1	transmembrane 6 superfamily member 1	1,11
23061	ILMN_9169	Homo sapiens TBC1 domain family, member 9B (with GRAM domain) (TBC1D9B), transcript variant 2, mRNA.	1,11
4201	MEA1	male-enhanced antigen 1	1,11
	ILMN_90317	Homo sapiens cDNA FLJ32550 fis, clone SPLEN1000056	1,11
2495	FTH1	ferritin, heavy polypeptide 1	1,11
54967	ILMN_138751	PREDICTED: Homo sapiens chromosome X open reading frame 48 (CXorf48), mRNA.	1,11
6352	CCL5	chemokine (C-C motif) ligand 5	1,11
55744	C7orf44	chromosome 7 open reading frame 44	1,11
408	ARRB1	arrestin, beta 1	1,11
10000	AKT3	v-akt murine thymoma viral oncogene homolog 3 (protein kinase B, gamma)	1,11
157680	VPS13B	vacuolar protein sorting 13 homolog B (yeast)	1,1
22925	ILMN_22699	Homo sapiens phospholipase A2 receptor 1, 180kDa (PLA2R1), transcript variant 2, mRNA.	1,1
651723	ILMN_39567	PREDICTED: Homo sapiens similar to schlafen family member 13 (LOC651723), mRNA.	1,1
644642	ILMN_43268	PREDICTED: Homo sapiens hypothetical protein LOC644642 (LOC644642), mRNA.	1,1
89857	KLHL6	kelch-like 6 (Drosophila)	1,1
2273	FHL1	four and a half LIM domains 1	1,1
286006	C7orf53	chromosome 7 open reading frame 53	1,1
84886	C1orf198	chromosome 1 open reading frame 198	1,1
1822	ATN1	atrophin 1	1,1
8751	ILMN_138255	PREDICTED: Homo sapiens ADAM metallopeptidase domain 15 (metargidin) (ADAM15), mRNA.	1,1
79752	ILMN_23674	Homo sapiens zinc finger, AN1-type domain 1 (ZFAND1), mRNA.	1,09
7531	YWHAE	tyrosine 3-monooxygenase/tryptophan 5-monooxygenase activation protein, epsilon polypeptide	1,09
5870	RAB6A	RAB6A, member RAS oncogene family	1,09
9861	PSMD6	proteasome (prosome, macropain) 26S subunit, non-ATPase, 6	1,09
653650	ILMN_46005	PREDICTED: Homo sapiens similar to 3-phosphoinositide dependent protein kinase 1 (hPDK1), transcript variant 6 (LOC653650), mRNA.	1,09
3802	ILMN_30269	Homo sapiens killer cell immunoglobulin-like receptor, two domains, long cytoplasmic tail, 1 (KIR2DL1), mRNA.	1,09
10553	ILMN_16008	Homo sapiens HIV-1 Tat interactive protein 2, 30kDa (HTATIP2), mRNA.	1,09
	ILMN_113901	BX088674 Soares_testis_NHT Homo sapiens cDNA clone IMAGp998A143518, mRNA sequence	1,09
	ILMN_106290	xc76e10.x1 NCI_CGAP_Ov32 Homo sapiens cDNA clone IMAGE:2590218 3, mRNA sequence	1,09
	ILMN_103797	Homo sapiens cDNA clone IMAGE:5260685	1,09
760	CA2	carbonic anhydrase II	1,09

57038	RARS2	arginyl-tRNA synthetase 2, mitochondrial	1,08
651643	ILMN_39968	PREDICTED: Homo sapiens hypothetical protein LOC651643 (LOC651643), mRNA.	1,08
387882	ILMN_23241	Homo sapiens hypothetical protein (LOC387882), mRNA.	1,08
	ILMN_87232	UI-E-EJ0-ahk-m-09-0-UI.r2 UI-E-EJ0 Homo sapiens cDNA clone UI-E-EJ0-ahk-m-09-0-UI 5, mRNA sequence	1,08
1647	GADD45A	growth arrest and DNA-damage-inducible, alpha	1,08
391059	FRRS1	ferric-chelate reductase 1	1,08
51582	AZIN1	antizyme inhibitor 1	1,08
27072	VPS41	vacuolar protein sorting 41 homolog (S. cerevisiae)	1,07
9342	SNAP29	synaptosomal-associated protein, 29kDa	1,07
9963	SLC23A1	solute carrier family 23 (nucleobase transporters), member 1	1,07
8031	NCOA4	nuclear receptor coactivator 4	1,07
730077	ILMN_39361	PREDICTED: Homo sapiens similar to NEW1 domain containing protein isoform 1 (LOC730077), mRNA.	1,07
56940	ILMN_138115	PREDICTED: Homo sapiens dual specificity phosphatase 22 (DUSP22), mRNA.	1,07
10257	ABCC4	ATP-binding cassette, sub-family C (CFTR/MRP), member 4	1,07
339005	WHAMMP3	WAS protein homolog associated with actin, golgi membranes and microtubules pseudogene 3	1,06
25816	TNFAIP8	tumor necrosis factor, alpha-induced protein 8	1,06
23512	SUZ12	suppressor of zeste 12 homolog (Drosophila)	1,06
115286	ILMN_15118	Homo sapiens solute carrier family 25, member 26 (SLC25A26), transcript variant 3, mRNA.	1,06
5573	PRKAR1A	protein kinase, cAMP-dependent, regulatory, type I, alpha (tissue specific extinguisher 1)	1,06
29780	PARVB	parvin, beta	1,06
8736	MYOM1	myomesin 1, 185kDa	1,06
4585	ILMN_138880	PREDICTED: Homo sapiens mucin 4, tracheobronchial (MUC4), mRNA.	1,06
136319	MTPN	myotrophin	1,06
729003	ILMN_40608	PREDICTED: Homo sapiens hypothetical protein LOC729003 (LOC729003), mRNA.	1,06
2537	IFI6	interferon, alpha-inducible protein 6	1,06
	ILMN_128394	DB341695 TESTI4 Homo sapiens cDNA clone TESTI4045781 3, mRNA sequence	1,06
	ILMN_122627	ai16e07.s1 Soares_parathyroid_tumor_NbHPA Homo sapiens cDNA clone 1342980 3, mRNA sequence	1,06
	ILMN_85871	nu88b06.s1 NCI_CGAP_Alv1 Homo sapiens cDNA clone IMAGE:1217747, mRNA sequence	1,06
3005	H1F0	H1 histone family, member 0	1,06
1603	DAD1	defender against cell death 1	1,06
51433	ANAPC5	anaphase promoting complex subunit 5	1,06
6845	VAMP7	vesicle-associated membrane protein 7	1,05
6001	RGS10	regulator of G-protein signaling 10	1,05

347168	OR1J1	olfactory receptor, family 1, subfamily J, member 1	1,05
647000	ILMN_45576	PREDICTED: Homo sapiens similar to tubulin, beta 5 (LOC647000), mRNA.	1,05
	ILMN_130543	DB038017 TESTI2 Homo sapiens cDNA clone TESTI2024179 5, mRNA sequence	1,05
2287	FKBP3	FK506 binding protein 3, 25kDa	1,05
9637	FEZ2	fasciculation and elongation protein zeta 2 (zygin II)	1,05
4735	2.IX	septin 2	1,04
6016	RIT1	Ras-like without CAAX 1	1,04
84281	C2orf88	chromosome 2 open reading frame 88	1,04
	ILMN_73823	Homo sapiens cDNA: FLJ23006 fis, clone LNG00414	1,04
	ILMN_117904	UI-H-BW1-anw-a-06-0-UI.s1 NCI_CGAP_Sub7 Homo sapiens cDNA clone IMAGE:3083435 3, mRNA sequence	1,04
649181	ILMN_41226	PREDICTED: Homo sapiens hypothetical protein LOC649181 (LOC649181), mRNA.	1,03
	ILMN_81181	Homo sapiens cDNA FLJ36653 fis, clone UTERU2001176	1,03
2852	GPER	G protein-coupled estrogen receptor 1	1,03
1948	EFNB2	ephrin-B2	1,03
10059	DNM1L	dynamin 1-like	1,03
27071	DAPP1	dual adaptor of phosphotyrosine and 3-phosphoinositides	1,03
55256	ADI1	acireductone dioxygenase 1	1,03
23032	USP33	ubiquitin specific peptidase 33	1,02
6451	SH3BGRL	SH3 domain binding glutamic acid-rich protein like	1,02
11031	RAB31	RAB31, member RAS oncogene family	1,02
10611	PDLIM5	PDZ and LIM domain 5	1,02
122773	KLHDC1	kelch domain containing 1	1,02
3811	KIR3DL1	killer cell immunoglobulin-like receptor, three domains, long cytoplasmic tail, 1	1,02
10082	GPC6	glypican 6	1,02
1859	ILMN_19737	Homo sapiens dual-specificity tyrosine-(Y)-phosphorylation regulated kinase 1A (DYRK1A), transcript variant 3, mRNA.	1,02
7402	UTRN	utrophin	1,01
400952	ILMN_36207	PREDICTED: Homo sapiens NGNL6975 (UNQ6975), mRNA.	1,01
653463	ILMN_31314	PREDICTED: Homo sapiens similar to hypothetical gene supported by AK127120 (LOC653463), mRNA.	1,01
100288367	LOC100288367	golgin A2 pseudogene	1,01
57461	ISY1	ISY1 splicing factor homolog (S. cerevisiae)	1,01
	ILMN_106734	xc23f04.x1 NCI_CGAP_Co19 Homo sapiens cDNA clone IMAGE:2585119 3, mRNA sequence	1,01
	ILMN_76886	Homo sapiens cDNA FLJ42306 fis, clone TRACH2001646	1,01

3190	HNRNPK	heterogeneous nuclear ribonucleoprotein K	1,01
28996	HIPK2	homeodomain interacting protein kinase 2	1,01
55207	ARL8B	ADP-ribosylation factor-like 8B	1,01
57552	NCEH1	neutral cholesterol ester hydrolase 1	1,01
6399	ILMN_558	Homo sapiens trafficking protein particle complex 2 (TRAPPC2), transcript variant 2, mRNA.	1
64849	ILMN_13280	Homo sapiens solute carrier family 13 (sodium-dependent dicarboxylate transporter), member 3 (SLC13A3), transcript variant 1, mRNA.	1
84236	RHBDD1	rhomboid domain containing 1	1
653136	ILMN_39035	PREDICTED: Homo sapiens similar to IFP38 (LOC653136), mRNA.	1
649897	ILMN_45446	PREDICTED: Homo sapiens similar to Ig gamma-2 chain C region (LOC649897), mRNA.	1
	ILMN_119248	UI-E-CK1-agb-m-11-0-UI.s1 UI-E-CK1 Homo sapiens cDNA clone UI-E-CK1-agb-m-11-0-UI 3, mRNA sequence	1
85865	ILMN_166088	Homo sapiens GTP-binding protein 10 (putative) (GTPBP10), transcript variant 1, mRNA.	1
2733	GLE1	GLE1 RNA export mediator homolog (yeast)	1
375061	FAM89A	family with sequence similarity 89, member A	1
26298	EHF	ets homologous factor	1
402	ARL2	ADP-ribosylation factor-like 2	1
6181	RPLP2	ribosomal protein, large, P2	-1
6135	RPL11	ribosomal protein L11	-1
55629	ILMN_7879	Homo sapiens proline-rich nuclear receptor coactivator 2 (PNRC2), mRNA.	-1
10135	NAMPT	nicotinamide phosphoribosyltransferase	-1
400464	ILMN_15604	Homo sapiens similar to FLJ43276 protein (LOC400464), mRNA.	-1
10818	FRS2	fibroblast growth factor receptor substrate 2	-1
1236	CCR7	chemokine (C-C motif) receptor 7	-1
79630	C1orf54	chromosome 1 open reading frame 54	-1
64422	ATG3	ATG3 autophagy related 3 homolog (S. cerevisiae)	-1
51385	ZNF589	zinc finger protein 589	-1,01
7874	USP7	ubiquitin specific peptidase 7 (herpes virus-associated)	-1,01
7045	TGFBI	transforming growth factor, beta-induced, 68kDa	-1,01
57466	SCAF4	SR-related CTD-associated factor 4	-1,01
6281	S100A10	S100 calcium binding protein A10	-1,01
29108	PYCARD	PYD and CARD domain containing	-1,01
51317	PHF21A	PHD finger protein 21A	-1,01
51199	NIN	ninein (GSK3B interacting protein)	-1,01

728734	ILMN_12371	PREDICTED: Homo sapiens similar to kidney-specific protein (KS), transcript variant 1 (LOC728734), mRNA.	-1,01
653226	SRP9P1	signal recognition particle 9 pseudogene 1	-1,01
643920	ILMN_39946	PREDICTED: Homo sapiens region containing similar to Williams Beuren syndrome chromosome region 19; hypothetical LOC441257, transcript variant 2 (LOC643920), mRNA.	-1,01
	ILMN_107450	UI-CF-DU1-aag-j-05-0-UI.s1 UI-CF-DU1 Homo sapiens cDNA clone UI-CF-DU1-aag-j-05-0-UI 3, mRNA sequence	-1,01
2841	GPR18	G protein-coupled receptor 18	-1,01
285527	FRYL	FRY-like	-1,01
115761	ARL11	ADP-ribosylation factor-like 11	-1,01
7259	TSPYL1	TSPY-like 1	-1,02
6921	TCEB1	transcription elongation factor B (SIII), polypeptide 1 (15kDa, elongin C)	-1,02
6277	S100A6	S100 calcium binding protein A6	-1,02
4144	MAT2A	methionine adenosyltransferase II, alpha	-1,02
8939	FUBP3	far upstream element (FUSE) binding protein 3	-1,02
22837	COBLL1	COBL-like 1	-1,02
56683	C21orf59	chromosome 21 open reading frame 59	-1,02
79090	TRAPPC6A	trafficking protein particle complex 6A	-1,03
54968	TMEM70	transmembrane protein 70	-1,03
9491	PSMF1	proteasome (prosome, macropain) inhibitor subunit 1 (PI31)	-1,03
10621	ILMN_5317	Homo sapiens polymerase (RNA) III (DNA directed) polypeptide F, 39 kDa (POLR3F), mRNA.	-1,03
653506	ILMN_30995	PREDICTED: Homo sapiens similar to meteorin, glial cell differentiation regulator-like (LOC653506), mRNA.	-1,03
2999	GZMH	granzyme H (cathepsin G-like 2, protein h-CCPX)	-1,03
9535	GMFG	glia maturation factor, gamma	-1,03
79571	GCC1	GRIP and coiled-coil domain containing 1	-1,03
1736	DKC1	dyskeratosis congenita 1, dyskerin	-1,03
79012	CAMKV	CaM kinase-like vesicle-associated	-1,03
388078	ILMN_31332	PREDICTED: Homo sapiens V-set and immunoglobulin domain containing 6 (VSIG6), mRNA.	-1,04
706	TSPO	translocator protein (18kDa)	-1,04
6902	TBCA	tubulin folding cofactor A	-1,04
26801	SNORD48	small nucleolar RNA, C/D box 48	-1,04
29964	PRICKLE4	prickle homolog 4 (Drosophila)	-1,04
126382	NR2C2AP	nuclear receptor 2C2-associated protein	-1,04
3482	IGF2R	insulin-like growth factor 2 receptor	-1,04
112398	EGLN2	egl nine homolog 2 (C. elegans)	-1,04

10294	DNAJA2	DnaJ (Hsp40) homolog, subfamily A, member 2	-1,04
28969	BZW2	basic leucine zipper and W2 domains 2	-1,04
54499	TMCO1	transmembrane and coiled-coil domains 1	-1,05
6726	SRP9	signal recognition particle 9kDa	-1,05
51728	POLR3K	polymerase (RNA) III (DNA directed) polypeptide K, 12.3 kDa	-1,05
6147	RPL23A	ribosomal protein L23a	-1,05
	ILMN_104476	zj45e10.s1 Soares_fetal_liver_spleen_1NFLS_S1 Homo sapiens cDNA clone IMAGE:453258 3, mRNA sequence	-1,05
2353	FOS	FBJ murine osteosarcoma viral oncogene homolog	-1,05
539	ATP5O	ATP synthase, H+ transporting, mitochondrial F1 complex, O subunit	-1,05
7133	TNFRSF1B	tumor necrosis factor receptor superfamily, member 1B	-1,06
92241	RCSD1	RCSD domain containing 1	-1,06
54625	PARP14	poly (ADP-ribose) polymerase family, member 14	-1,06
11165	NUDT3	nudix (nucleoside diphosphate linked moiety X)-type motif 3	-1,06
64231	ILMN_2009	Homo sapiens membrane-spanning 4-domains, subfamily A, member 6A (MS4A6A), transcript variant 2, mRNA.	-1,06
134997	ILMN_1017	Homo sapiens peptidylprolyl isomerase A processed pseudogene (LOC134997) on chromosome 6.	-1,06
728358	ILMN_11220	Homo sapiens defensin, alpha 1B (DEFA1B), mRNA.	-1,06
64225	ATL2	atlastin GTPase 2	-1,06
23370	ARHGEF18	Rho/Rac guanine nucleotide exchange factor (GEF) 18	-1,06
200316	ILMN_1659	Homo sapiens apolipoprotein B mRNA editing enzyme, catalytic polypeptide-like 3F (APOBEC3F), transcript variant 1, mRNA.	-1,06
6817	ILMN_28965	Homo sapiens sulfotransferase family, cytosolic, 1A, phenol-preferring, member 1 (SULT1A1), transcript variant 5, mRNA.	-1,07
3716	JAK1	Janus kinase 1	-1,07
220988	HNRNPA3	heterogeneous nuclear ribonucleoprotein A3	-1,07
9637	FEZ2	fasciculation and elongation protein zeta 2 (zygin II)	-1,07
1327	COX4I1	cytochrome c oxidase subunit IV isoform 1	-1,07
56994	CHPT1	choline phosphotransferase 1	-1,07
805	CALM2	calmodulin 2 (phosphorylase kinase, delta)	-1,07
55813	UTP6	UTP6, small subunit (SSU) processome component, homolog (yeast)	-1,08
7329	UBE2I	ubiquitin-conjugating enzyme E2I	-1,08
7267	TTC3	tetratricopeptide repeat domain 3	-1,08
8148	ILMN_18165	Homo sapiens TAF15 RNA polymerase II, TATA box binding protein (TBP)-associated factor, 68kDa (TAF15), transcript variant 2, mRNA.	-1,08

30968	STOML2	stomatin (EPB72)-like 2	-1,08
55505	NOP10	NOP10 ribonucleoprotein homolog (yeast)	-1,08
4191	MDH2	malate dehydrogenase 2, NAD (mitochondrial)	-1,08
3181	HNRNPA2B1	heterogeneous nuclear ribonucleoprotein A2/B1	-1,08
3043	HBB	hemoglobin, beta	-1,08
2665	GDI2	GDP dissociation inhibitor 2	-1,08
55140	ELP3	elongation protein 3 homolog (S. cerevisiae)	-1,08
51596	CUTA	cutA divalent cation tolerance homolog (E. coli)	-1,08
55101	ILMN_12644	Homo sapiens ATP5S-like (ATP5SL), mRNA.	-1,08
7178	TPT1	tumor protein, translationally-controlled 1	-1,09
6612	SUMO3	SMT3 suppressor of mif two 3 homolog 3 (S. cerevisiae)	-1,09
51065	RPS27L	ribosomal protein S27-like	-1,09
5500	PPP1CB	protein phosphatase 1, catalytic subunit, beta isozyme	-1,09
5216	PFN1	profilin 1	-1,09
64324	NSD1	nuclear receptor binding SET domain protein 1	-1,09
400818	ILMN_26956	Homo sapiens neuroblastoma breakpoint family, member 20 (NBPF20), mRNA.	-1,09
64951	MRPS24	mitochondrial ribosomal protein S24	-1,09
285053	ILMN_44464	PREDICTED: Homo sapiens similar to ribosomal protein L18a, transcript variant 1 (LOC285053), mRNA.	-1,09
56943	ENY2	enhancer of yellow 2 homolog (Drosophila)	-1,09
55661	DDX27	DEAD (Asp-Glu-Ala-Asp) box polypeptide 27	-1,09
10658	ILMN_10496	Homo sapiens CUG triplet repeat, RNA binding protein 1 (CUGBP1), transcript variant 2, mRNA.	-1,09
1396	CRIP1	cysteine-rich protein 1 (intestinal)	-1,09
929	CD14	CD14 molecule	-1,09
22809	ATF5	activating transcription factor 5	-1,09
8795	TNFRSF10B	tumor necrosis factor receptor superfamily, member 10b	-1,1
292	SLC25A5	solute carrier family 25 (mitochondrial carrier; adenine nucleotide translocator), member 5	-1,1
6390	SDHB	succinate dehydrogenase complex, subunit B, iron sulfur (Ip)	-1,1
441502	RPS26P11	ribosomal protein S26 pseudogene 11	-1,1
5705	PSMC5	proteasome (prosome, macropain) 26S subunit, ATPase, 5	-1,1
5538	PPT1	palmitoyl-protein thioesterase 1	-1,1
6187	RPS2	ribosomal protein S2	-1,1
440341	ILMN_34066	PREDICTED: Homo sapiens similar to nuclear pore complex interacting protein, transcript variant 4 (LOC440341), mRNA.	-1,1

3178	HNRNPA1	heterogeneous nuclear ribonucleoprotein A1	-1,1
3065	HDAC1	histone deacetylase 1	-1,1
2948	ILMN_4003	Homo sapiens glutathione S-transferase M4 (GSTM4), transcript variant 2, mRNA.	-1,1
1520	ILMN_13149	Homo sapiens cathepsin S (CTSS), mRNA.	-1,1
150684	COMMD1	copper metabolism (Murr1) domain containing 1	-1,1
23468	CBX5	chromobox homolog 5	-1,1
389541	C7orf59	chromosome 7 open reading frame 59	-1,1
689	BTF3	basic transcription factor 3	-1,1
9938	ARHGAP25	Rho GTPase activating protein 25	-1,1
7247	TSN	translin	-1,11
7127	ILMN_16372	Homo sapiens tumor necrosis factor, alpha-induced protein 2 (TNFAIP2), mRNA.	-1,11
10287	RGS19	regulator of G-protein signaling 19	-1,11
5660	PSAP	prosaposin	-1,11
118788	PIK3AP1	phosphoinositide-3-kinase adaptor protein 1	-1,11
9284	ILMN_10143	Homo sapiens nuclear pore complex interacting protein (NPIP), mRNA.	-1,11
	ILMN_132410	AV735490 CB Homo sapiens cDNA clone CBFBDD05 5, mRNA sequence	-1,11
54541	DDIT4	DNA-damage-inducible transcript 4	-1,11
1642	DDB1	damage-specific DNA binding protein 1, 127kDa	-1,11
1540	ILMN_28818	Homo sapiens cylindromatosis (turban tumor syndrome) (CYLD), mRNA.	-1,11
919	CD247	CD247 molecule	-1,11
908	CCT6A	chaperonin containing TCP1, subunit 6A (zeta 1)	-1,11
865	CBFB	core-binding factor, beta subunit	-1,11
9584	RBM39	RNA binding motif protein 39	-1,12
5788	PTPRC	protein tyrosine phosphatase, receptor type, C	-1,12
2782	GNB1	guanine nucleotide binding protein (G protein), beta polypeptide 1	-1,12
9158	FIBP	fibroblast growth factor (acidic) intracellular binding protein	-1,12
1667	ILMN_29692	Homo sapiens defensin, alpha 1 (DEFA1), mRNA.	-1,12
56993	TOMM22	translocase of outer mitochondrial membrane 22 homolog (yeast)	-1,13
4666	NACA	nascent polypeptide-associated complex alpha subunit	-1,13
7462	LAT2	linker for activation of T cells family, member 2	-1,13
3059	HCLS1	hematopoietic cell-specific Lyn substrate 1	-1,13
1340	COX6B1	cytochrome c oxidase subunit VIb polypeptide 1 (ubiquitous)	-1,13
1043	CD52	CD52 molecule	-1,13

148789	B3GALNT2	beta-1,3-N-acetylgalactosaminyltransferase 2	-1,13
199	AIF1	allograft inflammatory factor 1	-1,13
5250	SLC25A3	solute carrier family 25 (mitochondrial carrier; phosphate carrier), member 3	-1,14
11040	ILMN_27932	Homo sapiens pim-2 oncogene (PIM2), mRNA.	-1,14
4722	NDUFS3	NADH dehydrogenase (ubiquinone) Fe-S protein 3, 30kDa (NADH-coenzyme Q reductase)	-1,14
650029	ILMN_38295	PREDICTED: Homo sapiens similar to RNA-binding protein 4 (RNA-binding motif protein 4) (Lark homolog) (Hlark) (RNA-binding motif protein 4a), transcript variant 1 (LOC650029), mRNA.	-1,14
644131	CCT8P1	chaperonin containing TCP1, subunit 8 (theta) pseudogene 1	-1,14
641996	ILMN_32260	PREDICTED: Homo sapiens hypothetical protein LOC641996 (LOC641996), mRNA.	-1,14
284194	LGALS9B	lectin, galactoside-binding, soluble, 9B	-1,14
3683	ILMN_15930	Homo sapiens integrin, alpha L (antigen CD11A (p180), lymphocyte function-associated antigen 1; alpha polypeptide) (ITGAL), mRNA.	-1,14
1668	ILMN_11220	Homo sapiens defensin, alpha 3, neutrophil-specific (DEFA3), mRNA.	-1,14
11325	DDX42	DEAD (Asp-Glu-Ala-Asp) box polypeptide 42	-1,14
6138	RPL15	ribosomal protein L15	-1,15
149628	PYHIN1	pyrin and HIN domain family, member 1	-1,15
648840	ILMN_37663	PREDICTED: Homo sapiens hypothetical protein LOC648840 (LOC648840), mRNA.	-1,15
6141	RPL18	ribosomal protein L18	-1,15
	ILMN_83371	full-length cDNA clone CS0DC013YI10 of Neuroblastoma Cot 25-normalized of Homo sapiens (human)	-1,15
9170	LPAR2	lysophosphatidic acid receptor 2	-1,15
55619	DOCK10	dedicator of cytokinesis 10	-1,15
159	ADSS	adenylosuccinate synthase	-1,15
51522	TMEM14C	transmembrane protein 14C	-1,16
25949	SYF2	SYF2 homolog, RNA splicing factor (S. cerevisiae)	-1,16
6122	ILMN_29090	Homo sapiens ribosomal protein L3 (RPL3), transcript variant 2, mRNA.	-1,16
64326	RFWD2	ring finger and WD repeat domain 2	-1,16
83937	RASSF4	Ras association (RalGDS/AF-6) domain family member 4	-1,16
3543	IGLL1	immunoglobulin lambda-like polypeptide 1	-1,16
10542	HBXIP	hepatitis B virus x interacting protein	-1,16
1653	DDX1	DEAD (Asp-Glu-Ala-Asp) box polypeptide 1	-1,16
689	BTF3	basic transcription factor 3	-1,16
8125	ANP32A	acidic (leucine-rich) nuclear phosphoprotein 32 family, member A	-1,16
10285	SMNDC1	survival motor neuron domain containing 1	-1,17

6176	RPLP1	ribosomal protein, large, P1	-1,17
6885	MAP3K7	mitogen-activated protein kinase kinase kinase 7	-1,17
644037	HNRNPA1P12	heterogeneous nuclear ribonucleoprotein A1 pseudogene 12	-1,17
9807	IP6K1	inositol hexakisphosphate kinase 1	-1,17
	ILMN_108776	od80c09.s1 NCI_CGAP_Ov2 Homo sapiens cDNA clone IMAGE:1374256, mRNA sequence	-1,17
2217	FCGRT	Fc fragment of IgG, receptor, transporter, alpha	-1,17
441168	FAM26F	family with sequence similarity 26, member F	-1,17
57609	DIP2B	DIP2 disco-interacting protein 2 homolog B (Drosophila)	-1,17
301	ANXA1	annexin A1	-1,17
55754	TMEM30A	transmembrane protein 30A	-1,18
255758	TCTEX1D2	Tctex1 domain containing 2	-1,18
8893	EIF2B5	eukaryotic translation initiation factor 2B, subunit 5 epsilon, 82kDa	-1,18
1075	CTSC	cathepsin C	-1,18
868	CBLB	Cas-Br-M (murine) ecotropic retroviral transforming sequence b	-1,18
10476	ATP5H	ATP synthase, H+ transporting, mitochondrial Fo complex, subunit d	-1,18
515	ATP5F1	ATP synthase, H+ transporting, mitochondrial Fo complex, subunit B1	-1,18
7431	VIM	vimentin	-1,19
57050	UTP3	UTP3, small subunit (SSU) processome component, homolog (S. cerevisiae)	-1,19
8675	STX16	syntaxin 16	-1,19
645317	ILMN_15841	PREDICTED: Homo sapiens similar to Coiled-coil-helix-coiled-coil-helix domain-containing protein 2 (HCV NS2 trans-regulated protein) (NS2TP), transcript variant 3 (LOC645317), mRNA.	-1,19
27246	RNF115	ring finger protein 115	-1,2
51506	UFC1	ubiquitin-fold modifier conjugating enzyme 1	-1,2
6154	RPL26	ribosomal protein L26	-1,2
5293	PIK3CD	phosphoinositide-3-kinase, catalytic, delta polypeptide	-1,2
55303	GIMAP4	GTPase, IMAP family member 4	-1,2
51142	CHCHD2	coiled-coil-helix-coiled-coil-helix domain containing 2	-1,2
55196	C12orf35	chromosome 12 open reading frame 35	-1,2
9790	BMS1	BMS1 homolog, ribosome assembly protein (yeast)	-1,2
303	ANXA2P1	annexin A2 pseudogene 1	-1,2
7324	UBE2E1	ubiquitin-conjugating enzyme E2E 1	-1,21
9806	SPOCK2	sparc/osteonectin, cwcv and kazal-like domains proteoglycan (testican) 2	-1,21
5876	RABGGTB	Rab geranylgeranyltransferase, beta subunit	-1,21

9782	MATR3	matrin 3	-1,21
3326	HSP90AB1	heat shock protein 90kDa alpha (cytosolic), class B member 1	-1,21
10236	HNRNPR	heterogeneous nuclear ribonucleoprotein R	-1,21
3187	HNRNPH1	heterogeneous nuclear ribonucleoprotein H1 (H)	-1,21
115362	GBP5	guanylate binding protein 5	-1,21
8087	FXR1	fragile X mental retardation, autosomal homolog 1	-1,21
116496	FAM129A	family with sequence similarity 129, member A	-1,21
10436	EMG1	EMG1 nucleolar protein homolog (S. cerevisiae)	-1,21
51726	DNAJB11	DnaJ (Hsp40) homolog, subfamily B, member 11	-1,21
221443	C6orf130	chromosome 6 open reading frame 130	-1,21
25939	ILMN_17752	Homo sapiens SAM domain and HD domain 1 (SAMHD1), mRNA.	-1,22
400156	ILMN_38819	PREDICTED: Homo sapiens 40S ribosomal protein S26-like (RPS26L), misc RNA.	-1,22
3927	LASP1	LIM and SH3 protein 1	-1,22
2949	GSTM5	glutathione S-transferase mu 5	-1,22
23495	TNFRSF13B	tumor necrosis factor receptor superfamily, member 13B	-1,23
6275	S100A4	S100 calcium binding protein A4	-1,23
5660	PSAP	prosaposin	-1,23
613037	LOC613037	nuclear pore complex interacting protein pseudogene	-1,23
	ILMN_82386	Homo sapiens cDNA FLJ32080 fis, clone OCBBF2000015	-1,23
170575	GIMAP1	GTPase, IMAP family member 1	-1,23
23401	FRAT2	frequently rearranged in advanced T-cell lymphomas 2	-1,23
8683	SRSF9	serine/arginine-rich splicing factor 9	-1,24
4831	ILMN_17934	Homo sapiens non-metastatic cells 2, protein (NM23B) expressed in (NME2), transcript variant 4, mRNA.	-1,24
4728	NDUFS8	NADH dehydrogenase (ubiquinone) Fe-S protein 8, 23kDa (NADH-coenzyme Q reductase)	-1,24
645018	ILMN_33646	PREDICTED: Homo sapiens similar to ribosomal protein S2 (LOC645018), mRNA.	-1,24
2996	ILMN_21264	Homo sapiens glycophorin E (GYPE), transcript variant 1, mRNA.	-1,24
1890	TYMP	thymidine phosphorylase	-1,24
5880	RAC2	ras-related C3 botulinum toxin substrate 2 (rho family, small GTP binding protein Rac2)	-1,25
2802	GOLGA3	golgin A3	-1,25
84678	KDM2B	lysine (K)-specific demethylase 2B	-1,25
51759	C9orf78	chromosome 9 open reading frame 78	-1,25
83989	FAM172A	family with sequence similarity 172, member A	-1,25
8673	VAMP8	vesicle-associated membrane protein 8 (endobrevin)	-1,26

388474	ILMN_39323	PREDICTED: Homo sapiens similar to 60S ribosomal protein L7a, transcript variant 3 (LOC388474), mRNA.	-1,26
3067	HDC	histidine decarboxylase	-1,26
841	CASP8	caspase 8, apoptosis-related cysteine peptidase	-1,26
6280	S100A9	S100 calcium binding protein A9	-1,27
8761	PABPC4	poly(A) binding protein, cytoplasmic 4 (inducible form)	-1,27
8338	HIST2H2AC	histone cluster 2, H2ac	-1,27
1629	ILMN_124019	Homo sapiens dihydrolipoamide branched chain transacylase E2 (DBT), nuclear gene encoding mitochondrial protein, mRNA.	-1,27
80335	WDR82	WD repeat domain 82	-1,28
92912	UBE2Q2	ubiquitin-conjugating enzyme E2Q family member 2	-1,28
8148	TAF15	TAF15 RNA polymerase II, TATA box binding protein (TBP)-associated factor, 68kDa	-1,28
22828	SCAF8	SR-related CTD-associated factor 8	-1,28
5621	PRNP	prion protein	-1,28
64976	MRPL40	mitochondrial ribosomal protein L40	-1,28
4245	MGAT1	mannosyl (alpha-1,3-)-glycoprotein beta-1,2-N-acetylglucosaminyltransferase	-1,28
	ILMN_114404	yu05d06.s1 Soares fetal liver spleen 1NFLS Homo sapiens cDNA clone IMAGE:232907 3, mRNA sequence	-1,28
2733	GLE1	GLE1 RNA export mediator homolog (yeast)	-1,28
89853	FAM125B	family with sequence similarity 125, member B	-1,28
834	CASP1	caspase 1, apoptosis-related cysteine peptidase (interleukin 1, beta, convertase)	-1,28
65980	BRD9	bromodomain containing 9	-1,28
199	AIF1	allograft inflammatory factor 1	-1,28
29855	UBN1	ubinuclein 1	-1,29
9045	ILMN_138835	Homo sapiens ribosomal protein L14 (RPL14), transcript variant 1, mRNA.	-1,29
9267	CYTH1	cytohesin 1	-1,29
26986	PABPC1	poly(A) binding protein, cytoplasmic 1	-1,29
441034	ILMN_31371	PREDICTED: Homo sapiens similar to 60S ribosomal protein L7a (LOC441034), mRNA.	-1,29
23258	DENND5A	DENN/MADD domain containing 5A	-1,29
9774	ILMN_41406	Homo sapiens BCL2-associated transcription factor 1 (BCLAF1), transcript variant 2, mRNA.	-1,29
26019	UPF2	UPF2 regulator of nonsense transcripts homolog (yeast)	-1,3
7311	UBA52	ubiquitin A-52 residue ribosomal protein fusion product 1	-1,3
5937	RBMS1	RNA binding motif, single stranded interacting protein 1	-1,3
389435	ILMN_30712	Homo sapiens hCG21078 (LOC389435), mRNA.	-1,3
55527	FEM1A	fem-1 homolog a (C. elegans)	-1,3

8667	EIF3H	eukaryotic translation initiation factor 3, subunit H	-1,3
10078	TSSC4	tumor suppressing subtransferable candidate 4	-1,31
22918	CD93	CD93 molecule	-1,31
10961	ERP29	endoplasmic reticulum protein 29	-1,31
29117	BRD7	bromodomain containing 7	-1,31
9550	ATP6V1G1	ATPase, H+ transporting, lysosomal 13kDa, V1 subunit G1	-1,31
217	ALDH2	aldehyde dehydrogenase 2 family (mitochondrial)	-1,31
11066	SNRNP35	small nuclear ribonucleoprotein 35kDa (U11/U12)	-1,32
140885	SIRPA	signal-regulatory protein alpha	-1,32
23198	PSME4	proteasome (prosome, macropain) activator subunit 4	-1,32
9360	PPIG	peptidylprolyl isomerase G (cyclophilin G)	-1,32
8566	PDXK	pyridoxal (pyridoxine, vitamin B6) kinase	-1,32
220988	HNRNPA3	heterogeneous nuclear ribonucleoprotein A3	-1,32
100008589	ILMN_1627	Homo sapiens 28S ribosomal RNA (LOC100008589), non-coding RNA.	-1,32
51665	ASB1	ankyrin repeat and SOCS box containing 1	-1,32
54543	TOMM7	translocase of outer mitochondrial membrane 7 homolog (yeast)	-1,33
5094	PCBP2	poly(rC) binding protein 2	-1,33
1476	CSTB	cystatin B (stefin B)	-1,33
1108	CHD4	chromodomain helicase DNA binding protein 4	-1,33
115416	C7orf30	chromosome 7 open reading frame 30	-1,33
9459	ARHGEF6	Rac/Cdc42 guanine nucleotide exchange factor (GEF) 6	-1,33
677	ZFP36L1	zinc finger protein 36, C3H type-like 1	-1,34
7416	VDAC1	voltage-dependent anion channel 1	-1,34
23029	RBM34	RNA binding motif protein 34	-1,34
8021	NUP214	nucleoporin 214kDa	-1,34
2483	FRG1	FSHD region gene 1	-1,34
819	CAMLG	calcium modulating ligand	-1,34
523	ATP6V1A	ATPase, H+ transporting, lysosomal 70kDa, V1 subunit A	-1,34
200030	ILMN_10536	Homo sapiens neuroblastoma breakpoint family, member 11-like (LOC200030), mRNA.	-1,35
120364	ILMN_35763	PREDICTED: Homo sapiens similar to heterogeneous nuclear ribonucleoprotein A1-like (LOC120364), mRNA.	-1,35
2113	ETS1	v-ets erythroblastosis virus E26 oncogene homolog 1 (avian)	-1,35
2000	ELF4	E74-like factor 4 (ets domain transcription factor)	-1,35
79180	EFHD2	EF-hand domain family, member D2	-1,35

54915	YTHDF1	YTH domain family, member 1	-1,36
57590	WDFY1	WD repeat and FYVE domain containing 1	-1,36
51669	TMEM66	transmembrane protein 66	-1,36
23753	SDF2L1	stromal cell-derived factor 2-like 1	-1,36
23568	ARL2BP	ADP-ribosylation factor-like 2 binding protein	-1,36
4938	OAS1	2'-5'-oligoadenylate synthetase 1, 40/46kDa	-1,37
728643	ILMN_183126	Homo sapiens heterogeneous nuclear ribonucleoprotein A1 pseudogene (LOC728643), non-coding RNA.	-1,37
649946	RPL23AP64	ribosomal protein L23a pseudogene 64	-1,37
648659	ILMN_31696	PREDICTED: Homo sapiens similar to ribosomal protein S3a, transcript variant 4 (LOC648659), mRNA.	-1,37
644131	CCT8P1	chaperonin containing TCP1, subunit 8 (theta) pseudogene 1	-1,37
642817	ILMN_46700	PREDICTED: Homo sapiens hypothetical LOC642817 (LOC642817), mRNA.	-1,37
158345	ILMN_36732	PREDICTED: Homo sapiens similar to ribosomal protein L4 (LOC158345), mRNA.	-1,37
3181	HNRNPA2B1	heterogeneous nuclear ribonucleoprotein A2/B1	-1,37
29799	YPEL1	yippee-like 1 (Drosophila)	-1,38
6210	RPS15A	ribosomal protein S15a	-1,38
118433	ILMN_3185	Homo sapiens ribosomal protein L23a pseudogene 7 (RPL23AP7) on chromosome 2.	-1,38
64981	MRPL34	mitochondrial ribosomal protein L34	-1,38
22887	FOXJ3	forkhead box J3	-1,38
506	ATP5B	ATP synthase, H+ transporting, mitochondrial F1 complex, beta polypeptide	-1,38
25801	GCA	grancalcin, EF-hand calcium binding protein	-1,39
55602	CDKN2AIP	CDKN2A interacting protein	-1,39
22980	TCF25	transcription factor 25 (basic helix-loop-helix)	-1,4
90007	MIDN	midnolin	-1,4
645688	ILMN_35293	PREDICTED: Homo sapiens similar to 60S ribosomal protein L12 (LOC645688), mRNA.	-1,4
10261	IGSF6	immunoglobulin superfamily, member 6	-1,4
3148	HMGB2	high mobility group box 2	-1,4
3301	DNAJA1	DnaJ (Hsp40) homolog, subfamily A, member 1	-1,4
10212	DDX39A	DEAD (Asp-Glu-Ala-Asp) box polypeptide 39A	-1,4
6281	S100A10	S100 calcium binding protein A10	-1,41
7879	RAB7A	RAB7A, member RAS oncogene family	-1,41
4666	NACA	nascent polypeptide-associated complex alpha subunit	-1,41
1915	EEF1A1	eukaryotic translation elongation factor 1 alpha 1	-1,41
121274	ZNF641	zinc finger protein 641	-1,42

8635	RNASET2	ribonuclease T2	-1,42
5204	PFDN5	prefoldin subunit 5	-1,42
4046	LSP1	lymphocyte-specific protein 1	-1,42
23117	ILMN_44181	PREDICTED: Homo sapiens KIAA0220-like protein, transcript variant 16 (LOC23117), mRNA.	-1,42
3128	HLA-DRB6	major histocompatibility complex, class II, DR beta 6 (pseudogene)	-1,42
1973	EIF4A1	eukaryotic translation initiation factor 4A1	-1,42
11033	ADAP1	ArfGAP with dual PH domains 1	-1,42
81556	C15orf44	chromosome 15 open reading frame 44	-1,42
7705	ZNF146	zinc finger protein 146	-1,43
7532	YWHAG	tyrosine 3-monooxygenase/tryptophan 5-monooxygenase activation protein, gamma polypeptide	-1,43
9349	RPL23	ribosomal protein L23	-1,43
5788	PTPRC	protein tyrosine phosphatase, receptor type, C	-1,43
650518	ILMN_39973	PREDICTED: Homo sapiens similar to Proteasome subunit alpha type 6 (Proteasome iota chain) (Macropain iota chain) (Multicatalytic endopeptidase complex iota chain) (LOC650518), mRNA.	-1,43
	ILMN_98835	dq02f02.y1 NIH_MGC_2 Homo sapiens cDNA clone IMAGE:2846715 3, mRNA sequence	-1,43
54504	CPVL	carboxypeptidase, vitellogenic-like	-1,43
5859	QARS	glutaminyl-tRNA synthetase	-1,44
401992	ILMN_138791	PREDICTED: Homo sapiens olfactory receptor, family 2, subfamily T, member 2 (OR2T2), mRNA.	-1,44
4818	NKG7	natural killer cell group 7 sequence	-1,44
653232	ILMN_41130	PREDICTED: Homo sapiens hypothetical LOC653232, transcript variant 4 (LOC653232), mRNA.	-1,44
389137	LOC389137	armadillo repeat containing 10 pseudogene	-1,44
3939	LDHA	lactate dehydrogenase A	-1,44
3126	ILMN_18172	Homo sapiens major histocompatibility complex, class II, DR beta 4 (HLA-DRB4), mRNA.	-1,44
10961	ERP29	endoplasmic reticulum protein 29	-1,44
7327	UBE2G2	ubiquitin-conjugating enzyme E2G 2	-1,45
6595	SMARCA2	SWI/SNF related, matrix associated, actin dependent regulator of chromatin, subfamily a, member 2	-1,45
22934	RPIA	ribose 5-phosphate isomerase A	-1,45
79717	ILMN_25951	Homo sapiens phosphopantothenoylcysteine synthetase (PPCS), transcript variant 1, mRNA.	-1,45
651149	ILMN_36491	PREDICTED: Homo sapiens similar to 60S ribosomal protein L3 (L4) (LOC651149), mRNA.	-1,46
642250	ILMN_44960	Homo sapiens hCG39912 (LOC642250), mRNA.	-1,46
3310	HSPA6	heat shock 70kDa protein 6 (HSP70B')	-1,46
120425	AMICA1	adhesion molecule, interacts with CXADR antigen 1	-1,46
6234	RPS28	ribosomal protein S28	-1,47

10578	GNLY	granulysin	-1,47
9337	CNOT8	CCR4-NOT transcription complex, subunit 8	-1,47
6136	RPL12	ribosomal protein L12	-1,47
694	BTG1	B-cell translocation gene 1, anti-proliferative	-1,47
308	ANXA5	annexin A5	-1,47
91746	YTHDC1	YTH domain containing 1	-1,48
9589	WTAP	Wilms tumor 1 associated protein	-1,48
25777	SUN2	Sad1 and UNC84 domain containing 2	-1,48
57665	RDH14	retinol dehydrogenase 14 (all-trans/9-cis/11-cis)	-1,48
5898	RALA	v-ral simian leukemia viral oncogene homolog A (ras related)	-1,48
23420	ILMN_1736	Homo sapiens NODAL modulator 1 (NOMO1), mRNA.	-1,48
8837	ILMN_4321	Homo sapiens CASP8 and FADD-like apoptosis regulator (CFLAR), mRNA.	-1,48
28958	CCDC56	coiled-coil domain containing 56	-1,48
7919	DDX39B	DEAD (Asp-Glu-Ala-Asp) box polypeptide 39B	-1,48
8682	PEA15	phosphoprotein enriched in astrocytes 15	-1,49
6205	RPS11	ribosomal protein S11	-1,49
10608	MXD4	MAX dimerization protein 4	-1,5
4048	LTA4H	leukotriene A4 hydrolase	-1,5
5788	PTPRC	protein tyrosine phosphatase, receptor type, C	-1,51
146722	ILMN_4334	Homo sapiens CD300 molecule-like family member f (CD300LF), mRNA.	-1,51
7175	TPR	translocated promoter region (to activated MET oncogene)	-1,52
6625	SNRNP70	small nuclear ribonucleoprotein 70kDa (U1)	-1,52
27303	RBMS3	RNA binding motif, single stranded interacting protein 3	-1,52
9045	RPL14	ribosomal protein L14	-1,52
2971	GTF3A	general transcription factor IIIA	-1,52
1387	CREBBP	CREB binding protein	-1,52
4706	NDUFAB1	NADH dehydrogenase (ubiquinone) 1, alpha/beta subcomplex, 1, 8kDa	-1,53
1642	DDB1	damage-specific DNA binding protein 1, 127kDa	-1,53
8725	C19orf2	chromosome 19 open reading frame 2	-1,53
3001	GZMA	granzyme A (granzyme 1, cytotoxic T-lymphocyte-associated serine esterase 3)	-1,54
80777	CYB5B	cytochrome b5 type B (outer mitochondrial membrane)	-1,54
6204	RPS10	ribosomal protein S10	-1,55
9867	PJA2	praja ring finger 2	-1,55

57819	LSM2	LSM2 homolog, U6 small nuclear RNA associated (S. cerevisiae)	-1,55
10581	IFITM2	interferon induced transmembrane protein 2 (1-8D)	-1,55
389674	ILMN_38391	PREDICTED: Homo sapiens heterogeneous nuclear ribonucleoprotein A1 pseudogene 4 (HNRPA1P4), mRNA.	-1,55
2734	GLG1	golgi glycoprotein 1	-1,55
4736	RPL10A	ribosomal protein L10a	-1,56
5694	PSMB6	proteasome (prosome, macropain) subunit, beta type, 6	-1,56
64397	ZFP106	zinc finger protein 106 homolog (mouse)	-1,57
7305	TYROBP	TYRO protein tyrosine kinase binding protein	-1,57
6229	RPS24	ribosomal protein S24	-1,57
6128	RPL6	ribosomal protein L6	-1,57
6228	RPS23	ribosomal protein S23	-1,57
653232	ILMN_31434	PREDICTED: Homo sapiens hypothetical LOC653232, transcript variant 4 (LOC653232), mRNA.	-1,57
829	CAPZA1	capping protein (actin filament) muscle Z-line, alpha 1	-1,57
94104	GCFC1	GC-rich sequence DNA-binding factor 1	-1,57
11224	ILMN_11814	Homo sapiens ribosomal protein L35 (RPL35), mRNA.	-1,58
4716	NDUFB10	NADH dehydrogenase (ubiquinone) 1 beta subcomplex, 10, 22kDa	-1,58
23048	FNBP1	formin binding protein 1	-1,58
1968	EIF2S3	eukaryotic translation initiation factor 2, subunit 3 gamma, 52kDa	-1,58
26135	SERBP1	SERPINE1 mRNA binding protein 1	-1,59
5687	PSMA6	proteasome (prosome, macropain) subunit, alpha type, 6	-1,59
388275	ILMN_39089	PREDICTED: Homo sapiens similar to Heterogeneous nuclear ribonucleoprotein A1 (Helix-destabilizing protein) (Single-strand binding protein) (hnRNP core protein A1) (HDP-1) (Topoisomerase-inhibitor suppressed) (LOC388275), mRNA.	-1,59
6426	SRSF1	serine/arginine-rich splicing factor 1	-1,6
6205	RPS11	ribosomal protein S11	-1,6
650646	ILMN_46687	PREDICTED: Homo sapiens similar to 40S ribosomal protein S26 (LOC650646), mRNA.	-1,6
3295	HSD17B4	hydroxysteroid (17-beta) dehydrogenase 4	-1,6
7555	CNBP	CCHC-type zinc finger, nucleic acid binding protein	-1,6
29121	ILMN_138738	Homo sapiens C-type lectin domain family 2, member D (CLEC2D), transcript variant 1, mRNA.	-1,6
960	CD44	CD44 molecule (Indian blood group)	-1,6
90416	C15orf57	chromosome 15 open reading frame 57	-1,6
5265	SERPINA1	serpin peptidase inhibitor, clade A (alpha-1 antiproteinase, antitrypsin), member 1	-1,61
6217	RPS16	ribosomal protein S16	-1,61

6169	RPL38	ribosomal protein L38	-1,61
11158	RABL2B	RAB, member of RAS oncogene family-like 2B	-1,61
646350	ILMN_39634	PREDICTED: Homo sapiens similar to 60S ribosomal protein L39 (LOC646350), mRNA.	-1,61
2212	FCGR2A	Fc fragment of IgG, low affinity IIa, receptor (CD32)	-1,61
55729	ATF7IP	activating transcription factor 7 interacting protein	-1,61
328	APEX1	APEX nuclease (multifunctional DNA repair enzyme) 1	-1,61
6124	ILMN_21554	Homo sapiens ribosomal protein L4 (RPL4), mRNA.	-1,62
10181	RBM5	RNA binding motif protein 5	-1,62
441032	ILMN_46443	PREDICTED: Homo sapiens similar to eukaryotic translation elongation factor 1 alpha 1, transcript variant 11 (LOC441032), mRNA.	-1,62
155038	ILMN_6869	Homo sapiens GTPase, IMAP family member 8 (GIMAP8), mRNA.	-1,62
50854	C6orf48	chromosome 6 open reading frame 48	-1,62
6128	RPL6	ribosomal protein L6	-1,63
10250	SRRM1	serine/arginine repetitive matrix 1	-1,64
3184	HNRNPD	heterogeneous nuclear ribonucleoprotein D (AU-rich element RNA binding protein 1, 37kDa)	-1,64
81929	SEH1L	SEH1-like (S. cerevisiae)	-1,65
6230	RPS25	ribosomal protein S25	-1,65
23484	LEPROTL1	leptin receptor overlapping transcript-like 1	-1,65
54518	APBB1IP	amyloid beta (A4) precursor protein-binding, family B, member 1 interacting protein	-1,65
6224	RPS20	ribosomal protein S20	-1,66
6139	RPL17	ribosomal protein L17	-1,66
51667	NUB1	negative regulator of ubiquitin-like proteins 1	-1,66
4050	LTB	lymphotoxin beta (TNF superfamily, member 3)	-1,66
2268	FGR	Gardner-Rasheed feline sarcoma viral (v-fgr) oncogene homolog	-1,66
64411	ARAP3	ArfGAP with RhoGAP domain, ankyrin repeat and PH domain 3	-1,66
6235	RPS29	ribosomal protein S29	-1,67
57162	PELI1	pellino homolog 1 (Drosophila)	-1,67
606724	ILMN_9207	Homo sapiens coronin, actin binding protein, 1A pseudogene (LOC606724), non-coding RNA.	-1,67
6222	RPS18	ribosomal protein S18	-1,68
3071	NCKAP1L	NCK-associated protein 1-like	-1,68
664709	HNRNPA1P10	heterogeneous nuclear ribonucleoprotein A1 pseudogene 10	-1,68
81576	CCDC130	coiled-coil domain containing 130	-1,68
390354	ILMN_138133	PREDICTED: Homo sapiens similar to ribosomal protein L18a; 60S ribosomal protein L18a, transcript variant 36	-1,69

		(LOC390354), misc RNA.	
1938	EEF2	eukaryotic translation elongation factor 2	-1,69
64333	ARHGAP9	Rho GTPase activating protein 9	-1,69
10628	TXNIP	thioredoxin interacting protein	-1,7
9984	THOC1	THO complex 1	-1,7
4809	NHP2L1	NHP2 non-histone chromosome protein 2-like 1 (S. cerevisiae)	-1,7
8751	ADAM15	ADAM metallopeptidase domain 15	-1,7
7431	VIM	vimentin	-1,71
400156	ILMN_27668	Homo sapiens 40S ribosomal protein S26-like (RPS26L), non-coding RNA.	-1,71
5547	PRCP	prolylcarboxypeptidase (angiotensinase C)	-1,72
136143	ILMN_32715	PREDICTED: Homo sapiens similar to ribosomal protein L18 (LOC136143), mRNA.	-1,72
10399	GNB2L1	guanine nucleotide binding protein (G protein), beta polypeptide 2-like 1	-1,72
64771	C6orf106	chromosome 6 open reading frame 106	-1,72
27013	CNPPD1	cyclin Pas1/PHO80 domain containing 1	-1,72
7538	ZFP36	zinc finger protein 36, C3H type, homolog (mouse)	-1,73
6194	RPS6	ribosomal protein S6	-1,73
6230	RPS25	ribosomal protein S25	-1,73
168537	GIMAP7	GTPase, IMAP family member 7	-1,73
5435	POLR2F	polymerase (RNA) II (DNA directed) polypeptide F	-1,74
6778	STAT6	signal transducer and activator of transcription 6, interleukin-4 induced	-1,75
64744	SMAP2	small ArfGAP2	-1,75
6232	RPS27	ribosomal protein S27	-1,75
80223	RAB11FIP1	RAB11 family interacting protein 1 (class I)	-1,75
648343	ILMN_42525	PREDICTED: Homo sapiens similar to protein phosphatase 1 regulatory subunit 14B (LOC648343), mRNA.	-1,75
3183	ILMN_24356	Homo sapiens heterogeneous nuclear ribonucleoprotein C (C1/C2) (HNRPC), transcript variant 1, mRNA.	-1,75
746	C11orf10	chromosome 11 open reading frame 10	-1,75
650298	ILMN_42423	PREDICTED: Homo sapiens similar to 40S ribosomal protein S26 (LOC650298), mRNA.	-1,76
8721	EDF1	endothelial differentiation-related factor 1	-1,76
10694	CCT8	chaperonin containing TCP1, subunit 8 (theta)	-1,76
6168	RPL37A	ribosomal protein L37a	-1,77
644992	ILMN_33996	PREDICTED: Homo sapiens hypothetical LOC644992 (LOC644992), mRNA.	-1,77
144983	HNRNPA1L2	heterogeneous nuclear ribonucleoprotein A1-like 2	-1,77
23180	RFTN1	raftlin, lipid raft linker 1	-1,78

7940	LST1	leukocyte specific transcript 1	-1,78
6230	RPS25	ribosomal protein S25	-1,78
221092	HNRNPUL2	heterogeneous nuclear ribonucleoprotein U-like 2	-1,78
3128	HLA-DRB6	major histocompatibility complex, class II, DR beta 6 (pseudogene)	-1,78
79081	C11orf48	chromosome 11 open reading frame 48	-1,78
23478	SEC11A	SEC11 homolog A (S. cerevisiae)	-1,79
6132	RPL8	ribosomal protein L8	-1,79
6234	RPS28	ribosomal protein S28	-1,79
1958	EGR1	early growth response 1	-1,79
11159	RABL2A	RAB, member of RAS oncogene family-like 2A	-1,8
5720	PSME1	proteasome (prosome, macropain) activator subunit 1 (PA28 alpha)	-1,8
6128	RPL6	ribosomal protein L6	-1,8
3125	HLA-DRB3	major histocompatibility complex, class II, DR beta 3	-1,8
8364	HIST1H4C	histone cluster 1, H4c	-1,8
3268	AGFG2	ArfGAP with FG repeats 2	-1,8
6129	RPL7	ribosomal protein L7	-1,81
3303	HSPA1A	heat shock 70kDa protein 1A	-1,81
521	ATP5I	ATP synthase, H+ transporting, mitochondrial Fo complex, subunit E	-1,81
6741	SSB	Sjogren syndrome antigen B (autoantigen La)	-1,82
6688	SPI1	spleen focus forming virus (SFFV) proviral integration oncogene spi1	-1,82
25832	NBPF14	neuroblastoma breakpoint family, member 14	-1,82
284821	ILMN_43560	PREDICTED: Homo sapiens similar to ribosomal protein L13a (LOC284821), mRNA.	-1,82
2214	ILMN_181791	Homo sapiens Fc fragment of IgG, low affinity IIIa, receptor (CD16a) (FCGR3A), transcript variant 1, mRNA.	-1,82
10922	FASTK	Fas-activated serine/threonine kinase	-1,82
151636	ILMN_14733	Homo sapiens deltex 3-like (Drosophila) (DTX3L), mRNA.	-1,82
64781	CERK	ceramide kinase	-1,82
6430	SRSF5	serine/arginine-rich splicing factor 5	-1,83
6141	RPL18	ribosomal protein L18	-1,83
23405	DICER1	dicer 1, ribonuclease type III	-1,83
402694	ILMN_32451	Homo sapiens similar to ribosomal protein L15 (LOC402694), mRNA.	-1,83
2319	FLOT2	flotillin 2	-1,83
54543	TOMM7	translocase of outer mitochondrial membrane 7 homolog (yeast)	-1,84
6637	ILMN_30298	Homo sapiens small nuclear ribonucleoprotein polypeptide G (SNRPG), mRNA.	-1,84

6633	SNRPD2	small nuclear ribonucleoprotein D2 polypeptide 16.5kDa	-1,84
6427	SRSF2	serine/arginine-rich splicing factor 2	-1,84
730316	ILMN_28309	PREDICTED: Homo sapiens similar to Nuclear envelope pore membrane protein POM 121 (Pore membrane protein of 121 kDa) (P145) (LOC730316), mRNA.	-1,84
643949	ILMN_35406	PREDICTED: Homo sapiens similar to 60S acidic ribosomal protein P2 (LOC643949), mRNA.	-1,84
643284	ILMN_40380	PREDICTED: Homo sapiens similar to 40S ribosomal protein S29 (LOC643284), mRNA.	-1,84
84619	ZGPAT	zinc finger, CCCH-type with G patch domain	-1,85
6231	RPS26	ribosomal protein S26	-1,85
5204	PFDN5	prefoldin subunit 5	-1,85
8665	EIF3F	eukaryotic translation initiation factor 3, subunit F	-1,85
1654	DDX3X	DEAD (Asp-Glu-Ala-Asp) box polypeptide 3, X-linked	-1,85
5073	PARN	poly(A)-specific ribonuclease	-1,86
649548	ILMN_31442	PREDICTED: Homo sapiens similar to 60S ribosomal protein L32 (LOC649548), mRNA.	-1,86
440396	ILMN_45411	Homo sapiens similar to Heterogeneous nuclear ribonucleoprotein A1 (Helix-destabilizing protein) (Single-strand binding protein) (hnRNP core protein A1) (HDP-1) (Topoisomerase-inhibitor suppressed) (LOC440396), non-coding RNA.	-1,86
221960	C7orf28B	chromosome 7 open reading frame 28B	-1,86
9168	TMSB10	thymosin beta 10	-1,87
29101	SSU72	SSU72 RNA polymerase II CTD phosphatase homolog (S. cerevisiae)	-1,87
51187	RSL24D1	ribosomal L24 domain containing 1	-1,87
6176	RPLP1	ribosomal protein, large, P1	-1,87
6169	RPL38	ribosomal protein L38	-1,87
5265	SERPINA1	serpin peptidase inhibitor, clade A (alpha-1 antiproteinase, antitrypsin), member 1	-1,88
6235	RPS29	ribosomal protein S29	-1,88
645	BLVRB	biliverdin reductase B (flavin reductase (NADPH))	-1,88
54440	SASH3	SAM and SH3 domain containing 3	-1,89
	ILMN_111893	full-length cDNA clone CS0DI056YK21 of Placenta Cot 25-normalized of Homo sapiens (human)	-1,89
29924	EPN1	epsin 1	-1,89
5018	OXA1L	oxidase (cytochrome c) assembly 1-like	-1,9
7940	LST1	leukocyte specific transcript 1	-1,91
648622	ILMN_42936	PREDICTED: Homo sapiens similar to ribosomal protein S27 (LOC648622), mRNA.	-1,91
389672	ILMN_35589	PREDICTED: Homo sapiens similar to 40S ribosomal protein SA (p40) (34/67 kDa laminin receptor) (Colon carcinoma laminin-binding protein) (NEM/1CHD4) (Multidrug resistance-associated protein MGr1-Ag), transcript variant 3	-1,91

		(LOC389672), mRNA.	
23163	GGA3	golgi-associated, gamma adaptin ear containing, ARF binding protein 3	-1,91
1666	DECR1	2,4-dienoyl CoA reductase 1, mitochondrial	-1,91
7454	WAS	Wiskott-Aldrich syndrome (eczema-thrombocytopenia)	-1,92
7430	EZR	ezrin	-1,92
6232	RPS27	ribosomal protein S27	-1,93
4691	NCL	nucleolin	-1,93
146053	ILMN_40640	PREDICTED: Homo sapiens similar to ribosomal protein S3a, transcript variant 4 (LOC146053), mRNA.	-1,93
56910	STARD7	StAR-related lipid transfer (START) domain containing 7	-1,94
5547	PRCP	prolylcarboxypeptidase (angiotensinase C)	-1,94
3312	HSPA8	heat shock 70kDa protein 8	-1,94
3320	HSP90AA1	heat shock protein 90kDa alpha (cytosolic), class A member 1	-1,94
1915	EEF1A1	eukaryotic translation elongation factor 1 alpha 1	-1,94
387066	ILMN_38384	PREDICTED: Homo sapiens chromosome 6 open reading frame 160, transcript variant 4 (C6orf160), mRNA.	-1,94
4708	NDUFB2	NADH dehydrogenase (ubiquinone) 1 beta subcomplex, 2, 8kDa	-1,95
651202	ILMN_45225	PREDICTED: Homo sapiens similar to large subunit ribosomal protein L36a (LOC651202), mRNA.	-1,95
3123	HLA-DRB1	major histocompatibility complex, class II, DR beta 1	-1,95
64333	ARHGAP9	Rho GTPase activating protein 9	-1,95
51234	TMEM85	transmembrane protein 85	-1,96
402251	ILMN_45342	PREDICTED: Homo sapiens similar to eukaryotic translation elongation factor 1 alpha 2 (LOC402251), mRNA.	-1,96
6161	RPL32	ribosomal protein L32	-1,97
56655	POLE4	polymerase (DNA-directed), epsilon 4 (p12 subunit)	-1,97
651894	ILMN_34885	PREDICTED: Homo sapiens similar to ribosomal protein S12 (LOC651894), mRNA.	-1,97
645899	ILMN_42597	PREDICTED: Homo sapiens similar to 40S ribosomal protein S28 (LOC645899), mRNA.	-1,97
168537	GIMAP7	GTPase, IMAP family member 7	-1,97
6046	BRD2	bromodomain containing 2	-1,97
3385	ICAM3	intercellular adhesion molecule 3	-1,98
6136	RPL12	ribosomal protein L12	-1,99
10424	PGRMC2	progesterone receptor membrane component 2	-1,99
644604	EEF1A1P12	eukaryotic translation elongation factor 1 alpha 1 pseudogene 12	-1,99
	ILMN_72502	Homo sapiens cDNA FLJ31407 fis, clone NT2NE2000137	-1,99
3117	ILMN_41173	PREDICTED: Homo sapiens major histocompatibility complex, class II, DQ alpha 1, transcript variant 10 (HLA-DQA1), mRNA.	-1,99

23167	EFR3A	EFR3 homolog A (S. cerevisiae)	-1,99
1915	EEF1A1	eukaryotic translation elongation factor 1 alpha 1	-1,99
1350	COX7C	cytochrome c oxidase subunit VIIc	-1,99
9184	BUB3	budding uninhibited by benzimidazoles 3 homolog (yeast)	-1,99
6193	RPS5	ribosomal protein S5	-2
128346	C1orf162	chromosome 1 open reading frame 162	-2,01
6279	S100A8	S100 calcium binding protein A8	-2,02
7297	TYK2	tyrosine kinase 2	-2,03
6206	RPS12	ribosomal protein S12	-2,03
7979	SHFM1	split hand/foot malformation (ectrodactyly) type 1	-2,04
6235	RPS29	ribosomal protein S29	-2,04
9219	MTA2	metastasis associated 1 family, member 2	-2,04
647361	ILMN_37525	PREDICTED: Homo sapiens similar to 40S ribosomal protein S29 (LOC647361), mRNA.	-2,04
10437	IFI30	interferon, gamma-inducible protein 30	-2,04
1716	DGUOK	deoxyguanosine kinase	-2,04
648210	ILMN_40930	PREDICTED: Homo sapiens similar to Heterogeneous nuclear ribonucleoprotein A1 (Helix-destabilizing protein) (Single-strand RNA-binding protein) (hnRNP core protein A1) (HDP) (LOC648210), mRNA.	-2,05
6128	RPL6	ribosomal protein L6	-2,05
23019	CNOT1	CCR4-NOT transcription complex, subunit 1	-2,05
6194	RPS6	ribosomal protein S6	-2,06
6188	RPS3	ribosomal protein S3	-2,06
645737	ILMN_44612	PREDICTED: Homo sapiens similar to 60S ribosomal protein L7, transcript variant 1 (LOC645737), mRNA.	-2,06
645683	RPL13AP3	ribosomal protein L13a pseudogene 3	-2,06
10983	CCNI	cyclin I	-2,06
6741	SSB	Sjogren syndrome antigen B (autoantigen La)	-2,07
6256	RXRA	retinoid X receptor, alpha	-2,07
22827	PUF60	poly-U binding splicing factor 60KDa	-2,08
6159	RPL29	ribosomal protein L29	-2,08
440733	ILMN_38430	PREDICTED: Homo sapiens similar to 40S ribosomal protein S15 (RIG protein) (LOC440733), mRNA.	-2,08
	ILMN_102288	Human mRNA for T-cell specific protein	-2,08
9991	ROD1	ROD1 regulator of differentiation 1 (S. pombe)	-2,09
284058	KIAA1267	KIAA1267	-2,09
201895	C4orf34	chromosome 4 open reading frame 34	-2,1

645436	ILMN_31983	PREDICTED: Homo sapiens similar to Heterogeneous nuclear ribonucleoprotein A1 (Helix-destabilizing protein) (Single-strand binding protein) (hnRNP core protein A1) (HDP-1) (Topoisomerase-inhibitor suppressed) (LOC645436), mRNA.	-2,11
6129	RPL7	ribosomal protein L7	-2,11
402057	ILMN_181541	Homo sapiens similar to 40S ribosomal protein S17 (LOC402057), mRNA.	-2,11
401019	ILMN_35511	PREDICTED: Homo sapiens similar to 40S ribosomal protein S15 (RIG protein), transcript variant 3 (LOC401019), mRNA.	-2,11
1615	DARS	aspartyl-tRNA synthetase	-2,11
6235	RPS29	ribosomal protein S29	-2,12
6125	RPL5	ribosomal protein L5	-2,12
9516	LITAF	lipopolysaccharide-induced TNF factor	-2,12
10521	DDX17	DEAD (Asp-Glu-Ala-Asp) box polypeptide 17	-2,12
84881	RPUSD4	RNA pseudouridylate synthase domain containing 4	-2,13
6191	RPS4X	ribosomal protein S4, X-linked	-2,13
6176	RPLP1	ribosomal protein, large, P1	-2,13
441124	ILMN_8311	PREDICTED: Homo sapiens hypothetical LOC441124 (LOC441124), mRNA.	-2,13
6134	RPL10	ribosomal protein L10	-2,15
6191	RPS4X	ribosomal protein S4, X-linked	-2,16
5920	RARRES3	retinoic acid receptor responder (tazarotene induced) 3	-2,16
1975	EIF4B	eukaryotic translation initiation factor 4B	-2,16
7555	CNBP	CCHC-type zinc finger, nucleic acid binding protein	-2,16
83880	ILMN_9478	Homo sapiens IFP38 (IFP38), mRNA.	-2,17
3431	SP110	SP110 nuclear body protein	-2,18
6223	RPS19	ribosomal protein S19	-2,18
6122	RPL3	ribosomal protein L3	-2,18
2799	GNS	glucosamine (N-acetyl)-6-sulfatase	-2,18
552891	ILMN_29106	Homo sapiens DNAJC25-GNG10 readthrough (DNAJC25-GNG10), mRNA.	-2,18
6218	RPS17	ribosomal protein S17	-2,19
6159	RPL29	ribosomal protein L29	-2,19
390876	ILMN_35719	PREDICTED: Homo sapiens similar to 60S ribosomal protein L35 (LOC390876), mRNA.	-2,19
388524	ILMN_27037	Homo sapiens similar to Laminin receptor 1 (LOC388524), mRNA.	-2,2
6134	RPL10	ribosomal protein L10	-2,2
648000	ILMN_32447	PREDICTED: Homo sapiens similar to 60S ribosomal protein L7, transcript variant 1 (LOC648000), mRNA.	-2,22

3312	HSPA8	heat shock 70kDa protein 8	-2,22
994	CDC25B	cell division cycle 25 homolog B (S. pombe)	-2,22
6152	RPL24	ribosomal protein L24	-2,23
7157	ILMN_11285	Homo sapiens tumor protein p53 (TP53), mRNA.	-2,24
1937	EEF1G	eukaryotic translation elongation factor 1 gamma	-2,24
6146	RPL22	ribosomal protein L22	-2,24
10657	KHDRBS1	KH domain containing, RNA binding, signal transduction associated 1	-2,24
6208	RPS14	ribosomal protein S14	-2,25
391656	ILMN_43142	PREDICTED: Homo sapiens similar to hCG1640454 (LOC391656), mRNA.	-2,26
1933	EEF1B2	eukaryotic translation elongation factor 1 beta 2	-2,26
55748	CNDP2	CNDP dipeptidase 2 (metallopeptidase M20 family)	-2,26
4688	ILMN_8146	Homo sapiens neutrophil cytosolic factor 2 (65kDa, chronic granulomatous disease, autosomal 2) (NCF2), mRNA.	-2,27
6152	RPL24	ribosomal protein L24	-2,27
440737	ILMN_39347	PREDICTED: Homo sapiens similar to 60S ribosomal protein L35 (LOC440737), mRNA.	-2,27
388122	ILMN_37996	PREDICTED: Homo sapiens hypothetical LOC388122 (LOC388122), mRNA.	-2,27
27335	EIF3K	eukaryotic translation initiation factor 3, subunit K	-2,27
64115	ILMN_174163	Homo sapiens chromosome 10 open reading frame 54 (C10orf54), mRNA.	-2,27
6187	RPS2	ribosomal protein S2	-2,28
1936	EEF1D	eukaryotic translation elongation factor 1 delta (guanine nucleotide exchange protein)	-2,28
23589	CARHSP1	calcium regulated heat stable protein 1, 24kDa	-2,28
303	ANXA2P1	annexin A2 pseudogene 1	-2,28
27430	ILMN_18923	Homo sapiens methionine adenosyltransferase II, beta (MAT2B), transcript variant 1, mRNA.	-2,29
56616	DIABLO	diablo, IAP-binding mitochondrial protein	-2,29
2091	FBL	fibrillarin	-2,3
6189	RPS3A	ribosomal protein S3A	-2,31
51371	POMP	proteasome maturation protein	-2,31
6129	RPL7	ribosomal protein L7	-2,31
644037	HNRNPA1P12	heterogeneous nuclear ribonucleoprotein A1 pseudogene 12	-2,31
10288	ILMN_180674	Homo sapiens leukocyte immunoglobulin-like receptor, subfamily B (with TM and ITIM domains), member 2 (LILRB2), transcript variant 2, mRNA.	-2,31
81606	LBH	limb bud and heart development homolog (mouse)	-2,31
3420	IDH3B	isocitrate dehydrogenase 3 (NAD+) beta	-2,31
23480	SEC61G	Sec61 gamma subunit	-2,32

6166	RPL36AL	ribosomal protein L36a-like	-2,32
6165	RPL35A	ribosomal protein L35a	-2,32
10724	ILMN_11399	Homo sapiens meningioma expressed antigen 5 (hyaluronidase) (MGEA5), mRNA.	-2,32
6166	RPL36AL	ribosomal protein L36a-like	-2,34
653773	ILMN_44662	PREDICTED: Homo sapiens similar to ribosomal protein L31 (LOC653773), mRNA.	-2,34
346950	ILMN_36919	PREDICTED: Homo sapiens similar to ribosomal protein L37 (LOC346950), mRNA.	-2,34
9750	ILMN_21545	Homo sapiens family with sequence similarity 65, member B (FAM65B), transcript variant 2, mRNA.	-2,34
3646	EIF3E	eukaryotic translation initiation factor 3, subunit E	-2,35
8906	AP1G2	adaptor-related protein complex 1, gamma 2 subunit	-2,35
11100	HNRNPUL1	heterogeneous nuclear ribonucleoprotein U-like 1	-2,36
6142	RPL18A	ribosomal protein L18a	-2,39
441246	ILMN_31934	PREDICTED: Homo sapiens similar to 60S ribosomal protein L35, transcript variant 1 (LOC441246), mRNA.	-2,39
4170	MCL1	myeloid cell leukemia sequence 1 (BCL2-related)	-2,4
441377	ILMN_31681	PREDICTED: Homo sapiens similar to 40S ribosomal protein S26 (LOC441377), mRNA.	-2,4
6142	RPL18A	ribosomal protein L18a	-2,42
11224	RPL35	ribosomal protein L35	-2,42
3727	ILMN_29325	Homo sapiens jun D proto-oncogene (JUND), mRNA.	-2,42
6207	RPS13	ribosomal protein S13	-2,43
648249	ILMN_33635	PREDICTED: Homo sapiens similar to 40S ribosomal protein SA (p40) (34/67 kDa laminin receptor) (Colon carcinoma laminin-binding protein) (NEM/1CHD4) (Multidrug resistance-associated protein MGr1-Ag), transcript variant 3 (LOC648249), mRNA.	-2,43
3687	ILMN_7741	Homo sapiens integrin, alpha X (complement component 3 receptor 4 subunit) (ITGAX), mRNA.	-2,43
1933	EEF1B2	eukaryotic translation elongation factor 1 beta 2	-2,43
6133	RPL9	ribosomal protein L9	-2,44
6144	RPL21	ribosomal protein L21	-2,44
652624	ILMN_46806	PREDICTED: Homo sapiens similar to 40S ribosomal protein SA (p40) (34/67 kDa laminin receptor) (LOC652624), mRNA.	-2,44
644604	EEF1A1P12	eukaryotic translation elongation factor 1 alpha 1 pseudogene 12	-2,44
402057	ILMN_40648	Homo sapiens similar to 40S ribosomal protein S17 (LOC402057), mRNA.	-2,45
51316	PLAC8	placenta-specific 8	-2,46
142	ILMN_3056	Homo sapiens poly (ADP-ribose) polymerase family, member 1 (PARP1), mRNA.	-2,46
51335	NGRN	neugrin, neurite outgrowth associated	-2,47
728973	ILMN_38370	PREDICTED: Homo sapiens similar to 40S ribosomal protein S7 (S8) (LOC728973), mRNA.	-2,47

387867	ILMN_34982	PREDICTED: Homo sapiens similar to 40S ribosomal protein SA (p40) (34/67 kDa laminin receptor) (Colon carcinoma laminin-binding protein) (NEM/1CHD4) (Multidrug resistance-associated protein MGr1-Ag) (LOC387867), mRNA.	-2,47
2219	FCN1	ficolin (collagen/fibrinogen domain containing) 1	-2,47
647856	ILMN_42194	PREDICTED: Homo sapiens similar to 40S ribosomal protein SA (p40) (34/67 kDa laminin receptor) (Colon carcinoma laminin-binding protein) (NEM/1CHD4) (Multidrug resistance-associated protein MGr1-Ag) (LOC647856), mRNA.	-2,48
645385	ILMN_33113	PREDICTED: Homo sapiens similar to heterogeneous nuclear ribonucleoprotein A1 (LOC645385), mRNA.	-2,48
8721	EDF1	endothelial differentiation-related factor 1	-2,48
6139	RPL17	ribosomal protein L17	-2,49
81609	SNX27	sorting nexin family member 27	-2,5
1933	ILMN_4325	Homo sapiens eukaryotic translation elongation factor 1 beta 2 (EEF1B2), transcript variant 3, mRNA.	-2,5
50856	ILMN_8463	Homo sapiens C-type lectin domain family 4, member A (CLEC4A), transcript variant 4, mRNA.	-2,51
6142	RPL18A	ribosomal protein L18a	-2,52
5777	PTPN6	protein tyrosine phosphatase, non-receptor type 6	-2,52
9991	ROD1	ROD1 regulator of differentiation 1 (S. pombe)	-2,53
6209	RPS15	ribosomal protein S15	-2,54
27069	GHITM	growth hormone inducible transmembrane protein	-2,54
2130	EWSR1	Ewing sarcoma breakpoint region 1	-2,54
11180	WDR6	WD repeat domain 6	-2,55
6208	RPS14	ribosomal protein S14	-2,55
644029	ILMN_34790	PREDICTED: Homo sapiens similar to 60S ribosomal protein L7a (LOC644029), mRNA.	-2,55
6209	RPS15	ribosomal protein S15	-2,57
6210	RPS15A	ribosomal protein S15a	-2,59
6155	RPL27	ribosomal protein L27	-2,6
152007	GLIPR2	GLI pathogenesis-related 2	-2,6
10146	G3BP1	GTPase activating protein (SH3 domain) binding protein 1	-2,6
6233	RPS27A	ribosomal protein S27a	-2,63
5997	RGS2	regulator of G-protein signaling 2, 24kDa	-2,63
4332	MNDA	myeloid cell nuclear differentiation antigen	-2,64
654194	ILMN_31988	PREDICTED: Homo sapiens similar to ribosomal protein S27 (LOC654194), mRNA.	-2,66
6203	RPS9	ribosomal protein S9	-2,67
864	RUNX3	runt-related transcription factor 3	-2,68
5162	PDHB	pyruvate dehydrogenase (lipoamide) beta	-2,68
3329	HSPD1	heat shock 60kDa protein 1 (chaperonin)	-2,68

4714	NDUFB8	NADH dehydrogenase (ubiquinone) 1 beta subcomplex, 8, 19kDa	-2,69
3921	ILMN_27800	Homo sapiens ribosomal protein SA (RPSA), transcript variant 1, mRNA.	-2,7
6203	RPS9	ribosomal protein S9	-2,73
3326	HSP90AB1	heat shock protein 90kDa alpha (cytosolic), class B member 1	-2,73
653314	ILMN_31607	Homo sapiens similar to ribosomal protein L19 (LOC653314), mRNA.	-2,75
7529	YWHAB	tyrosine 3-monooxygenase/tryptophan 5-monooxygenase activation protein, beta polypeptide	-2,76
2113	ETS1	v-ets erythroblastosis virus E26 oncogene homolog 1 (avian)	-2,76
4670	HNRNPM	heterogeneous nuclear ribonucleoprotein M	-2,77
10412	NSA2	NSA2 ribosome biogenesis homolog (S. cerevisiae)	-2,79
6217	RPS16	ribosomal protein S16	-2,79
2197	FAU	Finkel-Biskis-Reilly murine sarcoma virus (FBR-MuSV) ubiquitously expressed	-2,79
6170	RPL39	ribosomal protein L39	-2,8
6472	SHMT2	serine hydroxymethyltransferase 2 (mitochondrial)	-2,82
651436	ILMN_39119	PREDICTED: Homo sapiens similar to ribosomal protein L9 (LOC651436), mRNA.	-2,84
6232	RPS27	ribosomal protein S27	-2,86
23524	SRRM2	serine/arginine repetitive matrix 2	-2,87
125144	ILMN_6167	Homo sapiens chromosome 17 open reading frame 45 (C17orf45), mRNA.	-2,89
6722	ILMN_22299	Homo sapiens serum response factor (c-fos serum response element-binding transcription factor) (SRF), mRNA.	-2,9
8241	RBM10	RNA binding motif protein 10	-2,91
4050	LTB	lymphotoxin beta (TNF superfamily, member 3)	-2,91
3122	HLA-DRA	major histocompatibility complex, class II, DR alpha	-2,94
1937	EEF1G	eukaryotic translation elongation factor 1 gamma	-2,97
972	CD74	CD74 molecule, major histocompatibility complex, class II invariant chain	-2,99
55571	C2orf29	chromosome 2 open reading frame 29	-3,02
79026	AHNAK	AHNAK nucleoprotein	-3,02
6218	RPS17	ribosomal protein S17	-3,03
3550	IK	IK cytokine, down-regulator of HLA II	-3,03
59339	PLEKHA2	pleckstrin homology domain containing, family A (phosphoinositide binding specific) member 2	-3,04
3184	HNRNPD	heterogeneous nuclear ribonucleoprotein D (AU-rich element RNA binding protein 1, 37kDa)	-3,05
6156	RPL30	ribosomal protein L30	-3,09
353514	LILRA5	leukocyte immunoglobulin-like receptor, subfamily A (with TM domain), member 5	-3,12
29997	GLTSCR2	glioma tumor suppressor candidate region gene 2	-3,12
6143	RPL19	ribosomal protein L19	-3,15

10365	KLF2	Kruppel-like factor 2 (lung)	-3,17
1974	EIF4A2	eukaryotic translation initiation factor 4A2	-3,19
972	CD74	CD74 molecule, major histocompatibility complex, class II invariant chain	-3,19
3689	ITGB2	integrin, beta 2 (complement component 3 receptor 3 and 4 subunit)	-3,2
302	ANXA2	annexin A2	-3,2
684	BST2	bone marrow stromal cell antigen 2	-3,22
6160	RPL31	ribosomal protein L31	-3,26
526	ATP6V1B2	ATPase, H+ transporting, lysosomal 56/58kDa, V1 subunit B2	-3,27
3936	LCP1	lymphocyte cytosolic protein 1 (L-plastin)	-3,49
3689	ILMN_138267	Homo sapiens integrin, beta 2 (antigen CD18 (p95), lymphocyte function-associated antigen 1; macrophage antigen 1 (mac-1) beta subunit) (ITGB2), mRNA.	-3,54
678	ZFP36L2	zinc finger protein 36, C3H type-like 2	-3,62
3122	HLA-DRA	major histocompatibility complex, class II, DR alpha	-3,62
4069	LYZ	lysozyme	-3,88

Supplementary Table S2: List of deregulated genes in serum of treated patients in comparision to serum of healthy individuals ($p < 0.05$, logFC ≥ 1).

Supplementary Table S2.
List of deregulated genes in serum of treated patients in comparision to serum of healthy individuals ($p < 0.05$, logFC ≥ 1).

ENTREZ Gene ID	Symbol	Gene Name	logFC
8736	MYOM1	myomesin 1, 185kDa	4
3690	ITGB3	integrin, beta 3 (platelet glycoprotein IIIa, antigen CD61)	3,8
8407	TAGLN2	transgelin 2	3,77
9240	PNMA1	paraneoplastic antigen MA1	3,7
3796	KIF2A	kinesin heavy chain member 2A	3,67
27102	EIF2AK1	eukaryotic translation initiation factor 2-alpha kinase 1	3,6
6648	SOD2	superoxide dismutase 2, mitochondrial	3,55
25907	TMEM158	transmembrane protein 158 (gene/pseudogene)	3,54
4900	NRGN	neurogranin (protein kinase C substrate, RC3)	3,44
58497	PRUNE	prune homolog (Drosophila)	3,38
400073	C12orf76	chromosome 12 open reading frame 76	3,34
51257	C3HC4	membrane-associated ring finger (C3HC4) 2	3,28
87	ACTN1	actinin, alpha 1	3,2
7414	VCL	vinculin	3,19
10867	TSPAN9	tetraspanin 9	3,12
2108	ETFA	electron-transfer-flavoprotein, alpha polypeptide	3,12
2791	GNG11	guanine nucleotide binding protein (G protein), gamma 11	3,06
8848	TSC22D1	TSC22 domain family, member 1	3,05
2040	STOM	stomatin	3,02
10653	SPINT2	serine peptidase inhibitor, Kunitz type, 2	3,01
5110	PCMT1	protein-L-isoaspartate (D-aspartate) O-methyltransferase	3,01
830	CAPZA2	capping protein (actin filament) muscle Z-line, alpha 2	3,01
7035	TFPI	tissue factor pathway inhibitor (lipoprotein-associated coagulation inhibitor)	2,98

8407	TAGLN2	transgelin 2	2,96
83699	ILMN_9801	Homo sapiens SH3 domain binding glutamic acid-rich protein like 2 (SH3BGRL2), mRNA.	2,96
5889	RAD51C	RAD51 homolog C (S. cerevisiae)	2,94
8349	ILMN_28293	Homo sapiens histone cluster 2, H2be (HIST2H2BE), mRNA.	2,91
55755	CDK5RAP2	CDK5 regulatory subunit associated protein 2	2,9
10826	C5orf4	chromosome 5 open reading frame 4	2,88
351	APP	amyloid beta (A4) precursor protein	2,88
7035	TFPI	tissue factor pathway inhibitor (lipoprotein-associated coagulation inhibitor)	2,87
89872	AQP10	aquaporin 10	2,85
10611	PDLIM5	PDZ and LIM domain 5	2,82
3934	LCN2	lipocalin 2	2,82
3987	LIMS1	LIM and senescent cell antigen-like domains 1	2,81
444	ASPH	aspartate beta-hydroxylase	2,8
8545	CGGBP1	CGG triplet repeat binding protein 1	2,79
145781	GCOM1	GRINL1A complex locus	2,75
5154	PDGFA	platelet-derived growth factor alpha polypeptide	2,74
8992	ATP6V0E1	ATPase, H+ transporting, lysosomal 9kDa, V0 subunit e1	2,74
28996	HIPK2	homeodomain interacting protein kinase 2	2,73
9948	WDR1	WD repeat domain 1	2,72
196527	ANO6	anoctamin 6	2,72
4673	NAP1L1	nucleosome assembly protein 1-like 1	2,7
8459	TPST2	tyrosylprotein sulfotransferase 2	2,69
10666	CD226	CD226 molecule	2,69
51266	CLEC1B	C-type lectin domain family 1, member B	2,68
9910	RABGAP1L	RAB GTPase activating protein 1-like	2,65
8440	NCK2	NCK adaptor protein 2	2,65
9848	MFAP3L	microfibrillar-associated protein 3-like	2,65
1200	ILMN_19883	Homo sapiens tripeptidyl peptidase I (TPP1), mRNA.	2,64
5816	PVALB	parvalbumin	2,64
25994	HIGD1A	HIG1 hypoxia inducible domain family, member 1A	2,64
54741	ILMN_27032	Homo sapiens leptin receptor overlapping transcript (LEPROT), mRNA.	2,63
27292	DIMT1	DIM1 dimethyladenosine transferase 1 homolog (S. cerevisiae)	2,63
340205	TREML1	triggering receptor expressed on myeloid cells-like 1	2,61

51109	RDH11	retinol dehydrogenase 11 (all-trans/9-cis/11-cis)	2,61
7456	WIPF1	WAS/WASL interacting protein family, member 1	2,6
5725	PTBP1	polypyrimidine tract binding protein 1	2,6
2983	GUCY1B3	guanylate cyclase 1, soluble, beta 3	2,6
4218	RAB8A	RAB8A, member RAS oncogene family	2,58
5567	PRKACB	protein kinase, cAMP-dependent, catalytic, beta	2,58
649260	ILMN_35781	PREDICTED: Homo sapiens similar to LIM and senescent cell antigen-like domains 1 (LOC649260), mRNA.	2,57
3987	LIMS1	LIM and senescent cell antigen-like domains 1	2,57
3693	ITGB5	integrin, beta 5	2,57
2017	CTTN	cortactin	2,56
1069	CETN2	centrin, EF-hand protein, 2	2,56
8417	STX7	syntaxin 7	2,55
2162	F13A1	coagulation factor XIII, A1 polypeptide	2,55
10079	ATP9A	ATPase, class II, type 9A	2,55
401232	ILMN_11265	Homo sapiens hypothetical protein DKFZp686I15217 (DKFZp686I15217), mRNA.	2,53
4638	MYLK	myosin light chain kinase	2,52
	ILMN_72868	TCAAP1D4752 Pediatric acute myelogenous leukemia cell (FAB M1) , mRNA sequence	2,51
3017	HIST1H2BD	histone cluster 1, H2bd	2,48
7057	THBS1	thrombospondin 1	2,47
2982	GUCY1A3	guanylate cyclase 1, soluble, alpha 3	2,46
222166	C7orf41	chromosome 7 open reading frame 41	2,46
55845	BRK1	BRICK1, SCAR/WAVE actin-nucleating complex subunit	2,44
3688	ITGB1	integrin, beta 1 (fibronectin receptor, beta polypeptide, antigen CD29 includes MDF2, MSK12)	2,43
55353	LAPTM4B	lysosomal protein transmembrane 4 beta	2,42
10217	CTDSPL	CTD (carboxy-terminal domain, RNA polymerase II, polypeptide A) small phosphatase-like	2,42
400073	C12orf76	chromosome 12 open reading frame 76	2,42
401232	ILMN_11265	Homo sapiens hypothetical protein DKFZp686I15217 (DKFZp686I15217), mRNA.	2,41
222658	KCTD20	potassium channel tetramerisation domain containing 20	2,4
3688	ITGB1	integrin, beta 1 (fibronectin receptor, beta polypeptide, antigen CD29 includes MDF2, MSK12)	2,4
402	ARL2	ADP-ribosylation factor-like 2	2,4
5627	PROS1	protein S (alpha)	2,39
81027	TUBB1	tubulin, beta 1	2,38
351	APP	amyloid beta (A4) precursor protein	2,38

644422	ILMN_34341	PREDICTED: Homo sapiens misc_RNA (LOC644422), miscRNA.	2,37
1998	ELF2	E74-like factor 2 (ets domain transcription factor)	2,37
5476	CTSA	cathepsin A	2,37
286006	C7orf53	chromosome 7 open reading frame 53	2,37
7414	VCL	vinculin	2,36
2664	ILMN_13492	Homo sapiens GDP dissociation inhibitor 1 (GDI1), mRNA.	2,35
23429	RYBP	RING1 and YY1 binding protein	2,34
8655	DYNLL1	dynein, light chain, LC8-type 1	2,34
51706	CYB5R1	cytochrome b5 reductase 1	2,33
7531	YWHAE	tyrosine 3-monooxygenase/tryptophan 5-monooxygenase activation protein, epsilon polypeptide	2,32
64407	RGS18	regulator of G-protein signaling 18	2,32
64795	RMND5A	required for meiotic nuclear division 5 homolog A (S. cerevisiae)	2,31
10611	PDLIM5	PDZ and LIM domain 5	2,31
3580	ILMN_139000	PREDICTED: Homo sapiens interleukin 8 receptor, beta pseudogene (IL8RBP), misc RNA.	2,31
55294	FBXW7	F-box and WD repeat domain containing 7	2,31
5742	ILMN_3001	Homo sapiens prostaglandin-endoperoxide synthase 1 (PTGS1), transcript variant 2, mRNA.	2,3
3987	LIMS1	LIM and senescent cell antigen-like domains 1	2,29
8655	DYNLL1	dynein, light chain, LC8-type 1	2,29
6374	CXCL5	chemokine (C-X-C motif) ligand 5	2,29
83706	FERMT3	fermitin family member 3	2,28
5413	ILMN_139223	Homo sapiens septin 5 (SEPT5), mRNA.	2,27
23531	MMD	monocyte to macrophage differentiation-associated	2,27
63933	CCDC90A	coiled-coil domain containing 90A	2,27
55132	LARP1B	La ribonucleoprotein domain family, member 1B	2,26
2149	ILMN_25961	Homo sapiens coagulation factor II (thrombin) receptor (F2R), mRNA.	2,25
23558	WBP2	WW domain binding protein 2	2,24
8345	HIST1H2BH	histone cluster 1, H2bh	2,23
948	CD36	CD36 molecule (thrombospondin receptor)	2,23
7533	YWHAH	tyrosine 3-monooxygenase/tryptophan 5-monooxygenase activation protein, eta polypeptide	2,22
5795	PTPRJ	protein tyrosine phosphatase, receptor type, J	2,22
2876	ILMN_10376	Homo sapiens glutathione peroxidase 1 (GPX1), transcript variant 2, mRNA.	2,22
10398	MYL9	myosin, light chain 9, regulatory	2,21
25994	HIGD1A	HIG1 hypoxia inducible domain family, member 1A	2,2

643319	ILMN_41137	PREDICTED: Homo sapiens similar to Transgelin-2 (LOC643319), mRNA.	2,18
112495	GTF3C6	general transcription factor IIIC, polypeptide 6, alpha 35kDa	2,18
5494	PPM1A	protein phosphatase, Mg2+/Mn2+ dependent, 1A	2,17
26052	DNM3	dynamin 3	2,17
4953	ODC1	ornithine decarboxylase 1	2,16
54516	ILMN_182901	Homo sapiens mitochondrial translational release factor 1-like (MTRF1L), mRNA.	2,16
4211	MEIS1	Meis homeobox 1	2,16
5867	ILMN_14453	Homo sapiens RAB4A, member RAS oncogene family (RAB4A), mRNA.	2,15
27018	NGFRAP1	nerve growth factor receptor (TNFRSF16) associated protein 1	2,15
4267	CD99	CD99 molecule	2,15
286451	YIPF6	Yip1 domain family, member 6	2,14
8848	TSC22D1	TSC22 domain family, member 1	2,14
442124	ILMN_37787	PREDICTED: Homo sapiens hypothetical LOC442124 (LOC442124), mRNA.	2,14
387820	ILMN_26714	PREDICTED: Homo sapiens similar to DnaJ (Hsp40) homolog, subfamily B, member 6 isoform a (LOC387820), mRNA.	2,13
643253	CCT6P1	chaperonin containing TCP1, subunit 6 (zeta) pseudogene 1	2,13
3146	HMGB1	high mobility group box 1	2,12
6648	SOD2	superoxide dismutase 2, mitochondrial	2,11
139105	ILMN_3923	Homo sapiens chromosome X open reading frame 20 (CXorf20), mRNA.	2,11
9848	MFAP3L	microfibrillar-associated protein 3-like	2,1
401629	ILMN_14074	Homo sapiens hypothetical LOC401629 (LOC401629), non-coding RNA.	2,1
54469	ZFAND6	zinc finger, AN1-type domain 6	2,09
7504	XK	X-linked Kx blood group (McLeod syndrome)	2,09
51251	NT5C3	5'-nucleotidase, cytosolic III	2,09
8766	RAB11A	RAB11A, member RAS oncogene family	2,08
441081	ILMN_36410	PREDICTED: Homo sapiens similar to nuclear pore membrane protein 121 (LOC441081), mRNA.	2,08
5552	SRGN	serglycin	2,07
4833	NME4	non-metastatic cells 4, protein expressed in	2,07
6374	CXCL5	chemokine (C-X-C motif) ligand 5	2,07
6622	SNCA	synuclein, alpha (non A4 component of amyloid precursor)	2,06
83700	JAM3	junctional adhesion molecule 3	2,06
3611	ILK	integrin-linked kinase	2,06
2280	FKBP1A	FK506 binding protein 1A, 12kDa	2,06
2040	STOM	stomatin	2,05

4627	MYH9	myosin, heavy chain 9, non-muscle	2,05
651361	ILMN_35263	PREDICTED: Homo sapiens similar to S-phase kinase-associated protein 1A (Cyclin A/CDK2-associated protein p19) (p19A) (p19skp1) (LOC651361), mRNA.	2,04
9445	ITM2B	integral membrane protein 2B	2,04
83658	DYNLRB1	dynein, light chain, roadblock-type 1	2,04
4052	LTBP1	latent transforming growth factor beta binding protein 1	2,03
1026	ILMN_5895	Homo sapiens cyclin-dependent kinase inhibitor 1A (p21, Cip1) (CDKN1A), transcript variant 1, mRNA.	2,03
6638	ILMN_17485	Homo sapiens small nuclear ribonucleoprotein polypeptide N (SNRPN), transcript variant 3, mRNA.	2,02
83658	DYNLRB1	dynein, light chain, roadblock-type 1	2,02
10929	SRSF8	serine/arginine-rich splicing factor 8	2
27020	NPTN	neuroplastin	2
4149	MAX	MYC associated factor X	2
	ILMN_110914	BX429982 Homo sapiens FETAL LIVER Homo sapiens cDNA clone CS0DM002YI05 5-PRIME, mRNA sequence	1,99
257019	FRMD3	FERM domain containing 3	1,99
23299	BICD2	bicaudal D homolog 2 (Drosophila)	1,99
84519	ACRBP	acrosin binding protein	1,99
84859	LRCH3	leucine-rich repeats and calponin homology (CH) domain containing 3	1,97
641649	TMEM91	transmembrane protein 91	1,96
8428	STK24	serine/threonine kinase 24	1,96
6648	ILMN_19880	Homo sapiens superoxide dismutase 2, mitochondrial (SOD2), nuclear gene encoding mitochondrial protein, transcript variant 1, mRNA.	1,96
3048	HBG2	hemoglobin, gamma G	1,95
5782	PTPN12	protein tyrosine phosphatase, non-receptor type 12	1,94
5606	MAP2K3	mitogen-activated protein kinase kinase 3	1,94
2534	FYN	FYN oncogene related to SRC, FGR, YES	1,94
6638	ILMN_11685	Homo sapiens small nuclear ribonucleoprotein polypeptide N (SNRPN), transcript variant 3, mRNA.	1,93
5087	PBX1	pre-B-cell leukemia homeobox 1	1,93
9054	NFS1	NFS1 nitrogen fixation 1 homolog (S. cerevisiae)	1,93
55796	MBNL3	muscleblind-like 3 (Drosophila)	1,93
645128	ILMN_40515	PREDICTED: Homo sapiens similar to DEAD/H (Asp-Glu-Ala-Asp/His) box polypeptide 11 isoform 1 (LOC645128), mRNA.	1,93
1175	AP2S1	adaptor-related protein complex 2, sigma 1 subunit	1,93
6678	ILMN_1780	Homo sapiens secreted protein, acidic, cysteine-rich (osteonectin) (SPARC), mRNA.	1,92

5725	PTBP1	polypyrimidine tract binding protein 1	1,92
5577	PRKAR2B	protein kinase, cAMP-dependent, regulatory, type II, beta	1,92
79752	ILMN_23674	Homo sapiens zinc finger, AN1-type domain 1 (ZFAND1), mRNA.	1,91
5874	RAB27B	RAB27B, member RAS oncogene family	1,91
3081	HGD	homogentisate 1,2-dioxygenase	1,91
80212	CCDC92	coiled-coil domain containing 92	1,91
54498	SMOX	spermine oxidase	1,9
4735	2.IX	septin 2	1,9
9124	PDLIM1	PDZ and LIM domain 1	1,9
9445	ITM2B	integral membrane protein 2B	1,9
	ILMN_89656	yz74c09.s1 Soares_multiple_sclerosis_2NbHMSP Homo sapiens cDNA clone IMAGE:288784 3, mRNA sequence	1,9
10357	HMGB1P1	high mobility group box 1 pseudogene 1	1,9
83541	FAM110A	family with sequence similarity 110, member A	1,9
9191	DEDD	death effector domain containing	1,9
649214	ILMN_33279	PREDICTED: Homo sapiens similar to High mobility group protein 1-like 10 (HMG-1L10), transcript variant 4 (LOC649214), mRNA.	1,89
128989	C22orf25	chromosome 22 open reading frame 25	1,89
153768	PRELID2	PRELI domain containing 2	1,88
11315	PARK7	parkinson protein 7	1,88
153527	ZMAT2	zinc finger, matrin-type 2	1,87
5197	PF4V1	platelet factor 4 variant 1	1,87
5196	ILMN_16715	Homo sapiens platelet factor 4 (chemokine (C-X-C motif) ligand 4) (PF4), mRNA.	1,87
10092	ARPC5	actin related protein 2/3 complex, subunit 5, 16kDa	1,87
22821	RASA3	RAS p21 protein activator 3	1,86
51251	NT5C3	5'-nucleotidase, cytosolic III	1,86
56890	MDM1	Mdm1 nuclear protein homolog (mouse)	1,86
92092	ZC3HAV1L	zinc finger CCCH-type, antiviral 1-like	1,85
22920	KIFAP3	kinesin-associated protein 3	1,85
1729	DIAPH1	diaphanous homolog 1 (Drosophila)	1,85
22885	ABLIM3	actin binding LIM protein family, member 3	1,85
8926	ILMN_15998	Homo sapiens SNRPN upstream reading frame (SNURF), transcript variant 1, mRNA.	1,83
4638	MYLK	myosin light chain kinase	1,83
644799	ILMN_43702	PREDICTED: Homo sapiens hypothetical protein LOC644799, transcript variant 1 (LOC644799), mRNA.	1,83

643138	ILMN_39430	PREDICTED: Homo sapiens similar to Guanine nucleotide-binding protein G(s), alpha subunit (Adenylate cyclase-stimulating G alpha protein) (G-alpha-8) (LOC643138), mRNA.	1,83
3988	LIPA	lipase A, lysosomal acid, cholesterol esterase	1,83
8328	GFI1B	growth factor independent 1B transcription repressor	1,83
6352	CCL5	chemokine (C-C motif) ligand 5	1,83
56897	WRNIP1	Werner helicase interacting protein 1	1,82
10477	UBE2E3	ubiquitin-conjugating enzyme E2E 3	1,82
94121	SYTL4	synaptotagmin-like 4	1,82
5305	PIP4K2A	phosphatidylinositol-5-phosphate 4-kinase, type II, alpha	1,82
3987	ILMN_139143	PREDICTED: Homo sapiens LIM and senescent cell antigen-like domains 1 (LIMS1), mRNA.	1,82
2811	GP1BA	glycoprotein Ib (platelet), alpha polypeptide	1,82
91624	NEXN	nexilin (F actin binding protein)	1,81
6500	SKP1	S-phase kinase-associated protein 1	1,8
10981	RAB32	RAB32, member RAS oncogene family	1,8
114769	CARD16	caspase recruitment domain family, member 16	1,79
55122	AKIRIN2	akirin 2	1,79
339005	WHAMMP3	WAS protein homolog associated with actin, golgi membranes and microtubules pseudogene 3	1,78
	ILMN_93150	UI-H-EU0-azk-k-10-0-UI.s1 NCI_CGAP_Car1 Homo sapiens cDNA clone IMAGE: 5849985 3, mRNA sequence	1,78
80790	ILMN_13851	Homo sapiens c-Maf-inducing protein (CMIP), transcript variant Tc-mip, mRNA.	1,78
9839	ZEB2	zinc finger E-box binding homeobox 2	1,77
27257	LSM1	LSM1 homolog, U6 small nuclear RNA associated (S. cerevisiae)	1,77
3688	ITGB1	integrin, beta 1 (fibronectin receptor, beta polypeptide, antigen CD29 includes MDF2, MSK12)	1,77
56344	CABP5	calcium binding protein 5	1,77
351	APP	amyloid beta (A4) precursor protein	1,77
4154	MBNL1	muscleblind-like (Drosophila)	1,76
	ILMN_88177	Homo sapiens cDNA clone IMAGE:5261213	1,76
488	ILMN_19965	Homo sapiens ATPase, Ca++ transporting, cardiac muscle, slow twitch 2 (ATP2A2), transcript variant 1, mRNA.	1,76
7485	WRB	tryptophan rich basic protein	1,75
9978	RBX1	ring-box 1, E3 ubiquitin protein ligase	1,75
94274	PPP1R14A	protein phosphatase 1, regulatory (inhibitor) subunit 14A	1,75
	ILMN_115478	UI-E-EO0-ahy-o-21-0-UI.s1 UI-E-EO0 Homo sapiens cDNA clone UI-E-EO0-ahy-o-21-0-UI 3, mRNA sequence	1,75
9588	PRDX6	peroxiredoxin 6	1,74
10398	MYL9	myosin, light chain 9, regulatory	1,74

6009	RHEB	Ras homolog enriched in brain	1,73
6774	STAT3	signal transducer and activator of transcription 3 (acute-phase response factor)	1,72
6117	RPA1	replication protein A1, 70kDa	1,72
651723	ILMN_39567	PREDICTED: Homo sapiens similar to schlafen family member 13 (LOC651723), mRNA.	1,72
387882	ILMN_23241	Homo sapiens hypothetical protein (LOC387882), mRNA.	1,72
57110	HRASLS	HRAS-like suppressor	1,72
25932	CLIC4	chloride intracellular channel 4	1,72
808	CALM3	calmodulin 3 (phosphorylase kinase, delta)	1,72
80739	C6orf25	chromosome 6 open reading frame 25	1,72
30061	SLC40A1	solute carrier family 40 (iron-regulated transporter), member 1	1,71
5911	RAP2A	RAP2A, member of RAS oncogene family	1,71
2280	FKBP1A	FK506 binding protein 1A, 12kDa	1,71
682	BSG	basigin (Ok blood group)	1,71
4627	MYH9	myosin, heavy chain 9, non-muscle	1,69
643752	RAP1BL	RAP1B, member of RAS oncogene family pseudogene	1,69
54498	SMOX	spermine oxidase	1,68
6117	RPA1	replication protein A1, 70kDa	1,68
5782	PTPN12	protein tyrosine phosphatase, non-receptor type 12	1,68
5341	PLEK	pleckstrin	1,68
27040	LAT	linker for activation of T cells	1,68
	ILMN_125202	IL2-NT0102-160600-105-C03 NT0102 Homo sapiens cDNA, mRNA sequence	1,68
	ILMN_123473	wf43g03.x1 Soares_NFL_T_GBC_S1 Homo sapiens cDNA clone IMAGE:2358388 3, mRNA sequence	1,68
	ILMN_121109	Homo sapiens mRNA; cDNA DKFZp762M127 (from clone DKFZp762M127)	1,68
267	AMFR	autocrine motility factor receptor	1,68
51646	YPEL5	yippee-like 5 (Drosophila)	1,67
64805	P2RY12	purinergic receptor P2Y, G-protein coupled, 12	1,67
136319	MTPN	myotrophin	1,67
10049	DNAJB6	DnaJ (Hsp40) homolog, subfamily B, member 6	1,67
51185	CRBN	cereblon	1,67
598	BCL2L1	BCL2-like 1	1,67
8654	PDE5A	phosphodiesterase 5A, cGMP-specific	1,66
23603	CORO1C	coronin, actin binding protein, 1C	1,66
375033	ILMN_170351	Homo sapiens platelet endothelial aggregation receptor 1 (PEAR1), mRNA.	1,65

391475	DYTN	dystrotelin	1,65
28996	HIPK2	homeodomain interacting protein kinase 2	1,65
2500	ILMN_16099	Homo sapiens ferritin, heavy polypeptide-like 7 (FTHL7) on chromosome 13.	1,65
10000	AKT3	v-akt murine thymoma viral oncogene homolog 3 (protein kinase B, gamma)	1,65
5606	MAP2K3	mitogen-activated protein kinase kinase 3	1,64
644162	ILMN_43225	PREDICTED: Homo sapiens similar to septin 7, transcript variant 4 (LOC644162), mRNA.	1,64
1523	CUX1	cut-like homeobox 1	1,64
6668	SP2	Sp2 transcription factor	1,63
4735	2.IX	septin 2	1,63
9554	SEC22B	SEC22 vesicle trafficking protein homolog B (S. cerevisiae) (gene/pseudogene)	1,63
8436	SDPR	serum deprivation response	1,63
11235	PDCD10	programmed cell death 10	1,63
4154	MBNL1	muscleblind-like (Drosophila)	1,63
440917	LOC440917	tyrosine 3-monooxygenase/tryptophan 5-monooxygenase activation protein, epsilon polypeptide pseudogene	1,63
6399	TRAPPC2	trafficking protein particle complex 2	1,62
6478	SIAH2	seven in absentia homolog 2 (Drosophila)	1,62
27040	ILMN_30259	Homo sapiens linker for activation of T cells (LAT), transcript variant 2, mRNA.	1,62
9446	GSTO1	glutathione S-transferase omega 1	1,62
2717	GLA	galactosidase, alpha	1,62
55281	TMEM140	transmembrane protein 140	1,61
64114	TMBIM1	transmembrane BAX inhibitor motif containing 1	1,61
26994	RNF11	ring finger protein 11	1,61
2026	ENO2	enolase 2 (gamma, neuronal)	1,61
56911	C21orf7	chromosome 21 open reading frame 7	1,61
10357	HMGB1P1	high mobility group box 1 pseudogene 1	1,6
25852	ARMC8	armadillo repeat containing 8	1,6
51720	UIMC1	ubiquitin interaction motif containing 1	1,59
644380	ILMN_36938	PREDICTED: Homo sapiens similar to High mobility group protein 1-like 10 (HMG-1L10), transcript variant 1 (LOC644380), mRNA.	1,59
387820	ILMN_26714	PREDICTED: Homo sapiens similar to DnaJ (Hsp40) homolog, subfamily B, member 6 isoform a (LOC387820), mRNA.	1,59
	ILMN_100075	Homo sapiens cDNA FLJ37325 fis, clone BRAMY2018295	1,59
7871	SLMAP	sarcolemma associated protein	1,58
147199	SCGB1C1	secretoglobin, family 1C, member 1	1,57

11031	RAB31	RAB31, member RAS oncogene family	1,57
10857	PGRMC1	progesterone receptor membrane component 1	1,57
58485	TRAPPC1	trafficking protein particle complex 1	1,56
9861	PSMD6	proteasome (prosome, macropain) 26S subunit, non-ATPase, 6	1,56
8337	ILMN_26733	Homo sapiens histone cluster 2, H2aa3 (HIST2H2AA3), mRNA.	1,56
7076	TIMP1	TIMP metallopeptidase inhibitor 1	1,55
27018	NGFRAP1	nerve growth factor receptor (TNFRSF16) associated protein 1	1,55
2309	FOXO3	forkhead box O3	1,55
51566	ARMCX3	armadillo repeat containing, X-linked 3	1,55
25893	TRIM58	tripartite motif containing 58	1,54
4860	PNP	purine nucleoside phosphorylase	1,54
27030	MLH3	mutL homolog 3 (E. coli)	1,54
3315	HSPB1	heat shock 27kDa protein 1	1,54
1727	CYB5R3	cytochrome b5 reductase 3	1,54
395	ARHGAP6	Rho GTPase activating protein 6	1,54
226	ALDOA	aldolase A, fructose-bisphosphate	1,54
10169	SERF2	small EDRK-rich factor 2	1,53
219771	CCNY	cyclin Y	1,53
83707	ILMN_9909	Homo sapiens tRNA phosphotransferase 1 (TRPT1), transcript variant 2, mRNA.	1,52
8773	SNAP23	synaptosomal-associated protein, 23kDa	1,52
4735	2.IX	septin 2	1,52
6403	SELP	selectin P (granule membrane protein 140kDa, antigen CD62)	1,52
441155	ILMN_35976	PREDICTED: Homo sapiens similar to Zinc finger CCCH-type domain-containing protein 11A, transcript variant 5 (LOC441155), mRNA.	1,52
51094	ADIPOR1	adiponectin receptor 1	1,52
6789	STK4	serine/threonine kinase 4	1,51
9628	RGS6	regulator of G-protein signaling 6	1,51
123803	NTAN1	N-terminal asparagine amidase	1,51
112597	ILMN_137135	Homo sapiens hypothetical protein MGC4677 (MGC4677), mRNA.	1,51
127018	LYPLAL1	lysophospholipase-like 1	1,51
29094	LGALSL	lectin, galactoside-binding-like	1,51
56940	ILMN_138115	PREDICTED: Homo sapiens dual specificity phosphatase 22 (DUSP22), mRNA.	1,51
6001	RGS10	regulator of G-protein signaling 10	1,5

5315	PKM2	pyruvate kinase, muscle	1,5
	ILMN_81934	UI-H-DF1-auk-p-10-0-UI.s1 NCI_CGAP_DF1 Homo sapiens cDNA clone IMAGE:5870841 3, mRNA sequence	1,5
326624	RAB37	RAB37, member RAS oncogene family	1,49
8031	NCOA4	nuclear receptor coactivator 4	1,49
55256	ADI1	acireductone dioxygenase 1	1,49
23032	USP33	ubiquitin specific peptidase 33	1,48
5870	RAB6A	RAB6A, member RAS oncogene family	1,48
	ILMN_103084	BX105743 NCI_CGAP_GC6 Homo sapiens cDNA clone IMAGp998H135716, mRNA sequence	1,48
	ILMN_97811	AV762101 MDS Homo sapiens cDNA clone MDSEOA03 5, mRNA sequence	1,48
8348	HIST1H2BO	histone cluster 1, H2bo	1,48
25852	ARMC8	armadillo repeat containing 8	1,48
57584	ARHGAP21	Rho GTPase activating protein 21	1,48
51538	ZCCHC17	zinc finger, CCHC domain containing 17	1,47
340348	TSPAN33	tetraspanin 33	1,47
4149	MAX	MYC associated factor X	1,47
26119	LDLRAP1	low density lipoprotein receptor adaptor protein 1	1,47
9923	ZBTB40	zinc finger and BTB domain containing 40	1,46
53346	TM6SF1	transmembrane 6 superfamily member 1	1,46
8773	SNAP23	synaptosomal-associated protein, 23kDa	1,46
4588	MUC6	mucin 6, oligomeric mucus/gel-forming	1,46
	ILMN_106785	fg13g01.x1 Human Iris cDNA (Normalized): fg Homo sapiens cDNA clone fg13g01 3, mRNA sequence	1,46
51727	CMPK1	cytidine monophosphate (UMP-CMP) kinase 1, cytosolic	1,46
5552	SRGN	serglycin	1,45
5062	PAK2	p21 protein (Cdc42/Rac)-activated kinase 2	1,45
653635	ILMN_42161	PREDICTED: Homo sapiens similar to CXYorf1-related protein (LOC653635), mRNA.	1,45
89857	KLHL6	kelch-like 6 (Drosophila)	1,45
3017	HIST1H2BD	histone cluster 1, H2bd	1,45
2778	GNAS	GNAS complex locus	1,45
57818	G6PC2	glucose-6-phosphatase, catalytic, 2	1,45
8654	PDE5A	phosphodiesterase 5A, cGMP-specific	1,44
3047	HBG1	hemoglobin, gamma A	1,44
10152	ABI2	abl-interactor 2	1,44
137492	VPS37A	vacuolar protein sorting 37 homolog A (S. cerevisiae)	1,43

30061	SLC40A1	solute carrier family 40 (iron-regulated transporter), member 1	1,43
4354	MPP1	membrane protein, palmitoylated 1, 55kDa	1,43
650263	ILMN_46692	PREDICTED: Homo sapiens hypothetical protein LOC650263 (LOC650263), mRNA.	1,43
11345	GABARAPL2	GABA(A) receptor-associated protein-like 2	1,43
2207	FCER1G	Fc fragment of IgE, high affinity I, receptor for; gamma polypeptide	1,43
760	CA2	carbonic anhydrase II	1,43
7353	UFD1L	ubiquitin fusion degradation 1 like (yeast)	1,42
4917	NTN3	netrin 3	1,42
10093	ARPC4	actin related protein 2/3 complex, subunit 4, 20kDa	1,42
10000	AKT3	v-akt murine thymoma viral oncogene homolog 3 (protein kinase B, gamma)	1,42
7292	TNFSF4	tumor necrosis factor (ligand) superfamily, member 4	1,41
9764	KIAA0513	KIAA0513	1,41
2820	GPD2	glycerol-3-phosphate dehydrogenase 2 (mitochondrial)	1,41
2010	EMD	emerin	1,41
10209	EIF1	eukaryotic translation initiation factor 1	1,41
22830	ILMN_32336	PREDICTED: Homo sapiens zinc finger protein 409 (ZNF409), mRNA.	1,4
9087	TMSB4Y	thymosin beta 4, Y-linked	1,4
6613	SUMO2	SMT3 suppressor of mif two 3 homolog 2 (S. cerevisiae)	1,4
29937	NENF	neudesin neurotrophic factor	1,4
8477	GPR65	G protein-coupled receptor 65	1,4
79053	ALG8	asparagine-linked glycosylation 8, alpha-1,3-glucosyltransferase homolog (S. cerevisiae)	1,4
85439	STON2	stonin 2	1,39
23011	RAB21	RAB21, member RAS oncogene family	1,39
2766	GMPR	guanosine monophosphate reductase	1,39
2495	FTH1	ferritin, heavy polypeptide 1	1,39
647499	ILMN_34248	PREDICTED: Homo sapiens similar to CG7889-PA, transcript variant 2 (LOC647499), mRNA.	1,38
81689	ISCA1	iron-sulfur cluster assembly 1 homolog (S. cerevisiae)	1,38
2776	GNAQ	guanine nucleotide binding protein (G protein), q polypeptide	1,38
54780	NSMCE4A	non-SMC element 4 homolog A (S. cerevisiae)	1,37
26065	LSM14A	LSM14A, SCD6 homolog A (S. cerevisiae)	1,37
	ILMN_73823	Homo sapiens cDNA: FLJ23006 fis, clone LNG00414	1,37
84548	TMEM185A	transmembrane protein 185A	1,36
51643	TMBIM4	transmembrane BAX inhibitor motif containing 4	1,36

57142	RTN4	reticulon 4	1,36
25915	NDUFAF3	NADH dehydrogenase (ubiquinone) 1 alpha subcomplex, assembly factor 3	1,36
648057	ILMN_32589	PREDICTED: Homo sapiens similar to Guanine nucleotide-binding protein G(s), alpha subunit (Adenylate cyclase-stimulating G alpha protein) (G-alpha-8) (LOC648057), mRNA.	1,36
	ILMN_71879	Homo sapiens cDNA clone IMAGE:5297467	1,36
10049	DNAJB6	DnaJ (Hsp40) homolog, subfamily B, member 6	1,36
9556	C14orf2	chromosome 14 open reading frame 2	1,36
7511	XPNPEP1	X-prolyl aminopeptidase (aminopeptidase P) 1, soluble	1,35
7351	UCP2	uncoupling protein 2 (mitochondrial, proton carrier)	1,35
6397	SEC14L1	SEC14-like 1 (S. cerevisiae)	1,35
9861	PSMD6	proteasome (prosome, macropain) 26S subunit, non-ATPase, 6	1,35
55329	MNS1	meiosis-specific nuclear structural 1	1,35
23215	PRRC2C	proline-rich coiled-coil 2C	1,35
6431	SRSF6	serine/arginine-rich splicing factor 6	1,34
54470	ARMCX6	armadillo repeat containing, X-linked 6	1,34
55062	WIPI1	WD repeat domain, phosphoinositide interacting 1	1,33
124446	TMEM219	transmembrane protein 219	1,33
5573	PRKAR1A	protein kinase, cAMP-dependent, regulatory, type I, alpha (tissue specific extinguisher 1)	1,33
3572	IL6ST	interleukin 6 signal transducer (gp130, oncostatin M receptor)	1,33
10949	HNRNPA0	heterogeneous nuclear ribonucleoprotein A0	1,33
10681	GNB5	guanine nucleotide binding protein (G protein), beta 5	1,33
1399	CRKL	v-crk sarcoma virus CT10 oncogene homolog (avian)-like	1,33
7009	TMBIM6	transmembrane BAX inhibitor motif containing 6	1,32
6451	SH3BGRL	SH3 domain binding glutamic acid-rich protein like	1,32
3013	HIST1H2AD	histone cluster 1, H2ad	1,32
84310	C7orf50	chromosome 7 open reading frame 50	1,32
55004	LAMTOR1	late endosomal/lysosomal adaptor, MAPK and MTOR activator 1	1,32
51742	ARID4B	AT rich interactive domain 4B (RBP1-like)	1,32
80232	WDR26	WD repeat domain 26	1,31
4130	MAP1A	microtubule-associated protein 1A	1,31
389787	LOC389787	tumor protein, translationally-controlled 1 pseudogene	1,31
51206	GP6	glycoprotein VI (platelet)	1,31
5908	RAP1B	RAP1B, member of RAS oncogene family	1,3

4673	NAP1L1	nucleosome assembly protein 1-like 1	1,3
80177	MYCT1	myc target 1	1,3
3241	HPCAL1	hippocalcin-like 1	1,3
9086	EIF1AY	eukaryotic translation initiation factor 1A, Y-linked	1,3
7750	ZMYM2	zinc finger, MYM-type 2	1,29
80230	RUFY1	RUN and FYVE domain containing 1	1,29
84281	C2orf88	chromosome 2 open reading frame 88	1,29
2815	GP9	glycoprotein IX (platelet)	1,29
10938	EHD1	EH-domain containing 1	1,29
339005	ILMN_32270	Homo sapiens WAS protein homolog associated with actin, golgi membranes and microtubules-like 1 (WHAMML1), non-coding RNA.	1,28
283131	ILMN_138191	PREDICTED: Homo sapiens trophoblast-derived noncoding RNA (TncRNA), misc RNA.	1,28
647025	ILMN_32345	PREDICTED: Homo sapiens hypothetical LOC647025 (LOC647025), mRNA.	1,28
53827	FXYD5	FXYD domain containing ion transport regulator 5	1,28
760	CA2	carbonic anhydrase II	1,28
148823	ILMN_22220	Homo sapiens chromosome 1 open reading frame 150 (C1orf150), mRNA.	1,28
8905	AP1S2	adaptor-related protein complex 1, sigma 2 subunit	1,28
7326	UBE2G1	ubiquitin-conjugating enzyme E2G 1	1,27
90853	SPOCD1	SPOC domain containing 1	1,27
2273	FHL1	four and a half LIM domains 1	1,27
25793	FBXO7	F-box protein 7	1,27
8804	CREG1	cellular repressor of E1A-stimulated genes 1	1,27
377	ARF3	ADP-ribosylation factor 3	1,27
6748	SSR4	signal sequence receptor, delta	1,26
387590	ILMN_12454	Homo sapiens TPTE pseudogene (psiTPTE22), non-coding RNA.	1,26
5352	PLOD2	procollagen-lysine, 2-oxoglutarate 5-dioxygenase 2	1,26
112597	ILMN_18892	Homo sapiens hypothetical protein MGC4677 (MGC4677), mRNA.	1,26
653226	ILMN_39856	PREDICTED: Homo sapiens similar to Signal recognition particle 9 kDa protein (SRP9) (LOC653226), mRNA.	1,26
651919	ILMN_36846	PREDICTED: Homo sapiens similar to Ras-related C3 botulinum toxin substrate 1 (p21-Rac1) (LOC651919), mRNA.	1,26
122773	KLHDC1	kelch domain containing 1	1,26
25994	HIGD1A	HIG1 hypoxia inducible domain family, member 1A	1,26
1982	EIF4G2	eukaryotic translation initiation factor 4 gamma, 2	1,26
1653	DDX1	DEAD (Asp-Glu-Ala-Asp) box polypeptide 1	1,26

8676	STX11	syntaxin 11	1,25
113230	ILMN_33629	PREDICTED: Homo sapiens hypothetical protein LOC113230 (LOC113230), mRNA.	1,25
1603	DAD1	defender against cell death 1	1,25
113457	TUBA3D	tubulin, alpha 3d	1,24
338596	ST8SIA6	ST8 alpha-N-acetyl-neuraminide alpha-2,8-sialyltransferase 6	1,24
1032	CDKN2D	cyclin-dependent kinase inhibitor 2D (p19, inhibits CDK4)	1,24
388403	YPEL2	yippee-like 2 (Drosophila)	1,23
85012	TCEAL3	transcription elongation factor A (SII)-like 3	1,23
136319	MTPN	myotrophin	1,23
81631	MAP1LC3B	microtubule-associated protein 1 light chain 3 beta	1,23
91283	C9orf30	chromosome 9 open reading frame 30	1,23
3105	HLA-A	major histocompatibility complex, class I, A	1,22
1072	CFL1	cofilin 1 (non-muscle)	1,22
85012	TCEAL3	transcription elongation factor A (SII)-like 3	1,21
6622	SNCA	synuclein, alpha (non A4 component of amyloid precursor)	1,21
83442	SH3BGRL3	SH3 domain binding glutamic acid-rich protein like 3	1,21
6451	SH3BGRL	SH3 domain binding glutamic acid-rich protein like	1,21
51247	PAIP2	poly(A) binding protein interacting protein 2	1,21
399491	LOC399491	GPS, PLAT and transmembrane domain-containing protein	1,21
55173	MRPS10	mitochondrial ribosomal protein S10	1,21
326624	RAB37	RAB37, member RAS oncogene family	1,2
64747	MFSD1	major facilitator superfamily domain containing 1	1,2
649181	ILMN_41226	PREDICTED: Homo sapiens hypothetical protein LOC649181 (LOC649181), mRNA.	1,2
3423	IDS	iduronate 2-sulfatase	1,2
3107	HLA-C	major histocompatibility complex, class I, C	1,2
2768	GNA12	guanine nucleotide binding protein (G protein) alpha 12	1,2
6352	CCL5	chemokine (C-C motif) ligand 5	1,2
79038	ZFYVE21	zinc finger, FYVE domain containing 21	1,19
122553	TRAPPC6B	trafficking protein particle complex 6B	1,19
754	PTTG1IP	pituitary tumor-transforming 1 interacting protein	1,19
4698	NDUFA5	NADH dehydrogenase (ubiquinone) 1 alpha subcomplex, 5, 13kDa	1,19
653061	ILMN_40836	PREDICTED: Homo sapiens similar to golgi autoantigen, golgin subfamily a, 8A (LOC653061), mRNA.	1,19
390367	ILMN_36801	PREDICTED: Homo sapiens hypothetical LOC390367 (LOC390367), mRNA.	1,19

50848	F11R	F11 receptor	1,19
27072	VPS41	vacuolar protein sorting 41 homolog (S. cerevisiae)	1,18
989	7.IX	septin 7	1,18
80148	PQLC1	PQ loop repeat containing 1	1,18
5494	PPM1A	protein phosphatase, Mg2+/Mn2+ dependent, 1A	1,18
51177	PLEKHO1	pleckstrin homology domain containing, family O member 1	1,18
29074	MRPL18	mitochondrial ribosomal protein L18	1,18
727909	ILMN_41851	PREDICTED: Homo sapiens similar to Golgin subfamily A member 8-like protein 2, transcript variant 4 (LOC727909), mRNA.	1,18
642489	ILMN_37913	PREDICTED: Homo sapiens similar to FK506-binding protein 1A (LOC642489), mRNA.	1,18
286239	ILMN_39621	PREDICTED: Homo sapiens similar to NAD-dependent malic enzyme, mitochondrial precursor (NAD-ME) (Malic enzyme 2) (LOC286239), mRNA.	1,18
3738	KCNA3	potassium voltage-gated channel, shaker-related subfamily, member 3	1,18
	ILMN_76886	Homo sapiens cDNA FLJ42306 fis, clone TRACH2001646	1,18
9402	GRAP2	GRB2-related adaptor protein 2	1,18
1647	GADD45A	growth arrest and DNA-damage-inducible, alpha	1,18
801	CALM1	calmodulin 1 (phosphorylase kinase, delta)	1,18
79020	C7orf25	chromosome 7 open reading frame 25	1,18
55973	BCAP29	B-cell receptor-associated protein 29	1,18
6487	ST3GAL3	ST3 beta-galactoside alpha-2,3-sialyltransferase 3	1,17
729317	LOC729317	voltage-dependent anion channel 2 pseudogene	1,17
200942	ILMN_6513	Homo sapiens kelch domain containing 8B (KLHDC8B), mRNA.	1,17
527	ATP6V0C	ATPase, H+ transporting, lysosomal 16kDa, V0 subunit c	1,17
55529	TMEM55A	transmembrane protein 55A	1,16
8926	SNURF	SNRPN upstream reading frame	1,16
643287	ILMN_37869	PREDICTED: Homo sapiens similar to prothymosin alpha, transcript variant 1 (LOC643287), mRNA.	1,16
	ILMN_116660	UI-H-BI3-alr-f-05-0-UI.s1 NCI_CGAP_Sub5 Homo sapiens cDNA clone IMAGE:3068313 3, mRNA sequence	1,16
649853	ILMN_168174	Homo sapiens major histocompatibility complex class I HLA-A29.1 (HLA-A29.1), mRNA. XM_001132736 XM_001132742 XM_001132747 XM_001132750 XM_001132753 XM_001132757 XM_001132760 XM_001132769 XM_001132772 XM_001132776 XM_001132779 XM_001132781 XM_001132783 XM_001132785 XM_001132794	1,16
85236	HIST1H2BK	histone cluster 1, H2bk	1,16
8263	F8A1	coagulation factor VIII-associated 1	1,16
65125	WNK1	WNK lysine deficient protein kinase 1	1,15

348235	SKA2	spindle and kinetochore associated complex subunit 2	1,15
646050	LOC646050	axin interactor, dorsalization associated pseudogene	1,15
644336	ILMN_44408	PREDICTED: Homo sapiens similar to SET domain and mariner transposase fusion gene (LOC644336), mRNA.	1,15
96626	LIMS3	LIM and senescent cell antigen-like domains 3	1,15
25923	ATL3	atlastin GTPase 3	1,15
8714	ILMN_8120	Homo sapiens ATP-binding cassette, sub-family C (CFTR/MRP), member 3 (ABCC3), mRNA.	1,15
26469	PTPN18	protein tyrosine phosphatase, non-receptor type 18 (brain-derived)	1,14
5579	PRKCB	protein kinase C, beta	1,14
57092	PCNP	PEST proteolytic signal containing nuclear protein	1,14
8992	ATP6V0E1	ATPase, H+ transporting, lysosomal 9kDa, V0 subunit e1	1,14
5567	PRKACB	protein kinase, cAMP-dependent, catalytic, beta	1,13
646227	LOC646227	cAMP-regulated phosphoprotein 19 pseudogene	1,13
643451	ILMN_31270	PREDICTED: Homo sapiens hypothetical protein LOC643451 (LOC643451), mRNA.	1,13
54435	ILMN_42290	Homo sapiens HLA complex group 4 (HCG4), non-coding RNA.	1,13
2280	FKBP1A	FK506 binding protein 1A, 12kDa	1,13
8832	CD84	CD84 molecule	1,13
50807	ASAP1	ArfGAP with SH3 domain, ankyrin repeat and PH domain 1	1,13
56950	SMYD2	SET and MYND domain containing 2	1,12
326624	ILMN_10097	Homo sapiens RAB37, member RAS oncogene family (RAB37), transcript variant 1, mRNA.	1,12
115294	PCMTD1	protein-L-isoaspartate (D-aspartate) O-methyltransferase domain containing 1	1,12
	ILMN_124830	Homo sapiens cDNA FLJ46527 fis, clone THYMU3034853	1,12
8334	HIST1H2AC	histone cluster 1, H2ac	1,12
55683	KIAA1310	KIAA1310	1,12
83658	DYNLRB1	dynein, light chain, roadblock-type 1	1,12
977	ILMN_44841	Homo sapiens CD151 molecule (Raph blood group) (CD151), transcript variant 2, mRNA.	1,12
10093	ARPC4	actin related protein 2/3 complex, subunit 4, 20kDa	1,12
7534	YWHAZ	tyrosine 3-monooxygenase/tryptophan 5-monooxygenase activation protein, zeta polypeptide	1,11
9948	WDR1	WD repeat domain 1	1,11
127262	TPRG1L	tumor protein p63 regulated 1-like	1,11
55331	ACER3	alkaline ceramidase 3	1,11
400948	ILMN_32897	PREDICTED: Homo sapiens similar to CG33774-PA (LOC400948), mRNA.	1,11
	ILMN_86463	RST15665 Athersys RAGE Library Homo sapiens cDNA, mRNA sequence	1,11
1831	TSC22D3	TSC22 domain family, member 3	1,1

8848	TSC22D1	TSC22 domain family, member 1	1,1
29780	PARVB	parvin, beta	1,1
27020	NPTN	neuroplastin	1,1
51471	NAT8B	N-acetyltransferase 8B (GCN5-related, putative, gene/pseudogene)	1,1
	ILMN_75430	QV4-GN0120-271100-586-d09 GN0120 Homo sapiens cDNA, mRNA sequence	1,1
2533	FYB	FYN binding protein	1,1
1982	EIF4G2	eukaryotic translation initiation factor 4 gamma, 2	1,1
826	CAPNS1	calpain, small subunit 1	1,1
79752	ILMN_23674	Homo sapiens zinc finger, AN1-type domain 1 (ZFAND1), mRNA.	1,09
5870	RAB6A	RAB6A, member RAS oncogene family	1,09
84861	KLHL22	kelch-like 22 (Drosophila)	1,09
	ILMN_88180	ir56a05.y1 HR85 islet Homo sapiens cDNA clone IMAGE:6549201 5, mRNA sequence	1,09
29940	DSE	dermatan sulfate epimerase	1,09
55973	BCAP29	B-cell receptor-associated protein 29	1,09
374618	TEX9	testis expressed 9	1,08
5573	PRKAR1A	protein kinase, cAMP-dependent, regulatory, type I, alpha (tissue specific extinguisher 1)	1,08
4684	NCAM1	neural cell adhesion molecule 1	1,08
3611	ILK	integrin-linked kinase	1,08
	ILMN_130395	DA957648 SPLEN2 Homo sapiens cDNA clone SPLEN2037016 5, mRNA sequence	1,08
369	ARAF	v-raf murine sarcoma 3611 viral oncogene homolog	1,08
8859	STK19	serine/threonine kinase 19	1,07
84236	RHBDD1	rhomboid domain containing 1	1,07
11105	ILMN_5534	Homo sapiens PR domain containing 7 (PRDM7), transcript variant 1, mRNA.	1,07
23608	MKRN1	makorin ring finger protein 1	1,07
57179	KIAA1191	KIAA1191	1,07
	ILMN_93316	Homo sapiens cDNA FLJ13548 fis, clone PLACE1007068	1,07
3005	H1F0	H1 histone family, member 0	1,07
337867	UBAC2	UBA domain containing 2	1,06
7027	TFDP1	transcription factor Dp-1	1,06
10507	SEMA4D	sema domain, immunoglobulin domain (Ig), transmembrane domain (TM) and short cytoplasmic domain, (semaphorin) 4D	1,06
57161	PELI2	pellino homolog 2 (Drosophila)	1,06
5175	PECAM1	platelet/endothelial cell adhesion molecule	1,06

79772	MCTP1	multiple C2 domains, transmembrane 1	1,06
2914	GRM4	glutamate receptor, metabotropic 4	1,06
285527	FRYL	FRY-like	1,06
80204	FBXO11	F-box protein 11	1,06
116496	FAM129A	family with sequence similarity 129, member A	1,06
1727	CYB5R3	cytochrome b5 reductase 3	1,06
51582	AZIN1	antizyme inhibitor 1	1,06
51382	ATP6V1D	ATPase, H+ transporting, lysosomal 34kDa, V1 subunit D	1,06
2534	FYN	FYN oncogene related to SRC, FGR, YES	1,05
440915	POTEKP	POTE ankyrin domain family, member K, pseudogene	1,05
56990	CDC42SE2	CDC42 small effector 2	1,05
408	ARRB1	arrestin, beta 1	1,05
51094	ADIPOR1	adiponectin receptor 1	1,05
127933	UHMK1	U2AF homology motif (UHM) kinase 1	1,04
153339	TMEM167A	transmembrane protein 167A	1,04
8780	RIOK3	RIO kinase 3 (yeast)	1,04
5294	ILMN_7736	Homo sapiens phosphoinositide-3-kinase, catalytic, gamma polypeptide (PIK3CG), mRNA.	1,04
11235	PDCD10	programmed cell death 10	1,04
649214	ILMN_33279	PREDICTED: Homo sapiens similar to High mobility group protein 1-like 10 (HMG-1L10), transcript variant 4 (LOC649214), mRNA.	1,04
25932	CLIC4	chloride intracellular channel 4	1,04
896	CCND3	cyclin D3	1,04
400804	ILMN_29977	Homo sapiens chromosome 1 open reading frame 140 (C1orf140), mRNA.	1,04
223	ALDH9A1	aldehyde dehydrogenase 9 family, member A1	1,04
157680	VPS13B	vacuolar protein sorting 13 homolog B (yeast)	1,03
56257	MEPCE	methylphosphate capping enzyme	1,03
646463	ILMN_38228	PREDICTED: Homo sapiens similar to Ubiquitin-conjugating enzyme E2 H (Ubiquitin-protein ligase H) (Ubiquitin carrier protein H) (UBCH2) (E2-20K) (LOC646463), mRNA.	1,03
9002	ILMN_43570	Homo sapiens coagulation factor II (thrombin) receptor-like 3 (F2RL3), mRNA.	1,03
10095	ARPC1B	actin related protein 2/3 complex, subunit 1B, 41kDa	1,03
54470	ILMN_6931	Homo sapiens armadillo repeat containing, X-linked 6 (ARMCX6), transcript variant 1, mRNA.	1,03
649395	LOC649395	tyrosine 3-monooxygenase/tryptophan 5-monooxygenase activation protein, epsilon polypeptide pseudogene	1,02
4673	NAP1L1	nucleosome assembly protein 1-like 1	1,02

652489	ILMN_36884	PREDICTED: Homo sapiens similar to SMT3 suppressor of mif two 3 homolog 2 (LOC652489), mRNA.	1,02
3300	DNAJB2	DnaJ (Hsp40) homolog, subfamily B, member 2	1,02
54622	ARL15	ADP-ribosylation factor-like 15	1,02
9465	AKAP7	A kinase (PRKA) anchor protein 7	1,02
7739	ZNF185	zinc finger protein 185 (LIM domain)	1,01
5908	ILMN_166610	Homo sapiens RAP1B, member of RAS oncogene family (RAP1B), transcript variant 1, mRNA.	1,01
5257	PHKB	phosphorylase kinase, beta	1,01
3725	JUN	jun proto-oncogene	1,01
3636	INPPL1	inositol polyphosphate phosphatase-like 1	1,01
85236	ILMN_17974	Homo sapiens histone cluster 1, H2bk (HIST1H2BK), mRNA.	1,01
64423	INF2	inverted formin, FH2 and WH2 domain containing	1,01
665	BNIP3L	BCL2/adenovirus E1B 19kDa interacting protein 3-like	1,01
7360	ILMN_24416	Homo sapiens UDP-glucose pyrophosphorylase 2 (UGP2), transcript variant 1, mRNA.	1
57187	THOC2	THO complex 2	1
3516	RBPJ	recombination signal binding protein for immunoglobulin kappa J region	1
5567	PRKACB	protein kinase, cAMP-dependent, catalytic, beta	1
1355	ILMN_14561	Homo sapiens COX15 homolog, cytochrome c oxidase assembly protein (yeast) (COX15), nuclear gene encoding mitochondrial protein, transcript variant 1, mRNA.	1
10282	BET1	blocked early in transport 1 homolog (S. cerevisiae)	1
10632	ATP5L	ATP synthase, H+ transporting, mitochondrial Fo complex, subunit G	1
140885	SIRPA	signal-regulatory protein alpha	-1
10125	RASGRP1	RAS guanyl releasing protein 1 (calcium and DAG-regulated)	-1
22827	PUF60	poly-U binding splicing factor 60KDa	-1
55035	NOL8	nucleolar protein 8	-1
4691	NCL	nucleolin	-1
91663	MYADM	myeloid-associated differentiation marker	-1
	ILMN_136538	DB027523 TESTI2 Homo sapiens cDNA clone TESTI2010045 5, mRNA sequence	-1
11033	ADAP1	ArfGAP with dual PH domains 1	-1
55101	ILMN_12644	Homo sapiens ATP5S-like (ATP5SL), mRNA.	-1
79852	EPHX3	epoxide hydrolase 3	-1
92912	UBE2Q2	ubiquitin-conjugating enzyme E2Q family member 2	-1,01
7327	UBE2G2	ubiquitin-conjugating enzyme E2G 2	-1,01
11262	SP140	SP140 nuclear body protein	-1,01

6427	SRSF2	serine/arginine-rich splicing factor 2	-1,01
9045	RPL14	ribosomal protein L14	-1,01
5435	POLR2F	polymerase (RNA) II (DNA directed) polypeptide F	-1,01
4688	ILMN_8146	Homo sapiens neutrophil cytosolic factor 2 (65kDa, chronic granulomatous disease, autosomal 2) (NCF2), mRNA.	-1,01
439949	LOC439949	hypothetical LOC439949	-1,01
8761	PABPC4	poly(A) binding protein, cytoplasmic 4 (inducible form)	-1,01
	ILMN_119133	wb37h11.x1 NCI_CGAP_GC6 Homo sapiens cDNA clone IMAGE:2307909 3, mRNA sequence	-1,01
27069	GHITM	growth hormone inducible transmembrane protein	-1,01
10480	EIF3M	eukaryotic translation initiation factor 3, subunit M	-1,01
728358	ILMN_11220	Homo sapiens defensin, alpha 1B (DEFA1B), mRNA.	-1,01
972	CD74	CD74 molecule, major histocompatibility complex, class II invariant chain	-1,01
25798	BRI3	brain protein I3	-1,01
6856	SYPL1	synaptophysin-like 1	-1,02
8675	STX16	syntaxin 16	-1,02
728609	ILMN_175112	Homo sapiens succinate dehydrogenase complex, subunit A, flavoprotein pseudogene 3 (SDHAP3) on chromosome 5.	-1,02
6222	RPS18	ribosomal protein S18	-1,02
6204	RPS10	ribosomal protein S10	-1,02
644029	ILMN_34790	PREDICTED: Homo sapiens similar to 60S ribosomal protein L7a (LOC644029), mRNA.	-1,02
643313	ILMN_44117	PREDICTED: Homo sapiens similar to hypothetical protein LOC284701, transcript variant 1 (LOC643313), mRNA.	-1,02
200030	ILMN_10536	Homo sapiens neuroblastoma breakpoint family, member 11-like (LOC200030), mRNA.	-1,02
	ILMN_104476	zj45e10.s1 Soares_fetal_liver_spleen_1NFLS_S1 Homo sapiens cDNA clone IMAGE:453258 3, mRNA sequence	-1,02
10818	FRS2	fibroblast growth factor receptor substrate 2	-1,02
116988	AGAP3	ArfGAP with GTPase domain, ankyrin repeat and PH domain 3	-1,02
829	CAPZA1	capping protein (actin filament) muscle Z-line, alpha 1	-1,02
694	BTG1	B-cell translocation gene 1, anti-proliferative	-1,02
3431	SP110	SP110 nuclear body protein	-1,03
644131	CCT8P1	chaperonin containing TCP1, subunit 8 (theta) pseudogene 1	-1,03
6161	RPL32	ribosomal protein L32	-1,03
1431	CS	citrate synthase	-1,03
6767	ST13	suppression of tumorigenicity 13 (colon carcinoma) (Hsp70 interacting protein)	-1,04
6342	SCP2	sterol carrier protein 2	-1,04
5089	ILMN_7500	Homo sapiens pre-B-cell leukemia homeobox 2 (PBX2), mRNA.	-1,04
6885	MAP3K7	mitogen-activated protein kinase kinase kinase 7	-1,04

653559	ILMN_43625	PREDICTED: Homo sapiens similar to Rho-associated protein kinase 1 (Rho-associated, coiled-coil containing protein kinase 1) (p160 ROCK-1) (p160ROCK) (LOC653559), mRNA.	-1,04
646144	ILMN_45842	PREDICTED: Homo sapiens similar to tousled-like kinase 2, transcript variant 2 (LOC646144), mRNA.	-1,04
643920	ILMN_39946	PREDICTED: Homo sapiens region containing similar to Williams Beuren syndrome chromosome region 19; hypothetical LOC441257, transcript variant 2 (LOC643920), mRNA.	-1,04
642934	ILMN_33728	PREDICTED: Homo sapiens hypothetical LOC642934 (LOC642934), mRNA.	-1,04
11100	HNRNPUL1	heterogeneous nuclear ribonucleoprotein U-like 1	-1,04
2949	GSTM5	glutathione S-transferase mu 5	-1,04
349152	ILMN_15856	Homo sapiens dpy-19-like 2 pseudogene 2 (C. elegans) (DPY19L2P2), non-coding RNA.	-1,04
53981	ILMN_181110	Homo sapiens cleavage and polyadenylation specific factor 2, 100kDa (CPSF2), mRNA.	-1,04
1236	CCR7	chemokine (C-C motif) receptor 7	-1,04
303	ANXA2P1	annexin A2 pseudogene 1	-1,04
3268	AGFG2	ArfGAP with FG repeats 2	-1,04
54915	YTHDF1	YTH domain family, member 1	-1,05
25777	SUN2	Sad1 and UNC84 domain containing 2	-1,05
29108	PYCARD	PYD and CARD domain containing	-1,05
5788	PTPRC	protein tyrosine phosphatase, receptor type, C	-1,05
5687	PSMA6	proteasome (prosome, macropain) subunit, alpha type, 6	-1,05
90007	MIDN	midnolin	-1,05
79863	RBFA	ribosome binding factor A (putative)	-1,05
121274	ZNF641	zinc finger protein 641	-1,06
6161	RPL32	ribosomal protein L32	-1,06
9991	ROD1	ROD1 regulator of differentiation 1 (S. pombe)	-1,06
57665	RDH14	retinol dehydrogenase 14 (all-trans/9-cis/11-cis)	-1,06
3804	KIR2DL3	killer cell immunoglobulin-like receptor, two domains, long cytoplasmic tail, 3	-1,06
9551	ATP5J2	ATP synthase, H+ transporting, mitochondrial Fo complex, subunit F2	-1,06
11040	ILMN_27932	Homo sapiens pim-2 oncogene (PIM2), mRNA.	-1,07
7940	LST1	leukocyte specific transcript 1	-1,07
645489	ILMN_41776	PREDICTED: Homo sapiens hypothetical protein LOC645489 (LOC645489), mRNA.	-1,07
3805	ILMN_16588	Homo sapiens killer cell immunoglobulin-like receptor, two domains, long cytoplasmic tail, 4 (KIR2DL4), mRNA.	-1,07
1370	ILMN_42585	PREDICTED: Homo sapiens carboxypeptidase N, polypeptide 2, 83kD (CPN2), mRNA.	-1,07
285382	C3orf70	chromosome 3 open reading frame 70	-1,07
8725	C19orf2	chromosome 19 open reading frame 2	-1,07

83719	YPEL3	yippee-like 3 (Drosophila)	-1,08
255758	TCTEX1D2	Tctex1 domain containing 2	-1,08
7979	SHFM1	split hand/foot malformation (ectrodactyly) type 1	-1,08
51316	PLAC8	placenta-specific 8	-1,08
402110	ILMN_21477	Homo sapiens hypothetical LOC402110 (LOC402110), mRNA.	-1,08
158345	ILMN_36732	PREDICTED: Homo sapiens similar to ribosomal protein L4 (LOC158345), mRNA.	-1,08
123876	ILMN_25856	Homo sapiens hypothetical protein LOC123876 (LOC123876), mRNA.	-1,08
9815	GIT2	G protein-coupled receptor kinase interacting ArfGAP 2	-1,08
1668	ILMN_11220	Homo sapiens defensin, alpha 3, neutrophil-specific (DEFA3), mRNA.	-1,08
841	CASP8	caspase 8, apoptosis-related cysteine peptidase	-1,08
7430	EZR	ezrin	-1,09
56910	STARD7	StAR-related lipid transfer (START) domain containing 7	-1,09
10181	RBM5	RNA binding motif protein 5	-1,09
440345	ILMN_43785	PREDICTED: Homo sapiens hypothetical protein LOC440345, transcript variant 5 (LOC440345), mRNA.	-1,09
168537	GIMAP7	GTPase, IMAP family member 7	-1,09
57609	DIP2B	DIP2 disco-interacting protein 2 homolog B (Drosophila)	-1,09
1667	ILMN_29692	Homo sapiens defensin, alpha 1 (DEFA1), mRNA.	-1,09
11325	DDX42	DEAD (Asp-Glu-Ala-Asp) box polypeptide 42	-1,09
521	ATP5I	ATP synthase, H+ transporting, mitochondrial Fo complex, subunit E	-1,09
10476	ATP5H	ATP synthase, H+ transporting, mitochondrial Fo complex, subunit d	-1,09
644037	HNRNPA1P12	heterogeneous nuclear ribonucleoprotein A1 pseudogene 12	-1,1
	ILMN_115736	HSC1JB062 normalized infant brain cDNA Homo sapiens cDNA clone c-1jb06 3, mRNA sequence	-1,1
8893	EIF2B5	eukaryotic translation initiation factor 2B, subunit 5 epsilon, 82kDa	-1,1
919	CD247	CD247 molecule	-1,1
6232	RPS27	ribosomal protein S27	-1,11
6129	RPL7	ribosomal protein L7	-1,11
4736	RPL10A	ribosomal protein L10a	-1,11
6129	RPL7	ribosomal protein L7	-1,11
645385	ILMN_33113	PREDICTED: Homo sapiens similar to heterogeneous nuclear ribonucleoprotein A1 (LOC645385), mRNA.	-1,11
6129	RPL7	ribosomal protein L7	-1,11
10437	IFI30	interferon, gamma-inducible protein 30	-1,11
115362	GBP5	guanylate binding protein 5	-1,11
23401	FRAT2	frequently rearranged in advanced T-cell lymphomas 2	-1,11

25940	FAM98A	family with sequence similarity 98, member A	-1,11
10658	ILMN_10496	Homo sapiens CUG triplet repeat, RNA binding protein 1 (CUGBP1), transcript variant 2, mRNA.	-1,11
150684	COMMD1	copper metabolism (Murr1) domain containing 1	-1,11
7576	ILMN_8130	Homo sapiens zinc finger protein 28 (ZNF28), mRNA.	-1,12
4046	LSP1	lymphocyte-specific protein 1	-1,12
145165	ST13P4	suppression of tumorigenicity 13 (colon carcinoma) (Hsp70 interacting protein) pseudogene 4	-1,12
9337	CNOT8	CCR4-NOT transcription complex, subunit 8	-1,12
51385	ZNF589	zinc finger protein 589	-1,13
7127	ILMN_16372	Homo sapiens tumor necrosis factor, alpha-induced protein 2 (TNFAIP2), mRNA.	-1,13
441502	RPS26P11	ribosomal protein S26 pseudogene 11	-1,13
643310	ST13P18	suppression of tumorigenicity 13 (colon carcinoma) (Hsp70 interacting protein) pseudogene 18	-1,13
10542	HBXIP	hepatitis B virus x interacting protein	-1,13
7555	CNBP	CCHC-type zinc finger, nucleic acid binding protein	-1,13
200316	ILMN_1659	Homo sapiens apolipoprotein B mRNA editing enzyme, catalytic polypeptide-like 3F (APOBEC3F), transcript variant 1, mRNA.	-1,13
303	ANXA2P1	annexin A2 pseudogene 1	-1,13
7267	TTC3	tetratricopeptide repeat domain 3	-1,14
6166	RPL36AL	ribosomal protein L36a-like	-1,14
118433	ILMN_3185	Homo sapiens ribosomal protein L23a pseudogene 7 (RPL23AP7) on chromosome 2.	-1,14
8241	RBM10	RNA binding motif protein 10	-1,14
8566	PDXK	pyridoxal (pyridoxine, vitamin B6) kinase	-1,14
3385	ICAM3	intercellular adhesion molecule 3	-1,14
50809	HP1BP3	heterochromatin protein 1, binding protein 3	-1,15
8338	HIST2H2AC	histone cluster 2, H2ac	-1,15
55140	ELP3	elongation protein 3 homolog (S. cerevisiae)	-1,15
1933	EEF1B2	eukaryotic translation elongation factor 1 beta 2	-1,15
9170	LPAR2	lysophosphatidic acid receptor 2	-1,15
10521	DDX17	DEAD (Asp-Glu-Ala-Asp) box polypeptide 17	-1,15
9184	BUB3	budding uninhibited by benzimidazoles 3 homolog (yeast)	-1,15
8635	RNASET2	ribonuclease T2	-1,16
3689	ILMN_138267	Homo sapiens integrin, beta 2 (antigen CD18 (p95), lymphocyte function-associated antigen 1; macrophage antigen 1 (mac-1) beta subunit) (ITGB2), mRNA.	-1,16
1642	DDB1	damage-specific DNA binding protein 1, 127kDa	-1,16

55196	C12orf35	chromosome 12 open reading frame 35	-1,16
23753	SDF2L1	stromal cell-derived factor 2-like 1	-1,17
79671	NLRX1	NLR family member X1	-1,17
728554	ILMN_137226	PREDICTED: Homo sapiens similar to THO complex 3 (LOC728554), mRNA.	-1,17
389435	ILMN_30712	Homo sapiens hCG21078 (LOC389435), mRNA.	-1,17
85376	ILMN_37441	PREDICTED: Homo sapiens KIAA1666 protein (KIAA1666), mRNA.	-1,17
2289	FKBP5	FK506 binding protein 5	-1,17
8721	EDF1	endothelial differentiation-related factor 1	-1,17
54541	DDIT4	DNA-damage-inducible transcript 4	-1,17
648237	ILMN_40504	PREDICTED: Homo sapiens similar to 60S ribosomal protein L10 (QM protein) (Tumor suppressor QM) (Laminin receptor homolog) (LOC648237), mRNA.	-1,18
3482	IGF2R	insulin-like growth factor 2 receptor	-1,18
868	CBLB	Cas-Br-M (murine) ecotropic retroviral transforming sequence b	-1,18
148789	B3GALNT2	beta-1,3-N-acetylgalactosaminyltransferase 2	-1,18
120425	AMICA1	adhesion molecule, interacts with CXADR antigen 1	-1,18
8795	TNFRSF10B	tumor necrosis factor receptor superfamily, member 10b	-1,19
6778	STAT6	signal transducer and activator of transcription 6, interleukin-4 induced	-1,19
80143	SIKE1	suppressor of IKBKE 1	-1,19
5871	MAP4K2	mitogen-activated protein kinase kinase kinase kinase 2	-1,19
728734	ILMN_12371	PREDICTED: Homo sapiens similar to kidney-specific protein (KS), transcript variant 1 (LOC728734), mRNA.	-1,19
6234	RPS28	ribosomal protein S28	-1,19
613037	LOC613037	nuclear pore complex interacting protein pseudogene	-1,19
146053	ILMN_40640	PREDICTED: Homo sapiens similar to ribosomal protein S3a, transcript variant 4 (LOC146053), mRNA.	-1,19
3067	HDC	histidine decarboxylase	-1,19
689	BTF3	basic transcription factor 3	-1,19
9349	RPL23	ribosomal protein L23	-1,2
5788	PTPRC	protein tyrosine phosphatase, receptor type, C	-1,2
4332	MNDA	myeloid cell nuclear differentiation antigen	-1,2
645688	ILMN_35293	PREDICTED: Homo sapiens similar to 60S ribosomal protein L12 (LOC645688), mRNA.	-1,2
3303	HSPA1A	heat shock 70kDa protein 1A	-1,2
	ILMN_127075	BX096001 Soares fetal liver spleen 1NFLS Homo sapiens cDNA clone IMAGp998O09393, mRNA sequence	-1,2
2948	ILMN_4003	Homo sapiens glutathione S-transferase M4 (GSTM4), transcript variant 2, mRNA.	-1,2
89853	FAM125B	family with sequence similarity 125, member B	-1,2

23468	CBX5	chromobox homolog 5	-1,2
26136	TES	testis derived transcript (3 LIM domains)	-1,21
6132	RPL8	ribosomal protein L8	-1,21
6128	RPL6	ribosomal protein L6	-1,21
11159	RABL2A	RAB, member of RAS oncogene family-like 2A	-1,21
4809	NHP2L1	NHP2 non-histone chromosome protein 2-like 1 (S. cerevisiae)	-1,21
645899	ILMN_42597	PREDICTED: Homo sapiens similar to 40S ribosomal protein S28 (LOC645899), mRNA.	-1,21
8665	EIF3F	eukaryotic translation initiation factor 3, subunit F	-1,21
221960	C7orf28B	chromosome 7 open reading frame 28B	-1,21
596	BCL2	B-cell CLL/lymphoma 2	-1,21
2113	ETS1	v-ets erythroblastosis virus E26 oncogene homolog 1 (avian)	-1,22
64744	SMAP2	small ArfGAP2	-1,23
6280	S100A9	S100 calcium binding protein A9	-1,23
6205	RPS11	ribosomal protein S11	-1,23
5547	PRCP	prolylcarboxypeptidase (angiotensinase C)	-1,23
79960	PHF17	PHD finger protein 17	-1,23
9045	RPL14	ribosomal protein L14	-1,23
646350	ILMN_39634	PREDICTED: Homo sapiens similar to 60S ribosomal protein L39 (LOC646350), mRNA.	-1,23
10261	IGSF6	immunoglobulin superfamily, member 6	-1,23
142	ILMN_3056	Homo sapiens poly (ADP-ribose) polymerase family, member 1 (PARP1), mRNA.	-1,24
6228	RPS23	ribosomal protein S23	-1,24
2483	FRG1	FSHD region gene 1	-1,24
3936	LCP1	lymphocyte cytosolic protein 1 (L-plastin)	-1,24
3329	HSPD1	heat shock 60kDa protein 1 (chaperonin)	-1,24
3059	HCLS1	hematopoietic cell-specific Lyn substrate 1	-1,24
2091	FBL	fibrillarin	-1,24
689	BTF3	basic transcription factor 3	-1,24
9551	ATP5J2	ATP synthase, H+ transporting, mitochondrial Fo complex, subunit F2	-1,24
64333	ARHGAP9	Rho GTPase activating protein 9	-1,24
57050	UTP3	UTP3, small subunit (SSU) processome component, homolog (S. cerevisiae)	-1,25
81929	SEH1L	SEH1-like (S. cerevisiae)	-1,25
6230	RPS25	ribosomal protein S25	-1,25
284194	LGALS9B	lectin, galactoside-binding, soluble, 9B	-1,25

51759	C9orf78	chromosome 9 open reading frame 78	-1,25
10961	ERP29	endoplasmic reticulum protein 29	-1,25
6169	RPL38	ribosomal protein L38	-1,26
4245	MGAT1	mannosyl (alpha-1,3-)-glycoprotein beta-1,2-N-acetylglucosaminyltransferase	-1,26
8366	HIST1H4B	histone cluster 1, H4b	-1,26
23167	EFR3A	EFR3 homolog A (S. cerevisiae)	-1,26
115761	ARL11	ADP-ribosylation factor-like 11	-1,26
388078	ILMN_31332	PREDICTED: Homo sapiens V-set and immunoglobulin domain containing 6 (VSIG6), mRNA.	-1,27
11066	SNRNP35	small nuclear ribonucleoprotein 35kDa (U11/U12)	-1,27
5859	QARS	glutaminyl-tRNA synthetase	-1,27
653232	ILMN_31434	PREDICTED: Homo sapiens hypothetical LOC653232, transcript variant 4 (LOC653232), mRNA.	-1,27
644131	CCT8P1	chaperonin containing TCP1, subunit 8 (theta) pseudogene 1	-1,27
9807	IP6K1	inositol hexakisphosphate kinase 1	-1,27
6742	SSBP1	single-stranded DNA binding protein 1	-1,28
6223	RPS19	ribosomal protein S19	-1,28
642333	ILMN_40016	PREDICTED: Homo sapiens similar to M-phase phosphoprotein, mpp8 (LOC642333), mRNA.	-1,28
3727	ILMN_29325	Homo sapiens jun D proto-oncogene (JUND), mRNA.	-1,28
7416	VDAC1	voltage-dependent anion channel 1	-1,29
26019	UPF2	UPF2 regulator of nonsense transcripts homolog (yeast)	-1,29
6208	RPS14	ribosomal protein S14	-1,29
645683	RPL13AP3	ribosomal protein L13a pseudogene 3	-1,29
54832	VPS13C	vacuolar protein sorting 13 homolog C (S. cerevisiae)	-1,3
51667	NUB1	negative regulator of ubiquitin-like proteins 1	-1,3
642817	ILMN_46700	PREDICTED: Homo sapiens hypothetical LOC642817 (LOC642817), mRNA.	-1,3
	ILMN_130610	DB337110 TESTI2 Homo sapiens cDNA clone TESTI2016331 3, mRNA sequence	-1,3
22918	CD93	CD93 molecule	-1,3
94104	GCFC1	GC-rich sequence DNA-binding factor 1	-1,3
55729	ATF7IP	activating transcription factor 7 interacting protein	-1,3
7175	TPR	translocated promoter region (to activated MET oncogene)	-1,31
26207	PITPNC1	phosphatidylinositol transfer protein, cytoplasmic 1	-1,31
23117	ILMN_44181	PREDICTED: Homo sapiens KIAA0220-like protein, transcript variant 16 (LOC23117), mRNA.	-1,31
10922	FASTK	Fas-activated serine/threonine kinase	-1,31
51335	NGRN	neugrin, neurite outgrowth associated	-1,32

374395	TMEM179B	transmembrane protein 179B	-1,32
11158	RABL2B	RAB, member of RAS oncogene family-like 2B	-1,33
64976	MRPL40	mitochondrial ribosomal protein L40	-1,33
606724	ILMN_9207	Homo sapiens coronin, actin binding protein, 1A pseudogene (LOC606724), non-coding RNA.	-1,33
6230	RPS25	ribosomal protein S25	-1,33
10412	NSA2	NSA2 ribosome biogenesis homolog (S. cerevisiae)	-1,34
728358	ILMN_176067	Homo sapiens defensin, alpha 1B (DEFA1B), mRNA.	-1,34
302	ANXA2	annexin A2	-1,34
85464	SSH2	slingshot homolog 2 (Drosophila)	-1,35
400156	ILMN_38819	PREDICTED: Homo sapiens 40S ribosomal protein S26-like (RPS26L), misc RNA.	-1,35
6217	RPS16	ribosomal protein S16	-1,35
6154	RPL26	ribosomal protein L26	-1,35
126382	NR2C2AP	nuclear receptor 2C2-associated protein	-1,35
4728	NDUFS8	NADH dehydrogenase (ubiquinone) Fe-S protein 8, 23kDa (NADH-coenzyme Q reductase)	-1,35
402694	ILMN_32451	Homo sapiens similar to ribosomal protein L15 (LOC402694), mRNA.	-1,35
2802	GOLGA3	golgin A3	-1,35
50854	C6orf48	chromosome 6 open reading frame 48	-1,35
9984	THOC1	THO complex 1	-1,36
4689	NCF4	neutrophil cytosolic factor 4, 40kDa	-1,36
653232	ILMN_41130	PREDICTED: Homo sapiens hypothetical LOC653232, transcript variant 4 (LOC653232), mRNA.	-1,36
6187	RPS2	ribosomal protein S2	-1,36
7462	LAT2	linker for activation of T cells family, member 2	-1,36
5694	PSMB6	proteasome (prosome, macropain) subunit, beta type, 6	-1,37
648622	ILMN_42936	PREDICTED: Homo sapiens similar to ribosomal protein S27 (LOC648622), mRNA.	-1,37
3128	HLA-DRB6	major histocompatibility complex, class II, DR beta 6 (pseudogene)	-1,37
1975	EIF4B	eukaryotic translation initiation factor 4B	-1,37
90416	C15orf57	chromosome 15 open reading frame 57	-1,37
80335	WDR82	WD repeat domain 82	-1,38
6189	RPS3A	ribosomal protein S3A	-1,38
59339	PLEKHA2	pleckstrin homology domain containing, family A (phosphoinositide binding specific) member 2	-1,38
649548	ILMN_31442	PREDICTED: Homo sapiens similar to 60S ribosomal protein L32 (LOC649548), mRNA.	-1,38
	ILMN_108776	od80c09.s1 NCI_CGAP_Ov2 Homo sapiens cDNA clone IMAGE:1374256, mRNA sequence	-1,38
84881	RPUSD4	RNA pseudouridylate synthase domain containing 4	-1,39

27303	RBMS3	RNA binding motif, single stranded interacting protein 3	-1,39
55239	ILMN_16561	Homo sapiens 2-oxoglutarate and iron-dependent oxygenase domain containing 1 (OGFOD1), mRNA.	-1,39
4144	MAT2A	methionine adenosyltransferase II, alpha	-1,39
23405	DICER1	dicer 1, ribonuclease type III	-1,39
83880	ILMN_9478	Homo sapiens IFP38 (IFP38), mRNA.	-1,39
1933	ILMN_4325	Homo sapiens eukaryotic translation elongation factor 1 beta 2 (EEF1B2), transcript variant 3, mRNA.	-1,39
5898	RALA	v-ral simian leukemia viral oncogene homolog A (ras related)	-1,4
6146	RPL22	ribosomal protein L22	-1,4
6187	RPS2	ribosomal protein S2	-1,4
2197	FAU	Finkel-Biskis-Reilly murine sarcoma virus (FBR-MuSV) ubiquitously expressed	-1,4
1973	EIF4A1	eukaryotic translation initiation factor 4A1	-1,4
2181	ACSL3	acyl-CoA synthetase long-chain family member 3	-1,4
7247	TSN	translin	-1,41
5695	PSMB7	proteasome (prosome, macropain) subunit, beta type, 7	-1,41
5336	PLCG2	phospholipase C, gamma 2 (phosphatidylinositol-specific)	-1,41
23484	LEPROTL1	leptin receptor overlapping transcript-like 1	-1,41
56683	C21orf59	chromosome 21 open reading frame 59	-1,41
526	ATP6V1B2	ATPase, H+ transporting, lysosomal 56/58kDa, V1 subunit B2	-1,41
6625	SNRNP70	small nuclear ribonucleoprotein 70kDa (U1)	-1,42
9360	PPIG	peptidylprolyl isomerase G (cyclophilin G)	-1,42
6134	RPL10	ribosomal protein L10	-1,42
441032	ILMN_46443	PREDICTED: Homo sapiens similar to eukaryotic translation elongation factor 1 alpha 1, transcript variant 11 (LOC441032), mRNA.	-1,42
	ILMN_114404	yu05d06.s1 Soares fetal liver spleen 1NFLS Homo sapiens cDNA clone IMAGE:232907 3, mRNA sequence	-1,42
1629	ILMN_124019	Homo sapiens dihydrolipoamide branched chain transacylase E2 (DBT), nuclear gene encoding mitochondrial protein, mRNA.	-1,42
6136	RPL12	ribosomal protein L12	-1,42
91746	YTHDC1	YTH domain containing 1	-1,43
6139	RPL17	ribosomal protein L17	-1,43
441377	ILMN_31681	PREDICTED: Homo sapiens similar to 40S ribosomal protein S26 (LOC441377), mRNA.	-1,43
402251	ILMN_45342	PREDICTED: Homo sapiens similar to eukaryotic translation elongation factor 1 alpha 2 (LOC402251), mRNA.	-1,43
3184	HNRNPD	heterogeneous nuclear ribonucleoprotein D (AU-rich element RNA binding protein 1, 37kDa)	-1,43
168537	GIMAP7	GTPase, IMAP family member 7	-1,43

7705	ZNF146	zinc finger protein 146	-1,44
26801	SNORD48	small nucleolar RNA, C/D box 48	-1,44
22828	SCAF8	SR-related CTD-associated factor 8	-1,44
5621	PRNP	prion protein	-1,44
26059	ERC2	ELKS/RAB6-interacting/CAST family member 2	-1,44
6206	RPS12	ribosomal protein S12	-1,45
81576	CCDC130	coiled-coil domain containing 130	-1,45
6159	RPL29	ribosomal protein L29	-1,46
1387	CREBBP	CREB binding protein	-1,46
328	APEX1	APEX nuclease (multifunctional DNA repair enzyme) 1	-1,46
7529	YWHAB	tyrosine 3-monooxygenase/tryptophan 5-monooxygenase activation protein, beta polypeptide	-1,47
6193	RPS5	ribosomal protein S5	-1,47
149628	PYHIN1	pyrin and HIN domain family, member 1	-1,47
64981	MRPL34	mitochondrial ribosomal protein L34	-1,47
402057	ILMN_40648	Homo sapiens similar to 40S ribosomal protein S17 (LOC402057), mRNA.	-1,47
6128	RPL6	ribosomal protein L6	-1,47
3122	HLA-DRA	major histocompatibility complex, class II, DR alpha	-1,47
56616	DIABLO	diablo, IAP-binding mitochondrial protein	-1,47
28951	TRIB2	tribbles homolog 2 (Drosophila)	-1,48
5018	OXA1L	oxidase (cytochrome c) assembly 1-like	-1,48
3181	HNRNPA2B1	heterogeneous nuclear ribonucleoprotein A2/B1	-1,48
552891	ILMN_29106	Homo sapiens DNAJC25-GNG10 readthrough (DNAJC25-GNG10), mRNA.	-1,48
81609	SNX27	sorting nexin family member 27	-1,49
6191	RPS4X	ribosomal protein S4, X-linked	-1,49
6176	RPLP1	ribosomal protein, large, P1	-1,49
6136	RPL12	ribosomal protein L12	-1,49
80223	RAB11FIP1	RAB11 family interacting protein 1 (class I)	-1,49
5777	PTPN6	protein tyrosine phosphatase, non-receptor type 6	-1,49
650298	ILMN_42423	PREDICTED: Homo sapiens similar to 40S ribosomal protein S26 (LOC650298), mRNA.	-1,49
391656	ILMN_43142	PREDICTED: Homo sapiens similar to hCG1640454 (LOC391656), mRNA.	-1,49
1933	EEF1B2	eukaryotic translation elongation factor 1 beta 2	-1,49
8751	ADAM15	ADAM metallopeptidase domain 15	-1,49
84619	ZGPAT	zinc finger, CCCH-type with G patch domain	-1,5

7454	WAS	Wiskott-Aldrich syndrome (eczema-thrombocytopenia)	-1,5
6208	RPS14	ribosomal protein S14	-1,5
9991	ROD1	ROD1 regulator of differentiation 1 (S. pombe)	-1,5
10608	MXD4	MAX dimerization protein 4	-1,5
4050	LTB	lymphotoxin beta (TNF superfamily, member 3)	-1,5
651894	ILMN_34885	PREDICTED: Homo sapiens similar to ribosomal protein S12 (LOC651894), mRNA.	-1,5
6128	RPL6	ribosomal protein L6	-1,5
284821	ILMN_43560	PREDICTED: Homo sapiens similar to ribosomal protein L13a (LOC284821), mRNA.	-1,5
6231	RPS26	ribosomal protein S26	-1,51
441246	ILMN_31934	PREDICTED: Homo sapiens similar to 60S ribosomal protein L35, transcript variant 1 (LOC441246), mRNA.	-1,51
3117	ILMN_41173	PREDICTED: Homo sapiens major histocompatibility complex, class II, DQ alpha 1, transcript variant 10 (HLA-DQA1), mRNA.	-1,51
10694	CCT8	chaperonin containing TCP1, subunit 8 (theta)	-1,51
23420	ILMN_1736	Homo sapiens NODAL modulator 1 (NOMO1), mRNA.	-1,53
4170	MCL1	myeloid cell leukemia sequence 1 (BCL2-related)	-1,53
1936	EEF1D	eukaryotic translation elongation factor 1 delta (guanine nucleotide exchange protein)	-1,53
6232	RPS27	ribosomal protein S27	-1,54
648000	ILMN_32447	PREDICTED: Homo sapiens similar to 60S ribosomal protein L7, transcript variant 1 (LOC648000), mRNA.	-1,54
6134	RPL10	ribosomal protein L10	-1,54
3122	HLA-DRA	major histocompatibility complex, class II, DR alpha	-1,54
972	CD74	CD74 molecule, major histocompatibility complex, class II invariant chain	-1,54
6235	RPS29	ribosomal protein S29	-1,55
653773	ILMN_44662	PREDICTED: Homo sapiens similar to ribosomal protein L31 (LOC653773), mRNA.	-1,55
54543	TOMM7	translocase of outer mitochondrial membrane 7 homolog (yeast)	-1,56
152007	GLIPR2	GLI pathogenesis-related 2	-1,56
112398	EGLN2	egl nine homolog 2 (C. elegans)	-1,56
115416	C7orf30	chromosome 7 open reading frame 30	-1,56
645	BLVRB	biliverdin reductase B (flavin reductase (NADPH))	-1,56
6230	RPS25	ribosomal protein S25	-1,57
5547	PRCP	prolylcarboxypeptidase (angiotensinase C)	-1,57
651149	ILMN_36491	PREDICTED: Homo sapiens similar to 60S ribosomal protein L3 (L4) (LOC651149), mRNA.	-1,57
644992	ILMN_33996	PREDICTED: Homo sapiens hypothetical LOC644992 (LOC644992), mRNA.	-1,57
402057	ILMN_181541	Homo sapiens similar to 40S ribosomal protein S17 (LOC402057), mRNA.	-1,57

2734	GLG1	golgi glycoprotein 1	-1,57
22934	RPIA	ribose 5-phosphate isomerase A	-1,58
9267	CYTH1	cytohesin 1	-1,58
6159	RPL29	ribosomal protein L29	-1,58
64333	ARHGAP9	Rho GTPase activating protein 9	-1,58
3921	ILMN_27800	Homo sapiens ribosomal protein SA (RPSA), transcript variant 1, mRNA.	-1,59
651436	ILMN_39119	PREDICTED: Homo sapiens similar to ribosomal protein L9 (LOC651436), mRNA.	-1,59
8721	EDF1	endothelial differentiation-related factor 1	-1,59
8906	AP1G2	adaptor-related protein complex 1, gamma 2 subunit	-1,59
6176	RPLP1	ribosomal protein, large, P1	-1,6
3320	HSP90AA1	heat shock protein 90kDa alpha (cytosolic), class A member 1	-1,6
1915	EEF1A1	eukaryotic translation elongation factor 1 alpha 1	-1,6
9219	MTA2	metastasis associated 1 family, member 2	-1,61
644604	EEF1A1P12	eukaryotic translation elongation factor 1 alpha 1 pseudogene 12	-1,61
647856	ILMN_42194	PREDICTED: Homo sapiens similar to 40S ribosomal protein SA (p40) (34/67 kDa laminin receptor) (Colon carcinoma laminin-binding protein) (NEM/1CHD4) (Multidrug resistance-associated protein MGr1-Ag) (LOC647856), mRNA.	-1,61
643949	ILMN_35406	PREDICTED: Homo sapiens similar to 60S acidic ribosomal protein P2 (LOC643949), mRNA.	-1,61
8837	ILMN_4321	Homo sapiens CASP8 and FADD-like apoptosis regulator (CFLAR), mRNA.	-1,61
6430	SRSF5	serine/arginine-rich splicing factor 5	-1,62
390876	ILMN_35719	PREDICTED: Homo sapiens similar to 60S ribosomal protein L35 (LOC390876), mRNA.	-1,62
136143	ILMN_32715	PREDICTED: Homo sapiens similar to ribosomal protein L18 (LOC136143), mRNA.	-1,62
6133	RPL9	ribosomal protein L9	-1,63
6144	RPL21	ribosomal protein L21	-1,63
644604	EEF1A1P12	eukaryotic translation elongation factor 1 alpha 1 pseudogene 12	-1,63
6169	RPL38	ribosomal protein L38	-1,64
650646	ILMN_46687	PREDICTED: Homo sapiens similar to 40S ribosomal protein S26 (LOC650646), mRNA.	-1,64
3326	HSP90AB1	heat shock protein 90kDa alpha (cytosolic), class B member 1	-1,64
10365	KLF2	Kruppel-like factor 2 (lung)	-1,66
1915	EEF1A1	eukaryotic translation elongation factor 1 alpha 1	-1,66
653314	ILMN_31607	Homo sapiens similar to ribosomal protein L19 (LOC653314), mRNA.	-1,67
3939	LDHA	lactate dehydrogenase A	-1,69
3128	HLA-DRB6	major histocompatibility complex, class II, DR beta 6 (pseudogene)	-1,69
400156	ILMN_27668	Homo sapiens 40S ribosomal protein S26-like (RPS26L), non-coding RNA.	-1,7

6155	RPL27	ribosomal protein L27	-1,7
3123	HLA-DRB1	major histocompatibility complex, class II, DR beta 1	-1,7
23524	SRRM2	serine/arginine repetitive matrix 2	-1,71
26135	SERBP1	SERPINE1 mRNA binding protein 1	-1,71
5997	RGS2	regulator of G-protein signaling 2, 24kDa	-1,71
57162	PELI1	pellino homolog 1 (Drosophila)	-1,71
1615	DARS	aspartyl-tRNA synthetase	-1,71
201895	C4orf34	chromosome 4 open reading frame 34	-1,71
5265	SERPINA1	serpin peptidase inhibitor, clade A (alpha-1 antiproteinase, antitrypsin), member 1	-1,72
5265	SERPINA1	serpin peptidase inhibitor, clade A (alpha-1 antiproteinase, antitrypsin), member 1	-1,72
6188	RPS3	ribosomal protein S3	-1,72
6141	RPL18	ribosomal protein L18	-1,72
4714	NDUFB8	NADH dehydrogenase (ubiquinone) 1 beta subcomplex, 8, 19kDa	-1,72
3125	HLA-DRB3	major histocompatibility complex, class II, DR beta 3	-1,72
6194	RPS6	ribosomal protein S6	-1,73
401992	ILMN_138791	PREDICTED: Homo sapiens olfactory receptor, family 2, subfamily T, member 2 (OR2T2), mRNA.	-1,73
645737	ILMN_44612	PREDICTED: Homo sapiens similar to 60S ribosomal protein L7, transcript variant 1 (LOC645737), mRNA.	-1,73
730316	ILMN_28309	PREDICTED: Homo sapiens similar to Nuclear envelope pore membrane protein POM 121 (Pore membrane protein of 121 kDa) (P145) (LOC730316), mRNA.	-1,74
10724	ILMN_11399	Homo sapiens meningioma expressed antigen 5 (hyaluronidase) (MGEA5), mRNA.	-1,75
390354	ILMN_138133	PREDICTED: Homo sapiens similar to ribosomal protein L18a; 60S ribosomal protein L18a, transcript variant 36 (LOC390354), misc RNA.	-1,75
10288	ILMN_180674	Homo sapiens leukocyte immunoglobulin-like receptor, subfamily B (with TM and ITIM domains), member 2 (LILRB2), transcript variant 2, mRNA.	-1,75
6210	RPS15A	ribosomal protein S15a	-1,77
346950	ILMN_36919	PREDICTED: Homo sapiens similar to ribosomal protein L37 (LOC346950), mRNA.	-1,77
	ILMN_72502	Homo sapiens cDNA FLJ31407 fis, clone NT2NE2000137	-1,77
7157	ILMN_11285	Homo sapiens tumor protein p53 (TP53), mRNA.	-1,79
5073	PARN	poly(A)-specific ribonuclease	-1,79
10236	HNRNPR	heterogeneous nuclear ribonucleoprotein R	-1,79
6207	RPS13	ribosomal protein S13	-1,8
10424	PGRMC2	progesterone receptor membrane component 2	-1,8
29997	GLTSCR2	glioma tumor suppressor candidate region gene 2	-1,8

55571	C2orf29	chromosome 2 open reading frame 29	-1,81
6233	RPS27A	ribosomal protein S27a	-1,82
6232	RPS27	ribosomal protein S27	-1,82
654194	ILMN_31988	PREDICTED: Homo sapiens similar to ribosomal protein S27 (LOC654194), mRNA.	-1,82
10146	G3BP1	GTPase activating protein (SH3 domain) binding protein 1	-1,82
6235	RPS29	ribosomal protein S29	-1,83
23163	GGA3	golgi-associated, gamma adaptin ear containing, ARF binding protein 3	-1,83
972	CD74	CD74 molecule, major histocompatibility complex, class II invariant chain	-1,83
6152	RPL24	ribosomal protein L24	-1,84
440733	ILMN_38430	PREDICTED: Homo sapiens similar to 40S ribosomal protein S15 (RIG protein) (LOC440733), mRNA.	-1,84
	ILMN_111893	full-length cDNA clone CS0DI056YK21 of Placenta Cot 25-normalized of Homo sapiens (human)	-1,85
728973	ILMN_38370	PREDICTED: Homo sapiens similar to 40S ribosomal protein S7 (S8) (LOC728973), mRNA.	-1,87
3184	HNRNPD	heterogeneous nuclear ribonucleoprotein D (AU-rich element RNA binding protein 1, 37kDa)	-1,87
6191	RPS4X	ribosomal protein S4, X-linked	-1,88
6209	RPS15	ribosomal protein S15	-1,88
3420	IDH3B	isocitrate dehydrogenase 3 (NAD+) beta	-1,88
2799	GNS	glucosamine (N-acetyl)-6-sulfatase	-1,88
27430	ILMN_18923	Homo sapiens methionine adenosyltransferase II, beta (MAT2B), transcript variant 1, mRNA.	-1,89
23019	CNOT1	CCR4-NOT transcription complex, subunit 1	-1,89
64115	ILMN_174163	Homo sapiens chromosome 10 open reading frame 54 (C10orf54), mRNA.	-1,89
50856	ILMN_8463	Homo sapiens C-type lectin domain family 4, member A (CLEC4A), transcript variant 4, mRNA.	-1,9
6722	ILMN_22299	Homo sapiens serum response factor (c-fos serum response element-binding transcription factor) (SRF), mRNA.	-1,91
2113	ETS1	v-ets erythroblastosis virus E26 oncogene homolog 1 (avian)	-1,91
1974	EIF4A2	eukaryotic translation initiation factor 4A2	-1,91
1937	EEF1G	eukaryotic translation elongation factor 1 gamma	-1,92
648343	ILMN_42525	PREDICTED: Homo sapiens similar to protein phosphatase 1 regulatory subunit 14B (LOC648343), mRNA.	-1,92
6143	RPL19	ribosomal protein L19	-1,94
1654	DDX3X	DEAD (Asp-Glu-Ala-Asp) box polypeptide 3, X-linked	-1,94
6156	RPL30	ribosomal protein L30	-1,95
25832	NBPF14	neuroblastoma breakpoint family, member 14	-1,95
9516	LITAF	lipopolysaccharide-induced TNF factor	-1,97
3646	EIF3E	eukaryotic translation initiation factor 3, subunit E	-1,97
6218	RPS17	ribosomal protein S17	-1,99

643284	ILMN_40380	PREDICTED: Homo sapiens similar to 40S ribosomal protein S29 (LOC643284), mRNA.	-1,99
389672	ILMN_35589	PREDICTED: Homo sapiens similar to 40S ribosomal protein SA (p40) (34/67 kDa laminin receptor) (Colon carcinoma laminin-binding protein) (NEM/1CHD4) (Multidrug resistance-associated protein MGr1-Ag), transcript variant 3 (LOC389672), mRNA.	-2
27335	EIF3K	eukaryotic translation initiation factor 3, subunit K	-2
6165	RPL35A	ribosomal protein L35a	-2,01
647361	ILMN_37525	PREDICTED: Homo sapiens similar to 40S ribosomal protein S29 (LOC647361), mRNA.	-2,01
1350	COX7C	cytochrome c oxidase subunit VIIc	-2,02
6142	RPL18A	ribosomal protein L18a	-2,03
401019	ILMN_35511	PREDICTED: Homo sapiens similar to 40S ribosomal protein S15 (RIG protein), transcript variant 3 (LOC401019), mRNA.	-2,04
6160	RPL31	ribosomal protein L31	-2,08
2219	FCN1	ficolin (collagen/fibrinogen domain containing) 1	-2,09
6235	RPS29	ribosomal protein S29	-2,1
6152	RPL24	ribosomal protein L24	-2,11
6142	RPL18A	ribosomal protein L18a	-2,11
651202	ILMN_45225	PREDICTED: Homo sapiens similar to large subunit ribosomal protein L36a (LOC651202), mRNA.	-2,13
3550	IK	IK cytokine, down-regulator of HLA II	-2,13
8364	HIST1H4C	histone cluster 1, H4c	-2,14
6142	RPL18A	ribosomal protein L18a	-2,16
353514	LILRA5	leukocyte immunoglobulin-like receptor, subfamily A (with TM domain), member 5	-2,17
125144	ILMN_6167	Homo sapiens chromosome 17 open reading frame 45 (C17orf45), mRNA.	-2,17
1937	EEF1G	eukaryotic translation elongation factor 1 gamma	-2,18
79026	AHNAK	AHNAK nucleoprotein	-2,19
652624	ILMN_46806	PREDICTED: Homo sapiens similar to 40S ribosomal protein SA (p40) (34/67 kDa laminin receptor) (LOC652624), mRNA.	-2,21
6122	RPL3	ribosomal protein L3	-2,22
4069	LYZ	lysozyme	-2,22
79081	C11orf48	chromosome 11 open reading frame 48	-2,23
6209	RPS15	ribosomal protein S15	-2,26
6217	RPS16	ribosomal protein S16	-2,26
11180	WDR6	WD repeat domain 6	-2,28
440737	ILMN_39347	PREDICTED: Homo sapiens similar to 60S ribosomal protein L35 (LOC440737), mRNA.	-2,28

3687	ILMN_7741	Homo sapiens integrin, alpha X (complement component 3 receptor 4 subunit) (ITGAX), mRNA.	-2,28
387867	ILMN_34982	PREDICTED: Homo sapiens similar to 40S ribosomal protein SA (p40) (34/67 kDa laminin receptor) (Colon carcinoma laminin-binding protein) (NEM/1CHD4) (Multidrug resistance-associated protein MGr1-Ag) (LOC387867), mRNA.	-2,3
9750	ILMN_21545	Homo sapiens family with sequence similarity 65, member B (FAM65B), transcript variant 2, mRNA.	-2,3
388122	ILMN_37996	PREDICTED: Homo sapiens hypothetical LOC388122 (LOC388122), mRNA.	-2,31
6203	RPS9	ribosomal protein S9	-2,34
81606	LBH	limb bud and heart development homolog (mouse)	-2,35
648249	ILMN_33635	PREDICTED: Homo sapiens similar to 40S ribosomal protein SA (p40) (34/67 kDa laminin receptor) (Colon carcinoma laminin-binding protein) (NEM/1CHD4) (Multidrug resistance-associated protein MGr1-Ag), transcript variant 3 (LOC648249), mRNA.	-2,36
284058	KIAA1267	KIAA1267	-2,37
6203	RPS9	ribosomal protein S9	-2,4
5162	PDHB	pyruvate dehydrogenase (lipoamide) beta	-2,43
11224	RPL35	ribosomal protein L35	-2,48
6170	RPL39	ribosomal protein L39	-2,55
	ILMN_102288	Human mRNA for T-cell specific protein	-2,55
684	BST2	bone marrow stromal cell antigen 2	-2,55
864	RUNX3	runt-related transcription factor 3	-2,57
678	ZFP36L2	zinc finger protein 36, C3H type-like 2	-2,76
6472	SHMT2	serine hydroxymethyltransferase 2 (mitochondrial)	-2,76
7297	TYK2	tyrosine kinase 2	-2,86
4050	LTB	lymphotoxin beta (TNF superfamily, member 3)	-3,19

Supplementary Table S3: List of deregulated KEGG pathways in serum of cancer patients in comparison to healthy indiviaduals (Nsig number of signaficantly changed genes in the pathway; Npath number of genes in the pathway).

Supplementary Table S3.
List of deregulated KEGG pathways in serum of cancer patients in comparison to healthy indiviaduals (N_{sig} number of signaficantly changed genes in the pathway; N_{path} number of genes in the pathway).

KEGG ID	KEGG name	N_{sig}	N_{path}
hsa04610	Complement and coagulation cascades	15	69
hsa00232	Caffeine metabolism	1	7
hsa00983	Drug metabolism - other enzymes	10	52
hsa01100	Metabolic pathways	315	1131
hsa00380	Tryptophan metabolism	10	42
hsa00970	Aminoacyl-tRNA biosynthesis	12	63
hsa00250	Alanine, aspartate and glutamate metabolism	5	32
hsa00280	Valine, leucine and isoleucine degradation	10	44
hsa00410	beta-Alanine metabolism	6	22
hsa00640	Propanoate metabolism	11	32
hsa00650	Butanoate metabolism	8	30
hsa02010	ABC transporters	4	44
hsa04975	Fat digestion and absorption	5	46
hsa04142	Lysosome	39	121
hsa04012	ErbB signaling pathway	33	87
hsa04110	Cell cycle	52	128
hsa04360	Axon guidance	38	130
hsa04722	Neurotrophin signaling pathway	56	127
hsa05130	Pathogenic Escherichia coli infection	38	58
hsa05131	Shigellosis	32	62
hsa05200	Pathways in cancer	114	327
hsa05220	Chronic myeloid leukemia	32	73
hsa05416	Viral myocarditis	36	72
hsa00330	Arginine and proline metabolism	14	54
hsa00340	Histidine metabolism	5	29
hsa00601	Glycosphingolipid biosynthesis - lacto and neolacto series	7	26
hsa00071	Fatty acid metabolism	11	43
hsa01040	Biosynthesis of unsaturated fatty acids	4	21
hsa03320	PPAR signaling pathway	14	70

hsa04146	Peroxisome	20	79
hsa00061	Fatty acid biosynthesis	1	6
hsa00620	Pyruvate metabolism	18	40
hsa04910	Insulin signaling pathway	57	138
hsa04920	Adipocytokine signaling pathway	20	68
hsa00072	Synthesis and degradation of ketone bodies	1	9
hsa00310	Lysine degradation	14	44
hsa00630	Glyoxylate and dicarboxylate metabolism	4	18
hsa00900	Terpenoid backbone biosynthesis	3	15
hsa04742	Taste transduction	9	52
hsa00564	Glycerophospholipid metabolism	14	80
hsa00020	Citrate cycle (TCA cycle)	12	30
hsa00592	alpha-Linolenic acid metabolism	1	20
hsa00740	Riboflavin metabolism	5	11
hsa04520	Adherens junction	34	73
hsa04380	Osteoclast differentiation	52	128
hsa05323	Rheumatoid arthritis	32	92
hsa04270	Vascular smooth muscle contraction	41	116
hsa04145	Phagosome	69	156
hsa04510	Focal adhesion	70	200
hsa04530	Tight junction	50	133
hsa04670	Leukocyte transendothelial migration	45	117
hsa04810	Regulation of actin cytoskeleton	88	214
hsa04971	Gastric acid secretion	28	74
hsa05100	Bacterial invasion of epithelial cells	34	71
hsa05110	Vibrio cholerae infection	26	54
hsa05410	Hypertrophic cardiomyopathy (HCM)	22	83
hsa05412	Arrhythmogenic right ventricular cardiomyopathy (ARVC)	21	74
hsa05414	Dilated cardiomyopathy	24	90
hsa04260	Cardiac muscle contraction	28	77
hsa05146	Amoebiasis	24	106
hsa05322	Systemic lupus erythematosus	49	138
hsa04060	Cytokine-cytokine receptor interaction	50	265
hsa04350	TGF-beta signaling pathway	27	85
hsa00230	Purine metabolism	56	162
hsa05340	Primary immunodeficiency	11	35
hsa05010	Alzheimer's disease	71	168
hsa05120	Epithelial cell signaling in Helicobacter pylori infection	30	68
hsa04623	Cytosolic DNA-sensing pathway	11	56
hsa04020	Calcium signaling pathway	40	177
hsa04062	Chemokine signaling pathway	64	189
hsa04114	Oocyte meiosis	48	114
hsa04540	Gap junction	36	90
hsa04720	Long-term potentiation	27	70

hsa04912	GnRH signaling pathway	33	101
hsa04914	Progesterone-mediated oocyte maturation	35	87
hsa04916	Melanogenesis	33	101
hsa04970	Salivary secretion	26	89
hsa04972	Pancreatic secretion	28	101
hsa04976	Bile secretion	19	71
hsa05142	Chagas disease (American trypanosomiasis)	32	104
hsa04740	Olfactory transduction	45	388
hsa04962	Vasopressin-regulated water reabsorption	18	44
hsa04080	Neuroactive ligand-receptor interaction	37	272
hsa00010	Glycolysis / Gluconeogenesis	30	65
hsa00350	Tyrosine metabolism	6	41
hsa00830	Retinol metabolism	12	64
hsa00980	Metabolism of xenobiotics by cytochrome P450	16	71
hsa00982	Drug metabolism - cytochrome P450	14	73
hsa03410	Base excision repair	12	34
hsa04144	Endocytosis	76	203
hsa05016	Huntington's disease	72	183
hsa00511	Other glycan degradation	1	17
hsa00500	Starch and sucrose metabolism	16	54
hsa04330	Notch signaling pathway	12	47
hsa04614	Renin-angiotensin system	4	17
hsa00260	Glycine, serine and threonine metabolism	5	32
hsa00270	Cysteine and methionine metabolism	12	36
hsa04010	MAPK signaling pathway	95	268
hsa04150	mTOR signaling pathway	20	52
hsa04210	Apoptosis	28	89
hsa04370	VEGF signaling pathway	30	76
hsa04620	Toll-like receptor signaling pathway	30	102
hsa04630	Jak-STAT signaling pathway	51	155
hsa04660	T cell receptor signaling pathway	42	108
hsa04662	B cell receptor signaling pathway	33	75
hsa04664	Fc epsilon RI signaling pathway	30	79
hsa04666	Fc gamma R-mediated phagocytosis	41	95
hsa04973	Carbohydrate digestion and absorption	16	44
hsa05145	Toxoplasmosis	44	133
hsa05160	Hepatitis C	46	135
hsa05210	Colorectal cancer	27	62
hsa05211	Renal cell carcinoma	31	70
hsa05212	Pancreatic cancer	29	70
hsa05213	Endometrial cancer	26	52
hsa05214	Glioma	29	65
hsa05215	Prostate cancer	40	89
hsa05218	Melanoma	23	71

hsa05221	Acute myeloid leukemia	25	58
hsa05222	Small cell lung cancer	29	85
hsa05223	Non-small cell lung cancer	26	54
hsa00860	Porphyrin and chlorophyll metabolism	12	43
hsa04514	Cell adhesion molecules (CAMs)	42	136
hsa00040	Pentose and glucuronate interconversions	8	32
hsa00053	Ascorbate and aldarate metabolism	8	26
hsa00561	Glycerolipid metabolism	12	50
hsa00360	Phenylalanine metabolism	2	17
hsa00030	Pentose phosphate pathway	13	27
hsa00051	Fructose and mannose metabolism	16	36
hsa00052	Galactose metabolism	9	27
hsa00590	Arachidonic acid metabolism	13	59
hsa00591	Linoleic acid metabolism	4	30
hsa04141	Protein processing in endoplasmic reticulum	61	168
hsa00670	One carbon pool by folate	4	18
hsa00910	Nitrogen metabolism	3	23
hsa00480	Glutathione metabolism	20	50
hsa04640	Hematopoietic cell lineage	28	88
hsa05012	Parkinson's disease	60	130
hsa00760	Nicotinate and nicotinamide metabolism	11	24
hsa04115	p53 signaling pathway	28	69
hsa05014	Amyotrophic lateral sclerosis (ALS)	19	54
hsa00240	Pyrimidine metabolism	33	99
hsa04310	Wnt signaling pathway	52	151
hsa05217	Basal cell carcinoma	13	55
hsa04120	Ubiquitin mediated proteolysis	60	139
hsa04621	NOD-like receptor signaling pathway	19	59
hsa04977	Vitamin digestion and absorption	5	24
hsa05143	African trypanosomiasis	9	35
hsa04650	Natural killer cell mediated cytotoxicity	54	136
hsa04940	Type I diabetes mellitus	19	45
hsa05320	Autoimmune thyroid disease	19	54
hsa05330	Allograft rejection	17	39
hsa05332	Graft-versus-host disease	21	43
hsa04964	Proximal tubule bicarbonate reclamation	8	23
hsa04730	Long-term depression	25	70
hsa05219	Bladder cancer	17	42
hsa04710	Circadian rhythm - mammal	5	23
hsa04744	Phototransduction	6	29
hsa00600	Sphingolipid metabolism	13	40
hsa00531	Glycosaminoglycan degradation	5	19
hsa00140	Steroid hormone biosynthesis	9	57
hsa04960	Aldosterone-regulated sodium reabsorption	19	42

hsa04974	Protein digestion and absorption	19	81
hsa00190	Oxidative phosphorylation	62	132
hsa04966	Collecting duct acid secretion	12	27
hsa00300	Lysine biosynthesis	1	3
hsa04612	Antigen processing and presentation	39	78
hsa00120	Primary bile acid biosynthesis	4	16
hsa00430	Taurine and hypotaurine metabolism	1	10
hsa05020	Prion diseases	12	36
hsa00290	Valine, leucine and isoleucine biosynthesis	3	11
hsa00770	Pantothenate and CoA biosynthesis	6	16
hsa05216	Thyroid cancer	16	29
hsa04672	Intestinal immune network for IgA production	12	49
hsa05150	Staphylococcus aureus infection	18	56
hsa03440	Homologous recombination	10	28
hsa04340	Hedgehog signaling pathway	14	56
hsa03020	RNA polymerase	8	29
hsa04130	SNARE interactions in vesicular transport	17	36
hsa00780	Biotin metabolism	1	2
hsa03018	RNA degradation	35	71
hsa05140	Leishmaniasis	33	73
hsa04930	Type II diabetes mellitus	13	48
hsa04070	Phosphatidylinositol signaling system	17	78
hsa04622	RIG-I-like receptor signaling pathway	14	71
hsa00450	Selenocompound metabolism	5	17
hsa03420	Nucleotide excision repair	16	45
hsa04512	ECM-receptor interaction	17	85
hsa05144	Malaria	9	51
hsa05310	Asthma	11	31
hsa03040	Spliceosome	68	128
hsa00100	Steroid biosynthesis	5	19
hsa00520	Amino sugar and nucleotide sugar metabolism	15	48
hsa03013	RNA transport	66	152
hsa03008	Ribosome biogenesis in eukaryotes	29	81
hsa03015	mRNA surveillance pathway	34	83
hsa00510	N-Glycan biosynthesis	10	49
hsa03030	DNA replication	16	36
hsa03450	Non-homologous end-joining	4	14
hsa00062	Fatty acid elongation in mitochondria	2	8
hsa04320	Dorso-ventral axis formation	10	25
hsa00534	Glycosaminoglycan biosynthesis - heparan sulfate	2	26
hsa03010	Ribosome	70	92
hsa04950	Maturity onset diabetes of the young	3	25
hsa00603	Glycosphingolipid biosynthesis - globo series	3	14
hsa00514	Other types of O-glycan biosynthesis	9	46

hsa00533	Glycosaminoglycan biosynthesis - keratan sulfate	4	15
hsa00604	Glycosphingolipid biosynthesis - ganglio series	1	15
hsa00512	Mucin type O-Glycan biosynthesis	5	30
hsa00524	Butirosin and neomycin biosynthesis	2	5
hsa00460	Cyanoamino acid metabolism	2	7
hsa00471	D-Glutamine and D-glutamate metabolism	1	4
hsa00400	Phenylalanine, tyrosine and tryptophan biosynthesis	1	5
hsa00563	Glycosylphosphatidylinositol(GPI)-anchor biosynthesis	3	25
hsa03430	Mismatch repair	9	23
hsa03022	Basal transcription factors	16	37
hsa03060	Protein export	11	23
hsa04140	Regulation of autophagy	7	34
hsa03050	Proteasome	28	45
hsa00562	Inositol phosphate metabolism	11	57
hsa04122	Sulfur relay system	3	10
hsa00565	Ether lipid metabolism	8	36
hsa00920	Sulfur metabolism	1	13
hsa00730	Thiamine metabolism	1	4
hsa00532	Glycosaminoglycan biosynthesis - chondroitin sulfate	3	22
hsa00785	Lipoic acid metabolism	1	3
hsa00790	Folate biosynthesis	1	11
hsa00750	Vitamin B6 metabolism	1	6

Supplementary Table S4: GSEA on GO terms - biological process of serum specimens of cancer patients in comparison to healthy individuals shows significant deregulation in translational processes, ribosomal biogenesis, viral transcription, negative regulation of DNA damage response - signal transduction by p53 class mediator, induction of apoptosis (showed only GO terms with p-value < 0.05).

Supplementary Table S4.

GSEA on GO terms - biological process of serum specimens of cancer patients in comparison to healthy individuals shows significant deregulation in translational processes, ribosomal biogenesis, viral transcription, negative regulation of DNA damage response - signal transduction by p53 class mediator, induction of apoptosis (showed only GO terms with p-value < 0.05).

GO ID	GO term	p-value
GO:0006414	translational elongation	1,20E-63
GO:0006415	translational termination	1,50E-62
GO:0006613	cotranslational protein targeting to membrane	1,90E-57
GO:0006614	SRP-dependent cotranslational protein targeting to membrane	1,90E-57
GO:0045047	protein targeting to ER	8,40E-57
GO:0072599	establishment of protein localization to endoplasmic reticulum	8,40E-57
GO:0043624	cellular protein complex disassembly	2,30E-54
GO:0070972	protein localization to endoplasmic reticulum	2,30E-54
GO:0043241	protein complex disassembly	7,80E-54
GO:0000184	nuclear-transcribed mRNA catabolic process, nonsense-mediated decay	9,40E-54
GO:0006402	mRNA catabolic process	4,20E-52
GO:0034623	cellular macromolecular complex disassembly	1,00E-51
GO:0000956	nuclear-transcribed mRNA catabolic process	2,10E-51
GO:0032984	macromolecular complex disassembly	2,80E-51
GO:0006612	protein targeting to membrane	7,20E-51
GO:0006401	RNA catabolic process	1,70E-50
GO:0006413	translational initiation	3,20E-50
GO:0019080	viral genome expression	4,30E-50
GO:0019083	viral transcription	4,30E-50
GO:0072594	establishment of protein localization to organelle	1,70E-44
GO:0016071	mRNA metabolic process	2,20E-39
GO:0071845	cellular component disassembly at cellular level	2,10E-38
GO:0022411	cellular component disassembly	2,80E-38
GO:0019058	viral infectious cycle	4,00E-37
GO:0022415	viral reproductive process	2,00E-36
GO:0006412	translation	1,70E-32
GO:0071822	protein complex subunit organization	1,50E-28
GO:0034621	cellular macromolecular complex subunit organization	1,70E-28

GO:0048610	cellular process involved in reproduction	4,10E-28
GO:0044265	cellular macromolecule catabolic process	4,70E-27
GO:0016032	viral reproduction	1,00E-26
GO:0006605	protein targeting	5,00E-26
GO:0009057	macromolecule catabolic process	1,70E-25
GO:0033365	protein localization to organelle	1,80E-25
GO:0043933	macromolecular complex subunit organization	8,40E-25
GO:0006886	intracellular protein transport	3,40E-23
GO:0070727	cellular macromolecule localization	7,10E-20
GO:0034613	cellular protein localization	8,30E-20
GO:0022414	reproductive process	2,00E-16
GO:0000003	reproduction	3,80E-16
GO:0015031	protein transport	1,10E-15
GO:0044248	cellular catabolic process	2,20E-15
GO:0045184	establishment of protein localization	7,70E-15
GO:0009056	catabolic process	3,20E-14
GO:0008104	protein localization	5,10E-14
GO:0046907	intracellular transport	6,90E-14
GO:0033036	macromolecule localization	1,70E-13
GO:0051649	establishment of localization in cell	7,30E-13
GO:0042274	ribosomal small subunit biogenesis	1,50E-12
GO:0016072	rRNA metabolic process	9,00E-12
GO:0051641	cellular localization	1,20E-11
GO:0006364	rRNA processing	1,40E-11
GO:0019538	protein metabolic process	5,80E-11
GO:0042254	ribosome biogenesis	7,80E-11
GO:0044267	cellular protein metabolic process	2,30E-10
GO:0006396	RNA processing	3,60E-10
GO:0034470	ncRNA processing	5,00E-10
GO:0010467	gene expression	1,50E-09
GO:0016070	RNA metabolic process	4,80E-09
GO:0034660	ncRNA metabolic process	6,30E-09
GO:0071840	cellular component organization or biogenesis	1,50E-08
GO:0071843	cellular component biogenesis at cellular level	1,60E-08
GO:0043170	macromolecule metabolic process	1,70E-08
GO:0071841	cellular component organization or biogenesis at cellular level	2,20E-08
GO:0022613	ribonucleoprotein complex biogenesis	3,80E-08
GO:0016043	cellular component organization	6,40E-08
GO:0071842	cellular component organization at cellular level	1,20E-07
GO:0090304	nucleic acid metabolic process	1,40E-07
GO:0042273	ribosomal large subunit biogenesis	3,60E-07
GO:0044260	cellular macromolecule metabolic process	6,30E-07
GO:0000028	ribosomal small subunit assembly	7,00E-07
GO:0044085	cellular component biogenesis	3,90E-06
GO:0006807	nitrogen compound metabolic process	5,90E-06
GO:0034641	cellular nitrogen compound metabolic process	6,40E-06
GO:0006139	nucleobase-containing compound metabolic process	1,40E-05

GO:0051234	establishment of localization	2,30E-05
GO:0032774	RNA biosynthetic process	3,00E-05
GO:0044238	primary metabolic process	4,10E-05
GO:0001775	cell activation	4,90E-05
GO:0006810	transport	4,90E-05
GO:0009059	macromolecule biosynthetic process	5,90E-05
GO:0044237	cellular metabolic process	5,90E-05
GO:0007229	integrin-mediated signaling pathway	8,50E-05
GO:0051179	localization	8,60E-05
GO:0044249	cellular biosynthetic process	0,00013
GO:0006955	immune response	0,00014
GO:0009058	biosynthetic process	0,00014
GO:0034645	cellular macromolecule biosynthetic process	0,00016
GO:0002376	immune system process	0,00017
GO:0008152	metabolic process	0,00019
GO:0006397	mRNA processing	2,00E-04
GO:0042060	wound healing	0,00029
GO:0042255	ribosome assembly	0,00033
GO:0051085	chaperone mediated protein folding requiring cofactor	0,00038
GO:0051293	establishment of spindle localization	0,00046
GO:0002576	platelet degranulation	0,00058
GO:0046649	lymphocyte activation	6,00E-04
GO:0006458	'de novo' protein folding	0,00073
GO:0030330	DNA damage response, signal transduction by p53 class mediator	0,00074
GO:0072331	signal transduction by p53 class mediator	0,00074
GO:2001021	negative regulation of response to DNA damage stimulus	0,00075
GO:0042110	T cell activation	0,00077
GO:0048872	homeostasis of number of cells	0,00088
GO:0007596	blood coagulation	0,0011
GO:0010942	positive regulation of cell death	0,0011
GO:0007599	hemostasis	0,0012
GO:0050817	coagulation	0,0012
GO:0065008	regulation of biological quality	0,0013
GO:0043065	positive regulation of apoptotic process	0,0014
GO:0043068	positive regulation of programmed cell death	0,0015
GO:0051653	spindle localization	0,0015
GO:0045321	leukocyte activation	0,0018
GO:0002468	dendritic cell antigen processing and presentation	0,0019
GO:0002604	regulation of dendritic cell antigen processing and presentation	0,0019
GO:0030593	neutrophil chemotaxis	0,0019
GO:0031124	mRNA 3'-end processing	0,0019
GO:0000375	RNA splicing, via transesterification reactions	0,002
GO:0002456	T cell mediated immunity	0,0021
GO:0042770	signal transduction in response to DNA damage	0,0023
GO:0008380	RNA splicing	0,0024
GO:0043518	negative regulation of DNA damage response, signal transduction by p53 class mediator	0,0024

GO:0010941	regulation of cell death	0,0027
GO:0035872	nucleotide-binding domain, leucine rich repeat containing receptor signaling pathway	0,0027
GO:0038032	termination of G-protein coupled receptor signaling pathway	0,003
GO:0070663	regulation of leukocyte proliferation	0,0032
GO:0043516	regulation of DNA damage response, signal transduction by p53 class mediator	0,0033
GO:0002504	antigen processing and presentation of peptide or polysaccharide antigen via MHC class II	0,0034
GO:0001959	regulation of cytokine-mediated signaling pathway	0,0035
GO:0031123	RNA 3'-end processing	0,0036
GO:0065003	macromolecular complex assembly	0,0037
GO:0090022	regulation of neutrophil chemotaxis	0,0037
GO:0006917	induction of apoptosis	0,0039
GO:0012502	induction of programmed cell death	0,0039
GO:0006915	apoptotic process	0,004
GO:0022607	cellular component assembly	0,004
GO:0050730	regulation of peptidyl-tyrosine phosphorylation	0,004
GO:0000377	RNA splicing, via transesterification reactions with bulged adenosine as nucleophile	0,0042
GO:0000398	nuclear mRNA splicing, via spliceosome	0,0042
GO:0009611	response to wounding	0,0042
GO:0042981	regulation of apoptotic process	0,0042
GO:0055072	iron ion homeostasis	0,0042
GO:0032944	regulation of mononuclear cell proliferation	0,0046
GO:0050670	regulation of lymphocyte proliferation	0,0046
GO:0006457	protein folding	0,0047
GO:0060338	regulation of type I interferon-mediated signaling pathway	0,0048
GO:0002520	immune system development	0,0051
GO:0043067	regulation of programmed cell death	0,0052
GO:0034101	erythrocyte homeostasis	0,0053
GO:0042026	protein refolding	0,0053
GO:0048144	fibroblast proliferation	0,0053
GO:0048145	regulation of fibroblast proliferation	0,0053
GO:0019882	antigen processing and presentation	0,0054
GO:0002577	regulation of antigen processing and presentation	0,0055
GO:0019886	antigen processing and presentation of exogenous peptide antigen via MHC class II	0,0055
GO:0060547	negative regulation of necrotic cell death	0,0055
GO:0090023	positive regulation of neutrophil chemotaxis	0,0055
GO:0090303	positive regulation of wound healing	0,0055
GO:0010740	positive regulation of intracellular protein kinase cascade	0,0056
GO:0009987	cellular process	0,0061
GO:0051249	regulation of lymphocyte activation	0,0065
GO:0031571	mitotic cell cycle G1/S transition DNA damage checkpoint	0,0067
GO:0070661	leukocyte proliferation	0,0067
GO:0012501	programmed cell death	0,0068

GO:0002429	immune response-activating cell surface receptor signaling pathway	0,0069
GO:0000288	nuclear-transcribed mRNA catabolic process, deadenylation-dependent decay	0,0073
GO:0043487	regulation of RNA stability	0,0073
GO:0051704	multi-organism process	0,0073
GO:0050776	regulation of immune response	0,0074
GO:0050867	positive regulation of cell activation	0,0075
GO:0000132	establishment of mitotic spindle orientation	0,0077
GO:0002579	positive regulation of antigen processing and presentation	0,0077
GO:0002606	positive regulation of dendritic cell antigen processing and presentation	0,0077
GO:0006878	cellular copper ion homeostasis	0,0077
GO:0006880	intracellular sequestering of iron ion	0,0077
GO:0040001	establishment of mitotic spindle localization	0,0077
GO:0045061	thymic T cell selection	0,0077
GO:0051294	establishment of spindle orientation	0,0077
GO:0006977	DNA damage response, signal transduction by p53 class mediator resulting in cell cycle arrest	0,0078
GO:0032943	mononuclear cell proliferation	0,0078
GO:0046651	lymphocyte proliferation	0,0078
GO:0072395	signal transduction involved in cell cycle checkpoint	0,0078
GO:0072401	signal transduction involved in DNA integrity checkpoint	0,0078
GO:0072404	signal transduction involved in G1/S transition checkpoint	0,0078
GO:0072413	signal transduction involved in mitotic cell cycle checkpoint	0,0078
GO:0072422	signal transduction involved in DNA damage checkpoint	0,0078
GO:0072431	signal transduction involved in mitotic cell cycle G1/S transition DNA damage checkpoint	0,0078
GO:0072474	signal transduction involved in mitotic cell cycle G1/S checkpoint	0,0078
GO:0044419	interspecies interaction between organisms	0,0079
GO:0000289	nuclear-transcribed mRNA poly(A) tail shortening	0,008
GO:0050851	antigen receptor-mediated signaling pathway	0,008
GO:0050865	regulation of cell activation	0,0081
GO:0002694	regulation of leukocyte activation	0,0082
GO:0030595	leukocyte chemotaxis	0,0082
GO:0042100	B cell proliferation	0,0082
GO:0051084	'de novo' posttranslational protein folding	0,0082
GO:0002768	immune response-regulating cell surface receptor signaling pathway	0,0083
GO:0002478	antigen processing and presentation of exogenous peptide antigen	0,0084
GO:0019884	antigen processing and presentation of exogenous antigen	0,0084
GO:0031575	mitotic cell cycle G1/S transition checkpoint	0,0084
GO:0050852	T cell receptor signaling pathway	0,0084
GO:0002696	positive regulation of leukocyte activation	0,0086
GO:0023021	termination of signal transduction	0,0087
GO:0048147	negative regulation of fibroblast proliferation	0,0087
GO:0061077	chaperone-mediated protein folding	0,0087
GO:0006826	iron ion transport	0,0092
GO:0018212	peptidyl-tyrosine modification	0,0092

GO:0030010	establishment of cell polarity	0,0092
GO:0040017	positive regulation of locomotion	0,0097
GO:0050870	positive regulation of T cell activation	0,0097
GO:0051272	positive regulation of cellular component movement	0,0098
GO:0030097	hemopoiesis	0,01
GO:0050731	positive regulation of peptidyl-tyrosine phosphorylation	0,01
GO:0071158	positive regulation of cell cycle arrest	0,01
GO:0002449	lymphocyte mediated immunity	0,011
GO:0006935	chemotaxis	0,011
GO:0018108	peptidyl-tyrosine phosphorylation	0,011
GO:0042330	taxis	0,011
GO:0048523	negative regulation of cellular process	0,011
GO:0050863	regulation of T cell activation	0,011
GO:0043383	negative T cell selection	0,012
GO:0045060	negative thymic T cell selection	0,012
GO:0050878	regulation of body fluid levels	0,012
GO:0002764	immune response-regulating signaling pathway	0,013
GO:0006767	water-soluble vitamin metabolic process	0,013
GO:0007159	leukocyte cell-cell adhesion	0,013
GO:0007416	synapse assembly	0,013
GO:0034622	cellular macromolecular complex assembly	0,013
GO:0051439	regulation of ubiquitin-protein ligase activity involved in mitotic cell cycle	0,013
GO:0001913	T cell mediated cytotoxicity	0,014
GO:0002544	chronic inflammatory response	0,014
GO:0007163	establishment or maintenance of cell polarity	0,014
GO:0007632	visual behavior	0,014
GO:0008088	axon cargo transport	0,014
GO:0010611	regulation of cardiac muscle hypertrophy	0,014
GO:0010939	regulation of necrotic cell death	0,014
GO:0014743	regulation of muscle hypertrophy	0,014
GO:0043502	regulation of muscle adaptation	0,014
GO:0045058	T cell selection	0,014
GO:0048534	hemopoietic or lymphoid organ development	0,014
GO:0050803	regulation of synapse structure and activity	0,014
GO:0051325	interphase	0,014
GO:0051897	positive regulation of protein kinase B signaling cascade	0,014
GO:0002757	immune response-activating signal transduction	0,015
GO:0008219	cell death	0,015
GO:0030335	positive regulation of cell migration	0,015
GO:0071779	G1/S transition checkpoint	0,015
GO:2000147	positive regulation of cell motility	0,015
GO:0030168	platelet activation	0,016
GO:0043488	regulation of mRNA stability	0,016
GO:0016265	death	0,017
GO:0070664	negative regulation of leukocyte proliferation	0,017
GO:0008277	regulation of G-protein coupled receptor protein signaling pathway	0,018

GO:0051251	positive regulation of lymphocyte activation	0,018
GO:0060759	regulation of response to cytokine stimulus	0,018
GO:0006026	aminoglycan catabolic process	0,019
GO:0006461	protein complex assembly	0,019
GO:0007183	SMAD protein complex assembly	0,019
GO:0043249	erythrocyte maturation	0,019
GO:0051168	nuclear export	0,019
GO:0055070	copper ion homeostasis	0,019
GO:0061001	regulation of dendritic spine morphogenesis	0,019
GO:0030890	positive regulation of B cell proliferation	0,02
GO:0043500	muscle adaptation	0,02
GO:0045429	positive regulation of nitric oxide biosynthetic process	0,02
GO:0070271	protein complex biogenesis	0,02
GO:2000045	regulation of G1/S transition of mitotic cell cycle	0,02
GO:0006887	exocytosis	0,021
GO:0002690	positive regulation of leukocyte chemotaxis	0,022
GO:0050854	regulation of antigen receptor-mediated signaling pathway	0,022
GO:0050921	positive regulation of chemotaxis	0,022
GO:0051329	interphase of mitotic cell cycle	0,022
GO:0051437	positive regulation of ubiquitin-protein ligase activity involved in mitotic cell cycle	0,022
GO:0001516	prostaglandin biosynthetic process	0,023
GO:0002495	antigen processing and presentation of peptide antigen via MHC class II	0,023
GO:0007176	regulation of epidermal growth factor-activated receptor activity	0,023
GO:0008306	associative learning	0,023
GO:0010613	positive regulation of cardiac muscle hypertrophy	0,023
GO:0010677	negative regulation of cellular carbohydrate metabolic process	0,023
GO:0014742	positive regulation of muscle hypertrophy	0,023
GO:0042113	B cell activation	0,023
GO:0042127	regulation of cell proliferation	0,023
GO:0045912	negative regulation of carbohydrate metabolic process	0,023
GO:0046457	prostanoid biosynthetic process	0,023
GO:0071844	cellular component assembly at cellular level	0,023
GO:0002688	regulation of leukocyte chemotaxis	0,025
GO:0032945	negative regulation of mononuclear cell proliferation	0,025
GO:0042116	macrophage activation	0,025
GO:0042592	homeostatic process	0,025
GO:0050672	negative regulation of lymphocyte proliferation	0,025
GO:0051896	regulation of protein kinase B signaling cascade	0,025
GO:0070374	positive regulation of ERK1 and ERK2 cascade	0,025
GO:0031145	anaphase-promoting complex-dependent proteasomal ubiquitin-dependent protein catabolic process	0,026
GO:0051270	regulation of cellular component movement	0,026
GO:0007093	mitotic cell cycle checkpoint	0,027
GO:0090068	positive regulation of cell cycle process	0,027
GO:0043484	regulation of RNA splicing	0,028

GO:0048520	positive regulation of behavior	0,028
GO:0006120	mitochondrial electron transport, NADH to ubiquinone	0,029
GO:0009451	RNA modification	0,029
GO:0010608	posttranscriptional regulation of gene expression	0,029
GO:0030888	regulation of B cell proliferation	0,029
GO:0042129	regulation of T cell proliferation	0,029
GO:0045744	negative regulation of G-protein coupled receptor protein signaling pathway	0,029
GO:0002682	regulation of immune system process	0,03
GO:0006518	peptide metabolic process	0,03
GO:0051438	regulation of ubiquitin-protein ligase activity	0,031
GO:0002250	adaptive immune response	0,032
GO:0002709	regulation of T cell mediated immunity	0,032
GO:0006692	prostanoid metabolic process	0,032
GO:0045931	positive regulation of mitotic cell cycle	0,032
GO:0050920	regulation of chemotaxis	0,032
GO:0006405	RNA export from nucleus	0,033
GO:0008344	adult locomotory behavior	0,034
GO:0003300	cardiac muscle hypertrophy	0,035
GO:0006508	proteolysis	0,035
GO:0014897	striated muscle hypertrophy	0,035
GO:0051352	negative regulation of ligase activity	0,035
GO:0051436	negative regulation of ubiquitin-protein ligase activity involved in mitotic cell cycle	0,035
GO:0051444	negative regulation of ubiquitin-protein ligase activity	0,035
GO:0070265	necrotic cell death	0,035
GO:0022618	ribonucleoprotein complex assembly	0,036
GO:0071826	ribonucleoprotein complex subunit organization	0,036
GO:0051340	regulation of ligase activity	0,037
GO:0002551	mast cell chemotaxis	0,038
GO:0002687	positive regulation of leukocyte migration	0,038
GO:0033119	negative regulation of RNA splicing	0,038
GO:0033631	cell-cell adhesion mediated by integrin	0,038
GO:0035020	regulation of Rac protein signal transduction	0,038
GO:0045059	positive thymic T cell selection	0,038
GO:0060997	dendritic spine morphogenesis	0,038
GO:0060998	regulation of dendritic spine development	0,038
GO:0071385	cellular response to glucocorticoid stimulus	0,038
GO:0072378	blood coagulation, fibrin clot formation	0,038
GO:2001237	negative regulation of extrinsic apoptotic signaling pathway	0,038
GO:0002443	leukocyte mediated immunity	0,041
GO:0000216	M/G1 transition of mitotic cell cycle	0,042
GO:0060326	cell chemotaxis	0,042
GO:0002460	adaptive immune response based on somatic recombination of immune receptors built from immunoglobulin superfamily domains	0,043
GO:0030334	regulation of cell migration	0,043
GO:0006879	cellular iron ion homeostasis	0,044

GO:0051494	negative regulation of cytoskeleton organization	0,044
GO:0030837	negative regulation of actin filament polymerization	0,045
GO:0033261	regulation of S phase	0,045
GO:0071260	cellular response to mechanical stimulus	0,045
GO:0006952	defense response	0,046
GO:0045333	cellular respiration	0,046
GO:0006644	phospholipid metabolic process	0,047
GO:0048002	antigen processing and presentation of peptide antigen	0,047
GO:0006417	regulation of translation	0,048
GO:0007160	cell-matrix adhesion	0,049
GO:0042590	antigen processing and presentation of exogenous peptide antigen via MHC class I	0,049
GO:0043491	protein kinase B signaling cascade	0,049
GO:0050778	positive regulation of immune response	0,049

Supplementary Table S5: GSEA on GO terms - cytological compartment of serum specimens of cancer patients in comparison to healthy individuals shows changes mainly in cytosolic or nuclear compartment (showed only GO terms with p-value < 0.05).

Supplementary Table S5.
GSEA on GO terms - cytological compartment of serum specimens of cancer patients in comparison to healthy individuals shows changes mainly in cytosolic or nuclear compartment (showed only GO terms with p-value < 0.05).

GO ID	GO term	p-value
GO:0022626	cytosolic ribosome	4,20E-63
GO:0044391	ribosomal subunit	2,00E-57
GO:0044445	cytosolic part	2,30E-52
GO:0005840	ribosome	1,40E-50
GO:0030529	ribonucleoprotein complex	1,40E-36
GO:0022627	cytosolic small ribosomal subunit	7,90E-34
GO:0022625	cytosolic large ribosomal subunit	7,10E-32
GO:0015934	large ribosomal subunit	3,20E-30
GO:0015935	small ribosomal subunit	4,10E-27
GO:0005829	cytosol	1,80E-22
GO:0032991	macromolecular complex	2,00E-19
GO:0044444	cytoplasmic part	1,90E-12
GO:0043228	non-membrane-bounded organelle	7,80E-12
GO:0043232	intracellular non-membrane-bounded organelle	7,80E-12
GO:0005737	cytoplasm	2,50E-09
GO:0044422	organelle part	1,00E-07
GO:0044446	intracellular organelle part	1,30E-07
GO:0044424	intracellular part	2,20E-07
GO:0005622	intracellular	1,10E-06
GO:0043226	organelle	3,00E-06
GO:0043229	intracellular organelle	3,30E-06
GO:0005853	eukaryotic translation elongation factor 1 complex	2,00E-05
GO:0008305	integrin complex	0,00017
GO:0005730	nucleolus	3,00E-04
GO:0031093	platelet alpha granule lumen	0,00039
GO:0034774	secretory granule lumen	0,00039
GO:0031091	platelet alpha granule	0,00047
GO:0005623	cell	0,00057
GO:0044464	cell part	0,00057
GO:0031983	vesicle lumen	0,00089
GO:0060205	cytoplasmic membrane-bounded vesicle lumen	0,00089
GO:0044452	nucleolar part	0,0013
GO:0031974	membrane-enclosed lumen	0,0026

GO:0005681	spliceosomal complex	0,0027
GO:0043233	organelle lumen	0,0037
GO:0043235	receptor complex	0,0038
GO:0001726	ruffle	0,0041
GO:0071013	catalytic step 2 spliceosome	0,0041
GO:0042613	MHC class II protein complex	0,0044
GO:0032587	ruffle membrane	0,0046
GO:0008180	signalosome	0,0058
GO:0044428	nuclear part	0,0064
GO:0005742	mitochondrial outer membrane translocase complex	0,0069
GO:0031594	neuromuscular junction	0,0069
GO:0005771	multivesicular body	0,0073
GO:0016604	nuclear body	0,009
GO:0045211	postsynaptic membrane	0,011
GO:0005852	eukaryotic translation initiation factor 3 complex	0,012
GO:0005868	cytoplasmic dynein complex	0,012
GO:0005912	adherens junction	0,012
GO:0031256	leading edge membrane	0,014
GO:0031981	nuclear lumen	0,014
GO:0070161	anchoring junction	0,014
GO:0042611	MHC protein complex	0,018
GO:0030054	cell junction	0,019
GO:0043209	myelin sheath	0,019
GO:0044291	cell-cell contact zone	0,019
GO:0070013	intracellular organelle lumen	0,019
GO:0005924	cell-substrate adherens junction	0,02
GO:0016272	prefoldin complex	0,02
GO:0030055	cell-substrate junction	0,02
GO:0001725	stress fiber	0,021
GO:0005747	mitochondrial respiratory chain complex I	0,021
GO:0030018	Z disc	0,021
GO:0030964	NADH dehydrogenase complex	0,021
GO:0032432	actin filament bundle	0,021
GO:0045271	respiratory chain complex I	0,021
GO:0045121	membrane raft	0,026
GO:0005925	focal adhesion	0,027
GO:0009986	cell surface	0,027
GO:0030141	secretory granule	0,027
GO:0015030	Cajal body	0,033
GO:0008043	intracellular ferritin complex	0,034
GO:0008290	F-actin capping protein complex	0,034
GO:0030056	hemidesmosome	0,034
GO:0070288	ferritin complex	0,034
GO:0009897	external side of plasma membrane	0,037
GO:0009295	nucleoid	0,039
GO:0042645	mitochondrial nucleoid	0,039
GO:0005604	basement membrane	0,045

GO:0014704	intercalated disc	0,045
